# Cross-Cultural Differences and Similarities in Human Value Instantiation

**DOI:** 10.3389/fpsyg.2018.00849

**Published:** 2018-05-29

**Authors:** Paul H. P. Hanel, Gregory R. Maio, Ana K. S. Soares, Katia C. Vione, Gabriel L. de Holanda Coelho, Valdiney V. Gouveia, Appasaheb C. Patil, Shanmukh V. Kamble, Antony S. R. Manstead

**Affiliations:** ^1^School of Psychology, Cardiff University, Cardiff, United Kingdom; ^2^Department of Psychology, University of Bath, Bath, United Kingdom; ^3^Departamento de Psicologia, Universidade Federal de Mato Grosso do Sul, Campo Grande, Brazil; ^4^Department of Psychology, University of Derby, Derby, United Kingdom; ^5^Departamento de Psicologia, Universidade Federal da Paraíba, Joao Pessoa, Brazil; ^6^Department of Psychology, Karnatak University, Dharwad, India

**Keywords:** human values, instantiation, cross-cultural, value-behavior relations, similarities, differences

## Abstract

Previous research found that the within-country variability of human values (e.g., equality and helpfulness) clearly outweighs between-country variability. Across three countries (Brazil, India, and the United Kingdom), the present research tested in student samples whether between-nation differences reside more in the behaviors used to concretely instantiate (i.e., exemplify or understand) values than in their importance as abstract ideals. In Study 1 (*N* = 630), we found several meaningful between-country differences in the behaviors that were used to concretely instantiate values, alongside high within-country variability. In Study 2 (*N* = 677), we found that participants were able to match instantiations back to the values from which they were derived, even if the behavior instantiations were spontaneously produced only by participants from another country or were created by us. Together, these results support the hypothesis that people in different nations can differ in the behaviors that are seen as typical as instantiations of values, while holding similar ideas about the abstract meaning of the values and their importance.

## Introduction

In recent years, many Western countries have accepted once again tens or even hundreds of thousands of immigrants into their country. This has sparked widespread discussions of how well immigrants are able to acculturate (e.g., The Economist, 2016). For example, a recent Canadian survey found that three quarters of Ontarians feel that Muslim immigrants have fundamentally different values than themselves (Keung, 2016). This feeling is in contrast to large international surveys of human values in which it was found that people from more than 55 nations are consistent in valuing some values more and others less (Schwartz and Bardi, 2001). How then is it the case that people from different countries appear to be so different? The present research follows up this train of thought by testing whether people in different nations differ in the *behaviors* that are seen as typical instantiations (i.e., examples) of values, while holding similar ideas about the abstract meaning of the values and their importance.

### Conceptualizing Values and Value Differences

**FIGURE 1:**
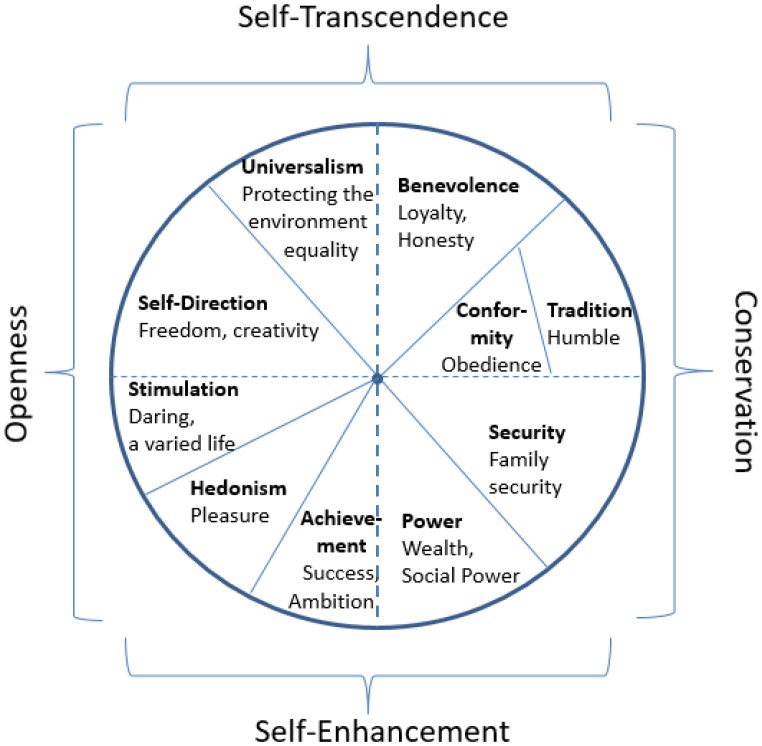
Schwartz’ (1992) circumplex model of human values displaying 10 value types (bold font) and examples of values in each type (normal font) along two dimensions.

Values, abstract guiding principles, have gained a lot of attention, not just within psychology, but also in neighboring fields such as sociology, economics, philosophy, and political science (Schwartz, 1992; Gouveia, 2013; Maio, 2016). In the last three decades, researchers have asked people to rate diverse values in terms of their importance as guiding principles in their lives. Analyses of these ratings have taught us that the structure of human values is very similar across more than 80 countries (Schwartz, 1992; Bilsky et al., 2011; Schwartz et al., 2012). That is, the same values have been grouped together in most countries, resulting in the view that values within a cluster are motivationally compatible. More specifically, in the predominant value model (Schwartz, 1992) 10 value types are distinguished: power, achievement, hedonism, stimulation, self-direction, universalism, benevolence, tradition, conformity, and security. The 10 value types can be combined into four higher order value types, which form the endpoints of two orthogonal dimensions: openness values vs. conservation values, and self-transcendence values vs. self-enhancement values (see **Figure 1**). Adjacent value types are motivationally compatible and hence positively correlated, whereas opposing value types are expected to be motivationally incompatible and negatively related.

In addition, there is similarity in value hierarchies (Schwartz and Bardi, 2001). Benevolence, universalism, and self-direction values are regarded as the most important across more than 50 countries, whereas tradition and power are valued least. Country of origin explains on average only 2–12% of inter-individual variance (Fischer and Schwartz, 2011). Thus, there is high consensus on value priorities across countries.

Given these findings, how is it that people often persist in believing that people from different countries hold different values? Some factors are likely to be motivational: abundant evidence points to the roles of realistic group conflict (Bobo, 1983), social identification (Tajfel and Turner, 1986), symbolic racism (Kinder and Sears, 1981), and various biases (e.g., symbolic self-completion, Gollwitzer et al., 1982, or system justification, Jost and Banaji, 1994) that can lead us to feel that our own group is superior to other groups in numerous characteristics, including values. Other factors are cognitive: social learning (Bandura et al., 1961) and stereotyping processes (e.g., illusory correlation, Hamilton and Rose, 1980) may lead us to encode other groups’ characteristics in ways that magnify the differences between groups. More relevant to the present research, however, is the nature of the values concept itself. Specifically, as abstract ideals, values subsume a wide range of behaviors as exemplars of the concepts. People may perceive differences between social groups because of the differences between groups in the specific behaviors that are seen as exemplars of different values, even if other behaviors that are exemplars of the values do not differ. Thus, by thinking about groups in terms of concrete instances, differences may be stronger than similarities.

In other words, people in different social groups may endorse the same values but associate different behaviors with them (Maio, 2010). For example, the value of equality may be linked to comparisons between men and women in countries where gender equality is promoted, but not in countries where gender equality is not part of the political agenda. Indeed, Turkish people value equality as much as people in other European countries, but endorse gender equality less strongly (Hanel et al., 2017). Furthermore, equality on an abstract level and gender equality were slightly *negatively* associated in Turkey, but positively in most other European countries.

These differences are not as evident if “meaning” is understood only as abstract conceptualizations of values, which tend to be vague in nature. The concrete actions that people link to values are value instantiations (Hanel et al., 2017). The concept of instantiations originates from cognitive psychology. Instantiating a rule or concept involves applying it to a concrete exemplar (Anderson et al., 1976). ‘Instantiation’ thus refers to a particular realization or instance of an abstraction or to the process of producing such an instance. Instantiation is therefore based on the relationship between general and specific, as in different levels of a conceptual hierarchy. For instance, *football* is an instantiation of the category *sport*, *fork* is an instantiation of *cutlery*, and *pear* is an instantiation of *fruits* (see Hanel et al., 2017, for a more extensive overview).

Maio (2010) suggested that values can be modeled as mental representations on three levels. The first level is the system level, on which values are connected to each other, as in Schwartz’s (1992) model. The second level is the level of specific abstract values (e.g., equality and wealth), which comprise the importance that people attach to the abstract concepts. Finally, the third level is the instantiation level, which includes specific situations, issues, and behaviors relevant to the values.

Similar to instantiations of animals and other categories, research has found that value instantiations can vary in typicality, with important ramifications. For example, Maio et al. (2009) found that contemplation of typical, concrete examples of a value increased subsequent value-related behavior more than did contemplation of atypical examples. That is, the act of thinking about a typical, concrete example of a value led people to be more likely to spontaneously apply the value in a subsequent situation. This finding illustrates the importance of finding typical instantiations over a range of values (perhaps due to their greater familiarity or fit with the ideal or central tendency), which is another aim of Study 1. Based on this finding, Maio (2010) indicated that value instantiations could operate in different ways. More specifically, concrete value instantiations “could (1) affect a strength-related property of the abstract value itself (e.g., value certainty), (2) act as metaphors that we apply to subsequent situations through analogical reasoning, or (3) affect our perceptual readiness to detect the value in subsequent situations” (Maio, 2010, p. 27).

### The Present Studies

In Study 1 we used a qualitative approach to measure (behavior) instantiations of 23 values from Schwartz’s (1992) value model, while comparing them in a systematic way across three countries. To help us examine the value instantiations, participants were asked to report *situations* in which they considered a value to be relevant, including the *people* in this situation and their *actions*. This method was an extension of previous concept-mapping approaches used in the study of attitudes (e.g., Lord et al., 1994), creativity research (Sternberg, 1985), and values (Maio et al., 2009).

We expected that people in different countries would differ in their concrete (behavior) instantiations of values, because we assumed that personal experiences and the socio-cultural environment exert a strong influence at the concrete level (Morris, 2014; Hanel et al., 2017). To test this hypothesis, we collected data from regions of three countries: north-east Brazil, south-west India, and south Wales. These countries differ on various dimensions. In terms of years of schooling, GNP, and life expectancy (United Nations Developmental Programme, 2014), India is the least developed of the three countries, and the United Kingdom is the most developed. Brazil and India are perceived to be much more corrupt than the United Kingdom (Transparency International, 2014), and the homicide rate in Brazil is 25 times higher than in the United Kingdom and almost eight times higher than in India (United Nations Office on Drugs and Crime, 2014). Thus, corruption may be more spontaneously associated with equality as a value and protections against physical violence may be more strongly associated with family security in Brazil than in the United Kingdom. There are also marked differences in climate and natural resources. These differences may well be reflected in differences in value instantiation between the nations. For example, water conservation may be more spontaneously associated with the value of protecting the environment in places where water is scarce (e.g., north-eastern Brazil) than where it is abundant (e.g., most of the United Kingdom). Similarly, waste recycling may be more spontaneously associated with protecting the environment in places where recycling is possible and promoted than where it is not possible and/or not promoted. This difference could emerge even if the absolute or relative importance of the value protecting the environment – a key value relevant to these behaviors – is the same in both types of location. Furthermore, such differences may emerge even if people in both regions recognize the behaviors as potential ways to promote the environment (see Study 2). That is, people in both types of location may recognize that water conservation and recycling protect the environment, but they may simply differ in how strongly they *spontaneously* associate these behaviors with the value in day-to-day life.

With the results of this study in hand, the next substantive issue was whether the (behavior) instantiations that were most frequent in each nation would fit the value as it is conceived in the other nations. That is, even if we focus on the instantiations that appeared only in one nation but not in another, could the instantiations be matched to values (i.e., ‘back-translated’). Study 2 examined the degree to which instantiations could be recognized as belonging to the values from which they originated. For example, would participants recognize recycling as an example of protecting the environment and keeping secrets as an example of loyalty to an equal extent across countries? This step was important because it would reveal the conceptual relevance of the instantiations to the values. In other words, people should be able to recognize the value that a behavioral instantiation promotes, even if the instantiation is atypical for the participant’s own region. This matching would show that the instantiations vary merely in their spontaneous natural activation by values, but not in their conceptual relevance to values. Both studies were approved by the ethics committee of the School of Psychology, Cardiff University. That means that informed consent was obtained by the participants, which included that their participation was voluntarily, they could withdraw at any time without providing a reason, and that the information participants provided would be held anonymously. At the end of each study, participants were fully debriefed. The English versions of the questionnaires used in both studies, along with the two datasets, can be found on https://osf.io/s5vwa/?view_only=6803c67e69af48278640fbcbb2a7b3ea.

## Study 1: Exploring Value Instantiations

This study aimed to find typical value instantiations in Brazil, India, and the United Kingdom and estimate the degree of similarity between them. This aim was achieved using a paradigm that has been used to examine exemplars of natural categories (e.g., Collins and Quillian, 1969), as well as in later research on typicality effects (Fehr and Russell, 1984; Lord et al., 1994; Maio et al., 2009) and on the strength of associations between categories and their members (Fazio et al., 2000). For example, Maio et al. (2009, p. 601) asked participants “to list situations in which they considered equality to be important”. A different approach was chosen by Lord et al. (1994), who asked their participants to complete attitude concept maps on capital punishment and social welfare in order to identify how participants refer to people who are affected by each of those social policies. Specifically, participants were asked to construct a concept map by adding nodes to a central node that stated “capital punishment” or “social welfare,” and the added nodes were generated in response to questions asking “what,” “where,” “when,” “who,” “why,” and “how”.

Following those examples, in Study 1 participants were asked to list situations in which they considered a value to be important and to include people and their actions. These responses were then used to create a conceptual map representing values and value instantiations for each country. These maps were similar to those created by Lord et al. (1994, p. 661), except that our method maps values, rather than natural concepts (see the 23 figures in the Supplementary Materials for such ‘value maps,’ one for each of the 23 value investigated in this study).

### Method

#### Participants in Brazil

Participants were 189 mostly postgraduate students from João Pessoa, a coastal city from north-east in Brazil. Participants were not compensated. The average socioeconomic status (SES; Sharma et al., 2012) of 18.50 indicates that the average participant was part of the Brazilian upper-middle class (see **Table 3** for details).

#### Participants in India

Participants were 214 undergraduate and graduate students from Dharwad, south-west India. Participants were not compensated. The mean SES was 20.78, indicating that the average participant was part of the Indian upper-middle class (see **Table 3** for details).

#### Participants in the United Kingdom

Of the 227 participants in the United Kingdom, 122 were psychology undergraduate students, and 105 were other members of Cardiff University (students or staff). The students received course credits in exchange for their participation, and other university members could add their name to a raffle of three cash prizes of $30, $20, and $10. The participants’ SES was similar to the SES of participants in the two other countries (**Table 1**).

**Table 1:** Demographic details of the two samples.

	Age	% Women	SES
Brazil	25 (7.98)	67.00	18.50 (4.86)
India	22.41 (5.15)	66.40	20.78 (5.15)
United Kingdom	22.17 (7.94)	79.90	18.60 (5.74)


**Standard deviations (SD) are in brackets, where applicable. The SES scale ranges from 3 to 29.**

#### Design

The design was qualitative and entailed the use of open questions.

#### Materials

We examined 23 out of the 56 values of Schwartz (1992) value model (see **Table 2**). The values were selected according to their perceived relevance for explaining cross-cultural differences. That is, we expected the instantiations for the chosen values to be more varied than for some other, non-chosen values. From most value types, two values were selected. The exception was the value type universalism, for which seven values were selected, with an eye to potential future research. To measure socioeconomic status, Kuppuswamy’s Socioeconomic Scale (Sharma et al., 2012) was used; it consists of three items, assessing education, occupation, and family income per month. Responses were summed up to one score. To adjust the income classes, the most recent available official income distribution from all countries was used. The questionnaire was translated to Portuguese from the original English version for the Brazilian sample by an experienced translator. The translation was double-checked by others who are fluent in both languages. The questionnaire was in English for the Indian and British samples.

**Table 2:** Length of average responses for each value and number of participants.

Value	Brazil Ø	*N* (BR)	India Ø	*N* (IND)	United Kingdom Ø	*N* (United Kingdom)
Protecting the environment (UN)	644	34	238	33	316	35
Wisdom (UN)	511	32	211	25	317	37
Unity with nature (UN)	554	32	185	28	252	31
World of beauty (UN)	607	31	264	28	295	35
Social justice (UN)	571	30	234	31	250	27
Broad-mindedness (UN)	517	33	220	35	309	29
Equality (UN)	574	25	278	39	268	25
Freedom (SD)	474	32	240	38	245	37
Creativity (SD)	532	35	225	34	322	44
A varied life (ST)	551	28	216	27	232	37
Daring (ST)	566	26	217	37	301	23
Pleasure (HE)	599	36	189	34	265	37
Success (AC)	602	35	227	40	353	41
Ambition (AC)	511	34	195	35	323	34
Wealth (PO)	557	27	261	33	307	27
Social power (PO)	680	25	318	31	332	29
Family security (SE)	477	30	219	31	281	27
Respect for tradition (TR)	522	33	204	37	275	39
Self-discipline (CO)	489	29	239	36	438	35
Obedience (CO)	501	33	329	37	370	33
Helpfulness (BE)	531	36	215	28	330	31
Loyalty (BE)	534	36	247	33	343	34
Honesty (BE)	574	30	273	36	320	36


**Ø, average number of characters of responses including spaces; N, number of participants. The value types are in brackets. UN, universalism; SD, self-direction; ST, Stimulation; HE, hedonism; AC, achievement; PO, power; SE, security; TR, tradition; CO, conformity; BE, benevolence.**

#### Procedure

Participants were asked to list typical situations in which they considered each value to be important. Furthermore, they were asked to include a “short description of the people in the situation and what they do.” The instructions provided two examples that pertained to two values not included in our measures or in Schwartz’s value model: “For example, the value ‘enjoyment’ could be relevant during leisure time. Relevant people in the situation can be friends and the family. They could spend time together at the beach or playing games at home.”

Participants were asked to list at least two to three situations, people, and actions for each value, up to a total of seven. To reduce the risk of fatigue, each participant responded to four out of the 23 values (see **Table 2** for the sample size for each value), resulting in approximately 30 to 40 participants per value. Subsequently, participants completed socio-demographic items. Brazilian and British participants completed the survey online, while Indian participants used a pen-and-paper version.

### Data Analysis

The data were analyzed with the open access program Iramuteq, which is built on R and Python and designed for content and frequency analyzes (version 0.6 alpha 3; Ratinaud, 2009). The data were analyzed separately for each value and country. For all analyses, very similar words (e.g., people and person) as well as different verb forms (e.g., advice, advises, and advised) were treated as equivalent. Additionally, we grouped together certain words that seemed very similar (e.g., parents, dad/father, and mother/mum), but in general this was generally avoided because participants may have used the words in different ways even if they seemed similar to us. Furthermore, only nouns, verbs, and adjectives were analyzed.

To analyze the data, we conducted an explicit and implicit content analysis, because both the length (see **Table 2**) and the comprehensibility of the responses differed across countries. We struggled to interpret some of the responses, especially those made by Indian respondents. Therefore, an explicit content analysis seemed to be appropriate, because the meaning of a single word is usually easier to understand than the meaning of a sentence. Explicit content analysis “locates what words or phrases are explicitly in the text, or the frequency with which they occur” (Carley, 1990, p. 2). This analysis is straightforward and easy to reproduce, but can miss out the meaning. In contrast, an implicit content analysis aims to detect the meaning of what is said (Carley, 1990). However, because the responses of British and Indian participants were much shorter than those of Brazilian participants, an implicit content analysis was difficult to produce. The British and Indian responses often consisted of only one word (e.g., “recycling” for a situation in which protecting the environment is relevant for British participants). This problem was identified after carefully reading all responses.

Next, we conducted an automated explicit content analysis with Iramuteq by counting the frequencies. We then re-read all responses which contained words that were mentioned at least by 20% of the participants to get a better understanding of the context in which the word was mentioned (i.e., implicit content analysis). The cut-off point was set to identify *prospective* typical instantiations, and we noted which behaviors were mentioned 10 times or more by at least five participants in one country. This threshold was selected because it enabled us to consider between 5 and 10 instantiations as candidates in each country. This procedure was not intended to definitively identify the typical instantiations, but to identify a range of instantiations that are potentially typical exemplars. In the concept mapping approach (Lord et al., 1994), instantiations that are mentioned very rarely or not at all are regarded as unlikely to be core aspects of the concept, whereas frequent instantiations are seen as plausible candidates. These were then compared between the nations and considered for future study.

Finally, we re-read all responses to ensure that we had not missed any meaning or theme which was not flagged up in the frequency analysis conducted with Iramuteq, which was rarely the case. Below we report and discuss instantiations that were mentioned by at least 50% of the participants per value in each country and in the Supplementary Materials we also list 5 to 10 other instantiations per value and country that were mentioned by around 20% of the participants.

Because hardly any negations (e.g., “recycling is not relevant”) were used by Brazilian and British participants, the absolute frequencies of specific words and their connections are meaningful. Indian participants used more negations, which itself is an interesting finding, reflecting the fact that they seemed to focus more on what a value does not mean. However, we do not consider this to be an issue for the analysis, because such occurrences were still rare and they appear to have been used to express the same points as if the affirmative had been used. For example, one instantiation for the value helpfulness, “people do not come forward and rescue the victim, though they can,” was reported as an example of action antithetical to helpfulness, and was therefore judged to be equal to the hypothetical positive version (“rescue the victim”). The Brazilian instantiations were first identified by a native speaker and then translated by an experienced translator (Portuguese native speaker), who ensured that the meaning was correctly translated.

Because the three different facets of a given response – “situation,” “people in the situation,” and “what are they doing” – were all part of the instantiation, they were analyzed together. Furthermore, family, friends, and people or person were mentioned for most values at least 10 times as the “relevant people in the situation.” The value itself was also very frequently mentioned. Therefore, these responses are not informative and are not discussed further. The frequencies of these words are nevertheless listed in the Supplementary Materials.

All authors contributed to the data analysis and interpretation: The Brazilian data were analyzed and interpreted by the Brazilian authors of this paper and the authors based in the United Kingdom. The Indian data were analyzed and interpreted by the Indian authors of this paper and the authors based in the United Kingdom. The British data were analyzed and interpreted by the authors based in the United Kingdom.

### Results and Discussion

The responses of the Brazilian participants for each value were on average nearly twice as long as the responses from Indian and British participants (see **Table 2**). The number of words mentioned at least 10 times barely differed between the Brazilian and the British sample. The number of words mentioned by at least 20% of the sample was lower in the Indian sample, resulting in fewer potentially typical instantiations in this sample.

Detailed analyses for each value can be found in the Supplementary Materials. There we list how often the most common instantiations of each country were mentioned and by how many participants. To address the question of whether value instantiations are more influenced by culture than values on an abstract level, we counted the number of instantiations that were mentioned by at least 50% of the participants in each country. If culture shapes how values are instantiated, people in each country should have a common understanding of values. We used 50% as an admittedly arbitrary threshold to define common understanding because of our relatively small sample sizes for each value (around 35 participants responded to each value in each country). This approach also allowed us to focus on larger effects, thus reducing the probability of a Type-I error.

As can be seen in **Table 3**, for 11 out of the 23 values, 7 instantiations mentioned by at least 50% of the participants were found in Brazil, and another 7 in the United Kingdom. For example, 50% (18 out of 36) Brazilian participants mentioned spending time with the family as an instantiation for the value ‘pleasure’ and 58% (21 out of 36) British participants considered relationships as an instantiation of ‘honesty’ (mainly in the sense that honesty is important in a relationship). In India, no instantiation was mentioned by at least 50% of the participants.

**Table 3:** Instantiations mentioned by ≥50% of the participants in each country.

Value	Brazil	United Kingdom
Protecting the environment (UN)	Putting rubbish in the bin (21/34)	
Equality (UN)	Equal opportunities for all (14/25)	
Creativity (SD)		Making or creating art (25/44)
A varied life (ST)		Doing varied activities at work (20/37)
Pleasure (HE)	Spending time with friends (21/36) and family (18/36)	Spending time with friends (25/37) and family (20/37)
Ambition (AC)		Working or work place (28/34)
Family security (SE)	Supporting parents (16/30)	
Self-discipline (CO)		Work (18/35)
Obedience (CO)	Obey parents (21/33)	
Helpfulness (BE)	Work (18/36)	
Honesty (BE)		Relationship (21/36)


**Number in brackets indicated how many respondents mentioned a particular instantiation out of the total number of respondents.**

In a next step, we computed the number of instantiations mentioned by at least 50% more participants in one nation than in another country. However, because the majority of all instantiations in all countries were mentioned by less than 50% of the participants, only two instantiations revealed large differences: 62% of the Brazilian participants considered throwing garbage into a bin as typical for ‘protecting the environment,’ whereas only 3% of the Indian participants did so. Also, 57% of the British participants mentioned art as a typical instantiation of ‘creativity,’ whereas only 6% of the Indian participants did so.

Finally, we looked for similar instantiations in different values across all samples. In the descriptive analyses above and the Supplementary Materials, it is easy to discern a number of instances in which participants in one nation used the same example for a different value than was used in another nation. To illustrate this diversity with only the relatively frequent examples, we list here four words which were mentioned at least 10 times for different value types. *New* was relevant for ambition (self-enhancement) and daring, varied life, creativity, broad-mindedness (openness and self-transcendence); *support* was relevant for family security (conservation) as well as loyalty (self-transcendence); and *work* was relevant for success and ambition (self-enhancement) and creativity (openness).

Some other examples of overlap were found in the Brazilian sample. In particular, typical instantiations of wealth in this sample often focused on a good family life, thereby overlapping wealth with family security. In addition, Brazilian participants understood social power more as social responsibility.

While Study 1 focused on the comparison of Brazil, India, and the United Kingdom, in Study 2 we focused only on Brazil and the United Kingdom. This was done because the quality of the responses of the Indian participants was overall low. This finding was surprising because some of the authors of this paper have successfully conducted multiple quantitative studies with student samples from the same departments of the Indian university with overall reliable results. This suggests that the English proficiency of most students might have been adequate for quantitative research, but not for qualitative research.

## Study 2: Matching Instantiations to Values

In Study 1 we found that, although few instantiations were mentioned by more than 50% of the participants in each country, some were mentioned more frequently by participants in one country than in one or both of the other countries. Of importance, these instantiations were produced spontaneously as examples of the values. If they are valid examples of the values, then these spontaneously produced exemplars should be correctly regarded as value instantiations; that is, when presented with an exemplar, people should be able to identify the value that elicited it. More importantly, we wanted to establish whether the examples would be seen as valid even in a country in which they had not been frequently generated. Should this be the case, it would indicate that the nations differ primarily with respect to the nature of the spontaneously produced examples, but not with respect to whether the examples are regarded as valid and therefore defining of the value. In other words, such a finding would show that the concrete examples of values that spontaneously come to mind in the mental representations differ between countries, but that the abstract meaning of the values is similar enough that even examples that do not spontaneously come to mind are seen as valid instances of a given value. The aim of Study 2 was therefore to test whether instantiations can be reliably matched to the values from which they were derived.

### Method

#### Participants in Brazil

In Brazil, 427 under- and postgraduate students (mainly in psychology), from João Pessoa participated (*M*_age_ = 23.42, *SD*_age_ = 6.96, 64.60% women). They were not compensated.

#### Participants in the United Kingdom

British participants were 250 psychology undergraduate students (*M*_age_ = 19.32, *SD*_age_ = 2.25, 89.00% women) from Cardiff University. They received course credits in exchange for their participation. Prior to data analysis, 42 non-British participants were excluded, to be consistent with the homogeneous Brazilian sample.

#### Material and Procedure

One-hundred thirty-eight instantiations were chosen to be matched to values, six for each of the 23 values. The instantiations were chosen mainly based on the results of Study 1, but also for exploratory purposes. The instantiations used were *a priori* categorized as either typical (i.e., mentioned frequently) in the United Kingdom, typical in both countries, typical in Brazil, or not typical in either country. The latter group were instantiations that we generated for exploratory purposes, based on their perceived relevance to the present research and also based on previous studies. They were used when there were fewer than six instantiations that seemed suitable in the first three categories. For example, Maio et al. (2009) found that discrimination against left-handed people is an atypical (albeit highly unacceptable) instantiation for equality for British participants. Thus, we expected that this atypical example would be recognized as an instantiation of equality by British participants and also, presumably, by Brazilian participants.

The instantiations selected from Study 1 were chosen based on the frequency with which they were mentioned in each country, while balancing the instantiations that were mentioned in both countries with those mentioned in only one country but not the other. Typical instantiations for protecting the environment, for example, were (1) “Putting certain rubbish in recycle bins rather than general waste,” (2) “Making sure the lights are off,” (3) “Walk instead of using car for short distances,” (4) “Throwing garbage in the bin,” (5) “Saving water,” and (6) “Installing heat insulation in the house.” The first three instantiations were considered as more typical by British than Brazilian participants (Study 1, see Supplementary Materials), whereas the fifth instantiation was considered more typical by Brazilian participants. The fourth instantiation was frequently mentioned by participants in both countries, and the sixth instantiation was added for exploratory purposes. Given the differences in climate between João Pessoa and Cardiff, we expected this last instantiation to be more reliably matched to the value ‘protecting the environment’ by British than by Brazilian participants. A list of all 138 (137 in the United Kingdom) instantiations can be found in the Supplementary Table S70, including the values they were derived from and whether they were mentioned by participants in both countries, just one country, or were added by us.^1^

The instruction to the participants was: “Your task in this study is simple: You will be given a specific situation and you are asked to choose the most suitable value in this situation.” This was followed by an example: “Leisure time is promoted most by valuing …”. This stem was followed by six values (in the current example: success, equality, ambition, wisdom, enjoyment, and respect for tradition), and a seventh “don’t know” option. Our example then stated a possible solution: “A possible answer is the value enjoyment: Leisure time is more related to the value enjoyment than to any other value in this set.” For this example, we intentionally selected a value that is not part of Schwartz’s value model. Both the ordinal position of the ‘correct value’^2^ among the response alternatives and the five alternative values were chosen randomly. The five alternative values were a subset of the 23 values from Schwartz’s 56 values listed in Study 1. Within the six instantiations of one value, both the order and the alternatives were kept constant. The five alternative values were kept constant across both countries. All participants then completed further scales, unrelated to the present study. On average, each instantiation was matched with values by 71 Brazilian and 41 British respondents.

Brazilian participants completed a paper version of the survey in classroom settings of 10 to 40 people. British participants completed the survey online. To reduce fatigue, each participant completed only one-sixth of the items, with each participant responding to one instantiation per value.

### Results and Discussion

To perform the principal analyses, we first counted how often each value was identified as being promoted by an instantiation, separately for each country (see Supplementary Table S70). Next, we compared for each instantiation and each country whether the most frequently chosen response option (whether this was a value or don’t know) was chosen significantly more often than the second-most commonly chosen option, using χ^2^-tests. This is a conservative approach, which partly takes the research design (multiple choice) and the influence of the response alternatives into account. For example, if the ‘correct’ value was chosen by 20 out of 40 British participants, another value by 12, and a third by 8 participants, we would not count it as correctly matched, because the difference between 20 and 12 is not significant, χ^2^ = 2.00, *p* = 0.16.

Overall, in both countries, most instantiations were correctly matched with the value from which they were derived (see Supplementary Table S70). Of the 138 (137 in the United Kingdom) instantiations, 94 were correctly matched by the Brazilian participants and 110 by the British participants. This difference (94 vs. 110) did not reach statistical significance, χ^2^(1) = 0.63, *p* = 0.43. Indeed, the similarities were much larger: both British and Brazilian participants were significantly more likely to choose the same, ‘correct’ value 86 out of 137 times. That is, they chose the same value significantly more often than any other value (or the ‘don’t know’ response). For another 12 instantiations, no value was chosen significantly more often than the second most frequent value in both countries.

For example, the instantiation “Putting certain rubbish in recycle bins rather than general waste” was correctly identified in both countries by the majority of participants as being promoted by the value protecting the environment (54 out of 67 Brazilian participants did so and 42 out of 43 British participants). In the Brazilian sample, the number of participants who chose protecting the environment differed significantly from the number of participants who chose the second-most frequently chosen value, helpfulness (54 vs. 9, χ^2^ = 32.14, *p* < 0.001). Overall, Brazilian participants correctly matched five out of the six instantiations for protecting the environment, and British participants correctly matched all six instantiations to protecting the environment. As can be seen in **Table 4**, participants from both countries were approximately equally likely to match instantiations that had been mentioned in both countries (columns 3 and 8), mentioned more frequently in Brazil, and also the exploratory instantiations. Brazilian participants had somewhat more difficulty in matching British instantiations, compared to their British counterparts (34 vs. 45, respectively), although this difference did not reach statistical significance, χ^2^ = 1.53, *p* = 0.22.

**Table 4:** Frequencies of correctly matched instantiations for all values combined and depending on the origin of the instantiation.

	Brazilian responses					British responses				
		
	United Kingdom	All	Brazil	None	Sum	United Kingdom	All	Brazil	None	Sum
Unity with nature (UN)	2/2		3/3	0/1	5	2/2		3/3	1/1	6
Wisdom (UN)	2/2	1/1	2/3		5	2/2	1/1	1/3		4
World of beauty (UN)	2/2		0/3	1/1	3	2/2		0/3	1/1	3
Social justice (UN)	2/2		3/3	1/1	6	2/2		3/3	1/1	6
Broad-mindedness (UN)	0/1		1/3	2/2	3	1/1		1/3	1/2	3
Protecting the environment (UN)	3/3	1/1	1/1	0/1	5	3/3	1/1	1/1	1/1	6
Equality (UN)	1/3		2/2	0/1	3	3/3		2/2	0/1	5
Freedom (SD)	2/2		1/3	1/1	4	2/2		3/3	1/1	6
Creativity (SD)	2/2	1/2	0/2		3	2/2	2/2	1/2		5
A varied life (ST)	0/2	0/1	0/3		0	2/2	1/1	1/3		4
Daring (ST)	2/2		2/2	1/2	5	2/2		2/2	2/2	6
Pleasure (HE)	2/2	2/2	1/2		5	2/2	1/2	2/2		5
Success (AC)	2/3		3/3		5	3/3		2/3		5
Ambition (AC)	2/3		2/3		4	3/3		2/3		5
Wealth (PO)	1/2	0/1	0/2	0/1	1	1/2	0/1	0/2	1/1	2
Social power (PO)	2/2		3/3	1/1	6	2/2		1/3	1/1	4
Family security (SE)	1/3		1/3		2	3/3		2/3		5
Respect for tradition (TR)		1/2	3/3	0/1	4		2/2	2/3	0/1	4
Self-discipline (CO)	2/3	1/1	1/1	1/1	5	3/3	1/1	1/1	0/1	5
Obedience (CO)		2/3	2/3		4		3/3	2/3		5
Helpfulness (BE)	0/1	2/2	2/2	0/1	4	1/1	2/2	2/2	0/1	5
Loyalty (BE)	3/3	1/1	2/2		6	3/3	1/1	2/2		6
Honesty (BE)	1/1	1/1	2/2	2/2	6	1/1	1/1	1/1	2/2	5
Sum (maximum 138)	34/46	13/18	37/57	10/17	94/138	45/46	16/18	37/56	12/17	110/137


**Value type is in brackets. UN, universalism; SD, self-direction; ST, stimulation; HE, hedonism; AC, achievement; PO, power; SE, security; TR, tradition; CO, conformity; BE, benevolence. United Kingdom, absolute frequency of typical British instantiations (and how often these were correctly matched; All, frequency of instantiations typical in both countries; Brazil, frequency of typical Brazilian instantiations; None, instantiations that were neither typical in Brazil nor the United Kingdom (i.e., those generated by me).**

In a final step, we computed how often differences occurred based on the taxonomy proposed in Study 1, while taking the unequal sample sizes into account. We compared all values that were mentioned by at least half the participants in one country with the percentage of participants choosing the same value in the other country. We focused on differences where one option was chosen by at least 50% more of the participants in one group than the other. Fifty percent was chosen as a cut-off value because it allowed us to focus on larger effects while reducing the probability of a Type-I error. For example, if 20% of the Brazilian participants reported that they thought that a specific instantiation is best promoted by wealth, at least 70% of the British participants (a difference of 50%) needed to choose wealth before we would call it a difference. This 50% cut-off value also aligns approximately with a *p*-value of 0.001 of a χ^2^-test, which in our view adequately controls for multiple-comparisons.

Differences were found for five instantiations (see Supplementary Table S70): ‘Traveling’ was considered to be best promoted by the value of ‘pleasure’ in the Brazilian sample and by ‘freedom’ in the British sample (84% of the Brazilian participants chose pleasure vs. 25% of the British participants and 13% of the Brazilian participants chose freedom vs. 75% in the British sample; see Supplementary Table S70). ‘Maintaining a good work life balance’ was considered to be promoted by ‘success’ in the Brazilian sample, but not in the British sample (73% vs. 12%), whereas British participants correctly matched this instantiation to ‘a varied life’ more often than Brazilian participants did (79% vs. 6%). ‘Being able to buy organic food’ was considered to be promoted by ‘wealth’ by British participants, but not by their Brazilian counterparts (61% vs. 6%). ‘Living your own life and not following the crowd’ was considered to be promoted by ‘self-discipline’ by Brazilian participants, but not by their British counterparts (84% vs. 13%), whereas the reverse applied for the value of ‘freedom’ (1% vs. 83%). This is an interesting finding because freedom and self-discipline are thought to be motivationally incongruent (Schwartz, 1992), but nevertheless appear to be related in the Brazilian respondents’ views of their social relationships. Finally, ‘customer service’ was thought to be promoted by ‘social justice’ by Brazilian participants (61% vs. 7%), whose country is one where cultural issues of corruption are relevant, but was correctly matched to ‘helpfulness’ by British participants (83% vs. 21%).

## General Discussion

The aim of this research was to explore whether value instantiations vary across countries, despite there being similarities in values at an abstract level (Fischer and Schwartz, 2011). We first discuss the implications and limitations of Study 1, before turning to Study 2.

### Implications of Study 1

In Study 1, we explored concrete examples (i.e., instantiations) associated with values across 23 values and 3 countries. This design enabled us to test the hypothesis that on a concrete level values differ between countries. However, only a few differences were found. There was large individual variability in the responses within countries, which made it difficult to detect differences between countries. This can be explained in terms of the ‘value as truism’ hypothesis (Maio and Olson, 1998). People usually do not think about their values or discuss them with others in order to arrive at a shared understanding of the meaning of values. If, for example, students were to discuss whether freedom is important, they would presumably develop a more shared understanding of this value.

This variation in responses within and between countries has further implications relating to possible misunderstandings both within and perhaps especially between countries. Take the value of ‘protecting the environment,’ for example. If a Brazilian, an Indian, and a British person were to talk about the importance of protecting the environment, they might easily talk past each other, because it is quite likely that they would have somewhat different understandings of it. For example, the Briton might conceive of protecting the environment as entailing the reduction of carbon emissions, whereas the Brazilian and Indian individuals might be thinking of putting rubbish into a bin. This implication is consistent with research in law and political sciences. There it has been argued that “human dignity” is understood differently both across jurisdictions and also (over time) within jurisdictions (McCrudden, 2008), resulting in intergovernmental and intergenerational misunderstandings, as governments treat their citizens based on their own interpretation of human dignity.

One conclusion from Study 1 is therefore that debate and discussion would be more constructive, and behavioral change interventions more effective if they linked the abstract values being considered to more concrete exemplars. Linking actions to abstract values carries a prescriptive, motivational impetus, which can predict behavior independently of attitudes, norms, and other constructs often used to predict behavior (Schwartz and Tessler, 1972; Maio and Olson, 2000). By making the connections of values to an action explicit, people can reason through their relevant attitudes and intentions to achieve better fit with their values. Such an approach could be used to support intervention programs, which have to deal with the fact that several behaviors are closely linked to values. For example, protecting the environment is usually considered to be an important value (Schwartz and Bardi, 2001), but can be linked to a variety of behaviors. Nonetheless, some of these behaviors are more damaging to the environment than others. For example, it may be more beneficial to alert participants to the fact that avoiding short distance flights or installing good heat insulation are effective ways of protecting the environment, rather than simply reminding people that environmental protection is important. Most people already agree that this value is important, and they might imagine that engaging in less impactful behaviors (e.g., recycling) demonstrates their support for the value. Highlighting important behaviors about the value should help to change their perceived typicality with respect to the value and the motivational impetus attached to these actions.

The only two exceptions where we found large differences between countries pertained to the values of ‘protecting the environment’ and ‘creativity.’ Specifically, Brazilians considered throwing garbage into a bin to be a typical instantiation of protecting the environment, whereas Indian participants did not. This finding is in line with our casual observation of the regions in Brazil and India from where the data were collected: the streets and roadside ditches in Brazil were much cleaner than those in India. Indeed, previous research found that 98% of Brazilian college students felt uncomfortable or very uncomfortable when seeing garbage all over the ground (Profice and Edington, 2014) – a common sight in many places in India. Further, more than half of the British participants mentioned ‘art’ as an instantiation of ‘creativity,’ whereas hardly any Indian participants did so. This indicates that the so-called art bias, “the misunderstanding of creativity that equates it with artistic talent” (Runco, 2007, p. 384), could be a Western phenomenon (see Hanel et al., unpublished for follow-up studies).

Other meaningful differences across countries were related to contextual differences. For example, a typical instantiation of ‘success’ for Brazilian participants was ‘passing an entrance test,’ which is highly competitive in that country, but promises a prestigious job with a permanent contract. Another example is that Indian participants mentioned castes or caste-ism, mainly in relation to ‘equality,’ but also in connection with other values. This refers to a social system that does not exist in Brazil and the United Kingdom, although prejudice based on social class is somewhat similar. These examples show that the examples provided by participants depended to some degree on the social and physical environment in which they live.

Another aim of Study 1 was to identify instantiations that are more frequent in one country than another, to select these for further confirmatory studies. There were a number of findings suggesting that the presence or absence of instantiations in participants’ responses to the open-ended questions used in this research are not suitable to serve as the *sole* criteria for selecting typical instantiations. For example, the presence of the same examples in relation to different values is a complicating factor. There were many instances of the same context being referenced for different values. In some instances, the same example was used for motivationally similar values, but countries varied with respect to which value generated the example (e.g., ‘meeting new people’ used for ‘broadmindedness’ in the United Kingdom, but used for ‘a varied life’ in Brazil). This pattern suggests that small shifts in understanding the meaning of the values may affect which examples are given.

Our approach can be generalized to other psychological constructs, such as goals (Grouzet et al., 2005) and personality traits (McCrae and Costa, 2003; Ashton et al., 2004). The importance of instantiations is especially relevant to measures that require participants to respond to single-word items, such as the markers of the Big-5 traits (Goldberg, 1992; Saucier, 1994) because they are not embedded in a context or defined, thus increasing the likelihood that the adjectives are differently instantiated. When completing such items, participants indicate how well adjectives such as ‘creative,’ ‘philosophical,’ or ‘warm’ describe themselves. However, participants across different groups might instantiate these adjectives differently. Future research could therefore investigate whether differences in how these adjectives are instantiated can account for potential failures to replicate the five-factor model of personality in some countries (McCrae et al., 2005; Gurven et al., 2013). For example, if ‘creative’ is differently instantiated in different countries, the relations with other items of the same factor, and thus the factor loadings, are likely to differ. Further, as outlined above for values, knowing the trait instantiations might help to predict the trait-behavior link.

### Limitations of Study 1

An important limitation of Study 1 is that it is likely that participants’ open-ended responses occasionally miss typical instantiations that they take for granted and, therefore, may neglect to mention. For instance, prior research has identified Blacks and women as two groups that are often used to instantiate the value of (lack of) equality in the United Kingdom (Maio et al., 2009). However, these groups were mentioned in Brazil, but *not* in the United Kingdom. Conversational norms apply to the information that participants might choose to identify, and one important norm is not offering information already mutually understood (one of the Gricean maxims; Grice, 1975). This might sometimes cause people to neglect to report common instantiations that are not salient. Another possibility is that participants’ responses are somewhat egocentric. That is, treating students or job applicants equally is something that would directly affect the British participants, whereas equal treatment of Black people does not (bearing in mind that most of the British participants were Caucasian). Although these observations are speculative, they show that open-ended measures of concept mapping, as used here and in past research, are likely to be unreliable as *sole* measures of the typicality of an exemplar.

Another issue is that although some of the observed differences in instantiations are clearly explicable in terms of contextual factors, others are more difficult to explain. Examples of readily explicable differences in instantiations include references to caste-ism in the Indian sample and the association of ‘electric fences’ with ‘family security’ among Brazilian participants, given that caste-ism does not exist in Brazil and the United Kingdom, and both India and the United Kingdom are safer than Brazil (Office for National Statistics, 2014). A difference that is more difficult to explain is in the use of ‘saving water’ for ‘protecting the environment.’ Although it seems obvious why saving water was mentioned more often in the relatively dry north-east of Brazil than in rainy Wales, it is less clear why saving water was barely mentioned by the Indian participants. Water conservation is an aspect of daily life in the region where this research was conducted (Karnataka), making it highly relevant to the residents. However, they did not spontaneously think of this behavior in relation to environmental protection. This may be a case where an instantiation is taken for granted, making it less salient to respondents (Gricean maxims; Grice, 1975). Alternatively, it may be the case that water conservation is seen as a basic necessity rather than a way to protect the environment. As a result, Indian participants may have perceived water shortage as a personal challenge rather than a challenge to the environment.

A further limitation pertains to the samples used. Because most participants were students in specific regions of each nation, generalizing to the population of each country should be done with caution (cf. Hanel and Vione, 2016). For example, Brazilians mentioned passing entrance exams for prestigious jobs as an instantiation. However, it is less likely that people who are close to retirement would also regard this as an instantiation for success. In other words, the instantiations seem also to be shaped by respondents’ age and educational level. Further, although the instantiations are in general not in line with typical gender stereotypes, similar limitations may pertain to the large proportion of female participants in all samples.

Finally, the answers in the Indian data were more heterogeneous (i.e., fewer typical instantiations) and were grammatically challenging to analyze, because of many grammatical errors. Most of the Indian participants did not have English as a first language, although English was the language of instruction both in school and at university. As a result, English proficiency varied substantially between participants. Another possible explanation for the difficulties we had in parsing the Indian responses is that Indian participants used a line of thought that was too unique for us to follow. This is sometimes a problem in anthropological research (Barley, 1986). We sought to minimize the extent of this problem by working closely with our Indian collaborator. Despite the difficulties in interpreting the Indian data, we did not exclude it because excluding conditions is perceived to be bad practice (Simmons et al., 2011) and it might be an useful for other researchers who seek to do qualitative research in India.

We aimed to get an overview of typical instantiations across 23 values and 3 countries. However, because of large within- and surprisingly small between-country variabilities in combination with relatively small samples sizes of around 30 participants in each country, cross-cultural comparisons were difficult. Thus, future research might want to measure instantiations in larger samples to detect potential (small) effects of group membership (e.g., culture) on how values are instantiated. Larger sample sizes would also allow one to test for moderators. For example, do left- and right-wingers instantiate conservation and openness values differently? A further possibility is to ask participants to describe three situations in the past in which they applied the value themselves or have seen applications of the value.

### Implications of Study 2

The aim of Study 2 was to test the extent to which the instantiations obtained in Study 1 would be recognized as being promoted by the specific value that had elicited them. Most instantiations were correctly matched in both the United Kingdom and Brazil, indicating a relatively similar understanding of which instantiations are related to which values. Interestingly, participants were often able to correctly match instantiations that their compatriots had not mentioned in the free recall procedure used in Study 1. For example, although British participants in Study 1 did not mention ‘saving water’ as often as their Brazilian counterparts did when asked to identify behaviors that ‘protect the environment,’ participants in both countries were able to correctly match saving water to protection of the environment. Thus, the findings of Studies 1 and 2 converge with evidence from cognitive psychology indicating that most people are able to recognize instances of a category, even when the instances are atypical; for example, people can label an ostrich or a penguin as members of the bird category, even though these birds are seldom the first examples that come to mind when participants were asked to name birds (e.g., Mervis and Rosch, 1981). Hence, the instantiations that have been correctly matched can be regarded as valid instantiations, but are potentially atypical when they were not spontaneously generated in Study 1.

### Limitations of Study 2

An obvious limitation of Study 2 is the use of fixed response alternatives, i.e., the six values that could be selected as best promoting a specific instantiation. Although five of the six values were chosen randomly (with the remaining value being the one related to the instantiation), they were the same across participants and countries for all six instantiations of each value. Consequently, although we can compare findings between participants and regions, we cannot do so between value instantiations and values. If the five alternative values had been selected out a broader range of values (e.g., Schwartz’s, 1992, 57 values), a much larger sample would have been required to achieve adequate power. In other words, conclusions such as “instantiation A was more reliably matched to value *X* than instantiation B to value *Y*” cannot be drawn, because these comparisons also depend on the response alternatives. On the other hand, between-country conclusions such as “instantiation A was more often ‘correctly’ matched to value *X* in Brazil than the United Kingdom” are justified, given that participants in both countries were given the same response alternatives. However, between-country comparisons may also be moderated by the choice of response alternatives. It might be the case that the nature of the differences between countries depends on which response options are offered. Nonetheless, given that these options were chosen randomly, there is no reason to suspect any *systematic* effect of the options on the between-country comparisons.

### Future Research

Our results suggest that some behaviors are more closely associated with some values than other behaviors. Thus, an unanswered question is whether the value-behavior link is moderated by the typicality of an instantiation (behavior). This issue is theoretically important because it points to different ways in which typicality might affect the role of values in behavior. This consideration is based on attitude representation theory (ART; Lord and Lepper, 1999). The ART postulates, based on previous findings of the authors (e.g., Lord et al., 1984), that attitude-behavior consistency is moderated by typicality. As argued above, both personal experiences and social-contextual factors influence the extent to which a behavior is a prominent instantiation of values. This, in turn, leads to the activation of one or more values that influence which behavior is chosen in a specific situation (cf. the representation postulate of the ART). Thus, not only the attitude-behavior link should be moderated by typicality, but also the value-behavior link: If an instantiation (here: behavior or behavioral intention) is more closely linked to a value, the two are more strongly associated. It is important to know whether typicality matters, because it allows us to better predict when values are correlated with behavior. For example, one might expect protecting the environment predicts saving water in the United Kingdom but not in Brazil. In conclusion, we hope that our findings allow researchers to develop more specific hypotheses in which context and for which sample type a value predicts a behavior.

## Conclusion

Overall, Study 1 revealed that most examples that are spontaneously attached to values vary in how much they are shaped by context. In most cases, within-country variability outweighed between-country differences. Nevertheless, many of the instances for which between-country differences were found could be linked to contextual factors. In Study 2, we found that most instantiations that had been spontaneously produced by participants in another country could reliably be matched to the values that they exemplified. Taken together, our results further challenge “the prevailing conception of culture as shared meaning system” (Schwartz, 2014, p. 5), as long as culture is equated with country or nation: the within-country variability outweighs the between-country variability, similar to values on an abstract level (Fischer and Schwartz, 2011). In other words, people endorse the same values to a similar extent across countries and also instantiate them similarly. We hope this research helps to lay a foundation for future research examining these differences and their implications for intercultural understanding and communication.

## Author’s Note

Both studies are described in the Ph.D.-thesis of the first author (Hanel, 2016).

## Author Contributions

GM and AM: conceptualization. PH, GM, and AM: study design. GM, VG, SK, and AM: funding acquisition. PH, GM, AS, KV, GdHC, VG, AP, SK, and AM: data analysis and provided critical revision. PH and AS: visualizations. PH, GM, and AM: wrote the original draft.

## Conflict of Interest Statement

The authors declare that the research was conducted in the absence of any commercial or financial relationships that could be construed as a potential conflict of interest.

## Footnotes

1 Due to an administrative error, one instantiation of honesty, “borrowing money and giving it back,” was presented twice (to different participants) in the British sample, whereas “returning money which you have found or wrongly received” was not presented.

2 “Correct” is meant in a relative sense, based on the findings of Study 1 and our theoretical reasoning. Of course, there are no de facto right and wrong answers in tasks like this.
