# Supplementary material for: Cross-Cultural Differences and Similarities in Human Value Instantiation
Source: Front Psychol. 2018 May 29;9:849. doi: 10.3389/fpsyg.2018.00849 (PMC5987738; doi:10.3389/fpsyg.2018.00849)
Supplement: Supplementary file 1 [file Table_1.DOC]

**Supplemental Materials of “Cross-Cultural Differences and Similarities in Human Value Instantiation”**

Table of Contents

- Analyses of each Value (Study 1)
- Detailed Results of Study 1
- Detailed Results of Study 2

## Analyses of each Value (Study 1)

In the following paragraphs, the results of the above analysis are described for each value separately, comparing Brazil, India, and the UK. In parentheses next to each instantiation, we list the number of times in total it was mentioned, followed by the number of people who gave the responses. By presenting these findings in text, we enable the reader to reflect on the numbers in evaluating the comparisons that we make. This inclusion is important because the comparisons we make in the qualitative data (e.g., X instantiation was mentioned more in Y country than Z country) are necessarily speculative, and the numbers better support some comparisons than others. (Quantitative chi-square tests are not appropriate because this analysis is exploratory and any *p* values would need to correct for an unknown number of comparisons, which reflects an *a priori* testing mode that is not the aim in this study.) By providing the actual numbers in text, we aim to provide a more thorough and integrated reflection on the findings. Nonetheless, for readers interested in a tabular summary, the instantiations below are listed alongside their frequencies in Appendix A.

Protecting the environment. For British participants, typical instantiations were recycling (mentioned in total 18 times by 14 people), putting rubbish in the bin (15/8), switching off the lights (13/9), and reducing carbon emissions (11/8). These instantiations were also mentioned by Brazilian participants (19/5, 69/21, 2/2, 3/3) but rarely by Indian participants (0/0, 2/1, 0/0, and 0/0). For Brazilian participants, other typical instantiations were not wasting or polluting water (20/14), and that companies should not pollute the environment (18/9). These two instantiations were less often mentioned by British (5/5 and 5/4) and Indian participants (3/2 and 0/0). Indian participants frequently mentioned keeping the environment clean (12/6). Clean was mentioned less often by British (5/4) and Brazilian (0/0) participants. Overall, few of the typical instantiations in Brazil and the UK were mentioned by Indian participants, indicating that the instantiations are not typical in India.


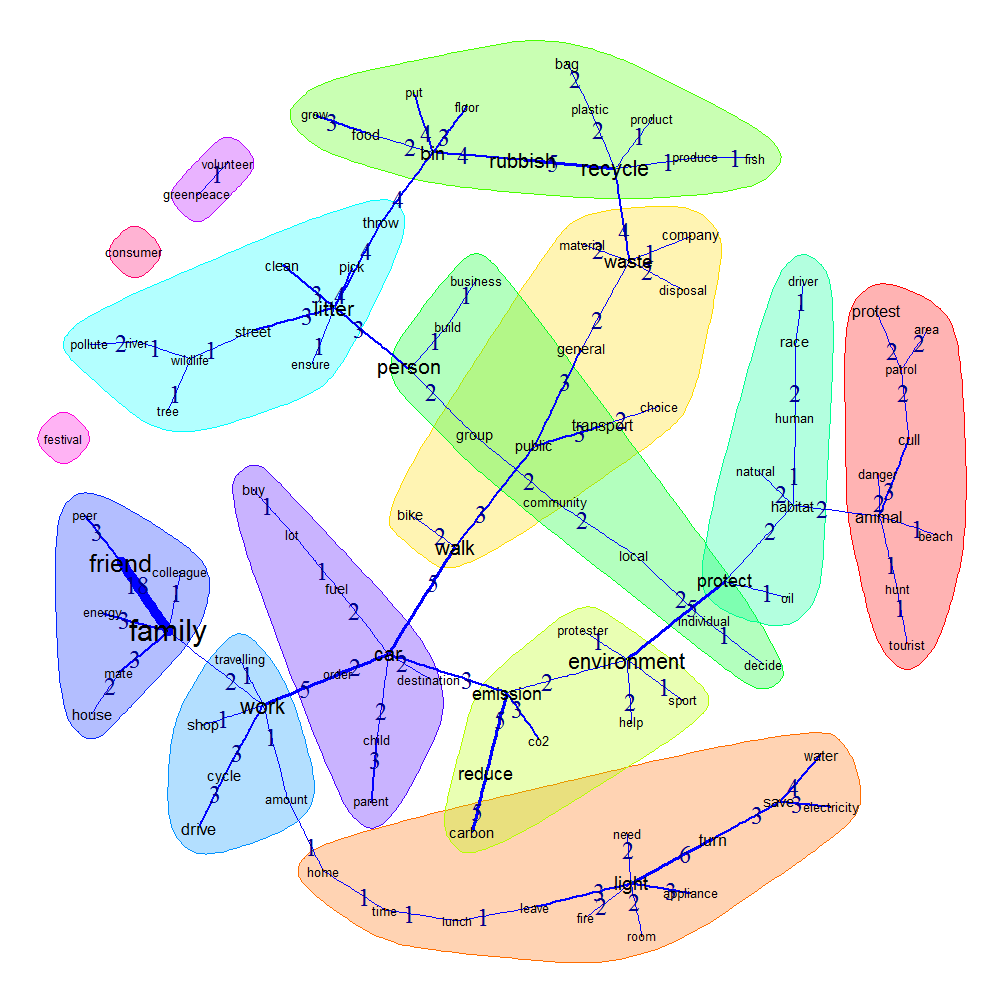


*Figure S1*. Result of the graphical similarity analysis for 'Protecting the environment' (UK only). Numbers indicate how often two words were mentioned together in single responses. Shared colours indicate that words were mentioned together.

The context and frequency of all the words for the UK sample can be seen in Figure S1. As for instantiations of most values, friends and family were mentioned often together. In the cluster at the bottom, for example, the words “lights”, “turn”, and “save”, in the cluster at the top the words “bin”, “rubbish”, and “recycle” were often mentioned together, supporting the aforementioned typical instantiations.

Wisdom. For British participants, typical instantiations were giving advice, for example to students or children (30/18) or making important decisions (17/10). In contrast, these two instantiations were not emphasized strongly in the Brazilian (7/4 and 4/4) or Indian (0/0 and 0/0) samples. Instead, Brazilian participants identified wisdom as being important for improving a difficult situation, as in conflict solving (16/7) more often than British and Indian participants did. In the Indian sample, no clear pattern was recognizable.

Unity with Nature. For British participants, typical instantiations were walking outside in nature (25/12), feeding or watching birds (12/6), or being in the garden (e.g., gardening, 12/8). These instantiations were mentioned less often by Brazilian (6/4, 5/3, and 3/2) and Indian (0/0, 3/1, and 2/1) participants. Brazilian participants emphasized protecting nature (11/8) more often than British (6/3) and Indian (2/2) participants. Brazilian participants mentioned the beach as a typical place more often (19/10) than British (5/5) and Indian (0/0) participants, probably because João Pessoa, the city where the questionnaire was completed, is on the coast of the Atlantic Ocean and has a tropical climate. Finally, Brazilian participants mentioned taking care of animals more often as an example of unity with nature (22/8) than British (7/5) or Indian (5/3) participants. In the Indian sample, no clear pattern was recognizable.

World of Beauty. For British participants, typical instantiations were walking outside (21/11), waking on the beach (10/8), or going on holiday (12/10). All three instantiations were mentioned less often by Brazilian (3/2, 6/5, and 0/0), and Indian (0/0, 0/0 and 0/0) participants. For both Brazilian and Indian participants, no common theme was observed.

Broad-mindedness. For British participants, the only typical instantiation was meeting new people (14/9). This instantiation was not mentioned by Brazilian or Indian participants. Brazilian participants emphasized new ideas and opportunities (18/6), and that the society should be more open towards minorities such as homosexuals (15/6). These instantiations were mentioned less frequently by British (5/5 and 5/3) and Indian (0/0 and 1/1) participants. For Indian participants, a typical instantiation was to help others, especially poor people (10/7). This instantiation of broad-mindedness was rare within the British (2/2) and Brazilian (0/0) samples.

Social Justice. For British participants, typical instantiations were ensuring that justice is applied equally to all, including homosexuals and disabled people (23/6). As a typical situation or environment, jobs were mentioned (10/6). Both instantiations were mentioned less often by Brazilian (2/2 and 0/0) and Indian (0/0 and 0/0) participants. Brazilian participants considered political rights like those related to health, education, and security as typical instantiations (27/14), regarded the government as responsible for reinforcing them (15/10) and education as a way to obtain equality (13/9). Those instantiations were mentioned less often by the British (13/5, 6/3, and 11/4) and Indian participants (0/0, 2/2, and 1/1). Indian participants considered helping as more typical (10/7) than British (2/2) and Brazilian (0/0) participants.

Equality. For British participants, typical instantiations were treating all children (13/6) and students (15/9) equally. Equality was also considered relevant during job applications and at the work place (24/12). The latter finding is consistent with observations by Maio et al. (2009). These three instantiations were also mentioned by the Brazilian (10/4, 6/5, and 9/6) and Indian (3/3, 16/11, and 25/8) participants. Brazilian participants frequently mentioned equal opportunities for all (23/14), including black people (13/6) and women (11/6). These topics were less often mentioned by British (1/1, 0/0, and 9/4) and Indian (3/3, 0/0, and 12/8) participants. For Indian participants, typical instantiations were giving equal opportunities to various subgroups (15/9) and doing this independent of caste affiliation (castism; 17/8). These two instantiations were less often mentioned by British (6/5 and 0/0) and Brazilian (6/3 and 0/0) participants.

Creativity. For British participants, typical instantiations were making or creating art (40/25), writing a book, poem, or essay (29/18), or making or composing music (22/16). All three instantiations were mentioned less often by Brazilian (5/4, 10/5, 3/2) and Indian (2/2, 3/2, and 0/0) participants. Brazilian participants focused on new (18/13) and different (11/5) things, such as ideas and products. Companies were mentioned as typical places (13/10). These three instantiations were less often mentioned by British (29/14, 7/2, and 2/2) and Indian (3/2, 6/5, and 3/2) participants. Finally, Indian participants frequently indicated that being creative is useful to become happy or to solve problems (20/8). Students were mentioned as typical people relevant to creativity (12/7). Both instantiations were mentioned less often by British (28/17 and 19/14) and Brazilian (19/9 and 11/7) participants. A more detailed comparison of the responses given by Brazilian and British participants can be found in Chapter 3, Study 8.

Freedom. For British participants, freedom rights were frequently mentioned. Of interest, they emphasized positive liberty rights (freedom *to*; 34/20) more than negative freedom rights (freedom *from*; 2/1). Job and work were mentioned as situations where freedom is relevant (17/10). All three instantiations were mentioned less often by Brazilian (13/10, 3/3, and 6/4) and Indian (15/8, 4/4, and 3/3) participants. In addition to mentioning liberty rights, Brazilian participants frequently mentioned travelling (11/8) as an exemplar of freedom and did so more often than British (7/6) and Indian (2/2) participants. A typical instantiation for Indian participants was that students need more freedom, especially from teachers (14/7). Students’ need for more freedom in general was also mentioned by British (11/8) and Brazilian (6/5) participants.

A Varied Life. For British participants, typical instantiations were doing varied activities at work (37/20), doing different and new activities (12/9), and having new experiences (12/7). The latter two instantiations were mentioned less often by Brazilian (0/0, and 2/2) and Indian (0/0, and 1/1) participants, but doing varied activities at work was mentioned at least somewhat frequently in Brazil (17/7) and India (7/4). In addition, Brazilian participants considered trying new things and meeting new people (21/8) and travelling (12/9) as typical instantiations of a varied life. Those two instantiations were mentioned less often by British (19/8 and 5/4) and Indian (16/6 and 0/0) participants. For Indian participants, no pattern was recognizable.

Daring. In none of the three countries did a clear pattern emerge. The written responses suggested some confusion about the meaning of the value, especially for the Brazilian participants.

Pleasure. For British participants, typical instantiations of pleasure were enjoying various things (29/15), eating (24/14), and drinking (14/12). All three instantiations were mentioned less often by Brazilian (2/1, 14/10, and 7/5) and Indian (7/4, 1/1, and 2/1) participants, although eating was also mentioned frequently in the Brazilian sample. In addition, Brazilian participants considered spending time with friends (65/21), family (32/18), and the boy- or girlfriend (i.e., partner; 21/9) as typical people relevant to pleasure. These three instantiations were also mentioned by British participants (59/25, 38/20, and 27/14) and to a lesser degree by Indian participants (18/12, 7/5, and 3/3). For Indian participants, no pattern was recognizable.

Success. For British participants, working (33/16) was a typical activity relevant to success. The most frequently mentioned people were students (28/19), and the common activities were exams (22/14) and sport (17/11). To some extent, these instantiations were also mentioned by Brazilian (8/6, 4/3, 3/2, and 2/2) and Indian (13/11, 9/7, 6/3, and 0/0) participants. For Brazilian participants, typical instantiations were companies (15/8), studying (14/11), and passing the entrance tests (12/9) for public positions, which everyone who wants to work in the popular public sector in Brazil has to pass. The first two of these instantiations were also mentioned by British (6/4, 7/6, and 0/0) and Indian (2/2, 7/6, and 0/0) participants. A typical instantiation for Indian participants was working hard (12/11). This instantiation was mentioned by British (14/7), but not by Brazilian (0/0) participants.

Ambition. For British participants, frequently mentioned typical instantiations were work (56/28), achieving your goals (15/10), and (sport) team (13/7). These instantiations were less often mentioned by Brazilian (20/11, 0/0, and 0/0) and Indian (32/16, 5/4, and 0/0) participants. For Brazilian participants, a typical ambition was having a good family life (14/7). This instantiation was mentioned less often by British (2/2) and Indian (0/0) participants. Typical instantiations for Indian participants were getting a good job (18/11), education (17/8), and working hard (11/8). These instantiations were less often mentioned by British (3/3, 3/3, and 13/9) and Brazilian (1/1, 0/0, and 0/0) participants.

Wealth. For British participants, typical instantiations of wealth were buying various, mainly expensive things (24/11) and shopping (12/8). Children were identified as typical people (e.g., “to provide children with what they need”, 13/6). These instantiations were also mentioned by Brazilian (4/3, 4/3 and 7/4) and Indian (5/4, 0/0, and 8/4) participants. For Brazilian participants, a typical instantiation was health (10/7). This instantiation was mentioned less often by British (1/1) and Indian (6/5) participants. A typical instantiation for Indian participants was to live a good life (11/8), which was mentioned less often by British (2/2) and Brazilian (9/8) participants.

Social Power. British participants identified the police (23/10) and teachers/professors/lecturers (18/10) as typical groups of people holding social power, and voting (12/8) as a typical situation or behaviour relevant to the value. These instantiations were mentioned less often by Brazilian (1/1, 8/4, and 4/2) and Indian (0/0, 0/0, and 3/3) participants. For Brazilian participants, typical instantiations were society in general as typical people (whereas a society has social responsibilities; 23/6), children as typical people (13/6); (liberty) rights (12/9) were also frequently mentioned. These instantiations were mentioned less often by British (1/1, 12/5, and 3/3) and Indian (5/2, 4/1, and 9/5) participants. Indian participants frequently mentioned work (10/5) as a typical situation relevant to social power, and this instantiation was also mentioned by British (14/11) and Brazilian (6/3) participants.

*Family Security*. For British participants, support was a typical behaviour (14/11), and the people mentioned were parents (including mother and father, 32/10) and children (including daughter and son, 26/9). Brazilian (10/5, 71/16, 37/11) and Indian (0/0, 11/5, 15/6) participants followed a similar pattern. Brazilian participants also considered securing the family home against intruders (through electronic fences, demanding more police on the street; 15/7) as typical, whereas this was not mentioned by British (0/0) or Indian (2/1) participants. No other instantiations arose frequently among the Indian participants.

Respect for Tradition. British participants frequently mentioned Christmas (24/16), church (19/15), and eating together (16/9). These instantiations were mentioned less often by Brazilian (6/4, 4/3, and 7/4) and Indian (all 0/0) participants. For Brazilian participants, parents (34/10) and children (29/12) and the city (10/5) were mentioned often. (Cities are the centre for a local festival.) Parents were mentioned by British (18/9) and, to a lesser extent, Indian (6/3) participants, but children and cities were not mentioned as often among British (4/4, 0/0) and Indian (6/3, 0/0) participants. Typical instantiations for Indian participants were wearing traditional dress (16/10) and festivals (13/9). These instantiations were mentioned somewhat less often by British (6/4 and 5/4) and Brazilian (4/3 and 11/8) participants, although Brazilian participants also mentioned festivals frequently (and their locations in cities; see above).

Self-discipline. British participants identified the importance of self-discipline in work (34/18), exercising (19/12), and losing weight (17/13). Work was also identified frequently by Brazilian (25/12) and Indian (16/10) participants, whereas exercising and losing weight were mentioned less often by Brazilian (2/2 and 0/0) and Indian (0/0 and 0/0) participants. For Brazilian participants, lecturer/professor/teacher was a typical person (12/7), but such persons were mentioned less often by British (4/4) and Indian (8/5) participants. Indian participants frequently mentioned students (13/11). This instantiation was also mentioned often by British (19/14) and Brazilian (16/11) participants.

*Obedience*. British participants mentioned children (31/14), parents (27/14), and teachers (24/15) as typical people relevant to obedience, mostly in the sense that children should obey their parents and teachers. A similar pattern was shown by Brazilian (32/16, 63/21, and 22/11) and, to a lesser extent, Indian (5/4, 15/10, and 16/12) participants. Brazilian participants also frequently mentioned the verb to ask (20/10) and rules (20/10). These instantiations were mentioned less often by British (0/0 and 13/8) and Indian (2/2 and 0/0) participants, although British participants did mention rules frequently. Indian participants identified work (19/9) as a typical situation or activity and students (14/10) as typical people. These instantiations were also mentioned by British (13/9 and 21/10) and Brazilian (4/4 and 21/12) participants.

*Helpfulness*. British participants frequently connected helpfulness with work (13/10), students (12/7), and customers (11/5), as did Brazilian participants (40/18, 21/12, and 8/5), but not Indian participants (2/2, 3/3, and 0/0). For Brazilian participants, typical people were colleagues/classmates (24/9) and elderly (18/11), while the street (17/11) was a typical place. These instantiations were mentioned less often by British (6/5, 8/6, and 1/1) and Indian (1/1, 0/0, and 0/0) participants. No typical instantiations for Indian participants were found.

*Loyalty*. British participants often mentioned work (22/12), relationships (19/14), (providing) support (18/12), and (keeping) secrets (10/6). These instantiations were also mentioned by Brazilian (25/11, 3/3, 4/3, and 2/2) and Indian (5/4, 5/4, 1/1, and 2/2) participants. Brazilian participants also frequently mentioned (business) companies (17/8), colleagues (13/5), and husband-and-wife (15/7). These instantiations were less often mentioned by British (6/4, 15/7, and 4/1) and Indian (1/1, 0/0, and 10/4) participants, aside from British references to colleagues and Indian references to couples. In both countries, being loyal to friends was mentioned at least three times as often as being loyal to any other group of people. No typical instantiations for Indian participants were found.

*Honesty*. Typical instantiations of honesty for British participants were relationships (31/21), not cheating (13/9), and the courtroom (11/8). These instantiations were mentioned less often by Brazilian (7/5, 0/0, and 0/0) and Indian (3/3, 2/2, and 1/1) participants. For Brazilian participants, typical instantiations included money (21/10) and returning money (mostly money found on the street or after receiving too much change, 15/8). These instantiations were less often mentioned by British (2/2 and 0/0) and Indian (8/5 and 7/4) participants. Typical instantiations for Indian participants were parents (15/10) and work (10/6), which were also mentioned by British (8/4 and 7/6) and Brazilian (14/5 and 12/8) participants.

## Detailed Results of Study 1

Words which were mentioned by at least 20% of the participants were analysed. Because the sample sizes differed, the number of participants who mentioned an instantiation varied. Quotation marks indicate that a response of a participant is listed. Numbers in brackets indicate how easy it was to discover the meaning of the specific word. 1: very much variance between the answers with the given keywords (no pattern among the responses is recognizable), 5: very little variance (i.e. the answers are all very similar in their meaning). Figures are based on the results from the UK.

**Protecting the environment**

Table **1**

***UK results of university sample for Protecting the environment***

| Word | Meaning (Instantiation) | Absolute Frequency |
| --- | --- | --- |
| family | “family and friends” as relevant people (5) | 30 |
| friend | “family and friends” as relevant people (5) | 22 |
| recycle | “recycling”, “putting certain rubbish in recycle bins rather than general waste” (5) | 18 |
| work | “cycling to and from work instead of driving” (3) | 18 |
| environment | “protect the environment” (4) | 17 |
| car | share the car, walk instead of using car for short distances (3) | 16 |
| person | people who try to protect the environment (2) | 16 |
| rubbish | “putting rubbish in the bin not the floor” (4) | 16 |
| litter | “putting rubbish in the bin not the floor” (4) | 16 |
| walk | “travelling to work etc. using public transport bikes or walking rather than using a car” (3) | 15 |
| light | “making sure the lights are off” (4) | 15 |
| bin | using bins (3) | 15 |
| waste | “disposal of waste”, recycling waste (3) | 13 |
| protect | “protect the environment” (4) | 12 |
| emission | “reducing carbon emissions” (4) | 11 |
| reduce | “reducing carbon emissions” (4) | 11 |
| animal | “culling of animals”, “preserving animal habitats” (2) | 10 |

Table **2**

***Brazil results of university sample for Protecting the environment***

| Word | Meaning (Instantiation) | Absolute Frequency |
| --- | --- | --- |
| garbage | "Recyclables". "Garbage in bin" (5) | 69 |
| play | "Throwing trash in the trash" (5) (subset of “garbage”) | 39 |
| environment | "Environment" (5) | 30 |
| environment | "Environment" | 27 |
| water | "Save water" control water consumption (4) | 20 |
| street | "Helping homeless animals." "Throwing garbage in the street" (3) | 19 |
| business | "Companies that look after the environment" situations to punish companies that pollute (4) (but people do not think about this while shopping -> hypocritical) | 18 |
| population | "People throw trash in the bin." "Population separating garbage" situations where the people throw trash enquiry (5) | 15 |
| home | "Decorate your home so you do not pollute" situations of water saving (4) | 14 |
| animal | "Helping animals" (2) | 13 |
| protect | "Protect the environment" (5) | 13 |
| all | "All people" (1) | 13 |
| pollution | "Pollution of rivers”. "Noise" (4) | 12 |
| use | "Using water" situations of water use (5) | 12 |
| car | "Riding car" (4) | 10 |
| avoid | "Avoiding polluting the environment" (5) | 10 |
| plastic | "Plastic bags" (3) | 10 |
| pollute | "Polluting the environment" (4) | 10 |
| beach | "Take the garbage thrown into the beach" (4) | 10 |
| projects | "Public initiative projects of clean cities" situations where it asks for the development of projects to protect the environment (4) | 10 |
| river | "Littering the rivers." "River sewage" (3) | 10 |
| trees | "Cutting trees." "Toppling trees" (4) | 10 |

Table 3

India results of university sample for Protecting the environment

| Word | Meaning (Instantiation) | Absolute Frequency |
| --- | --- | --- |
| person | “people” (3) | 28 |
| clean | “cleaning”, “keep clean” (4) | 12 |

**Equality**


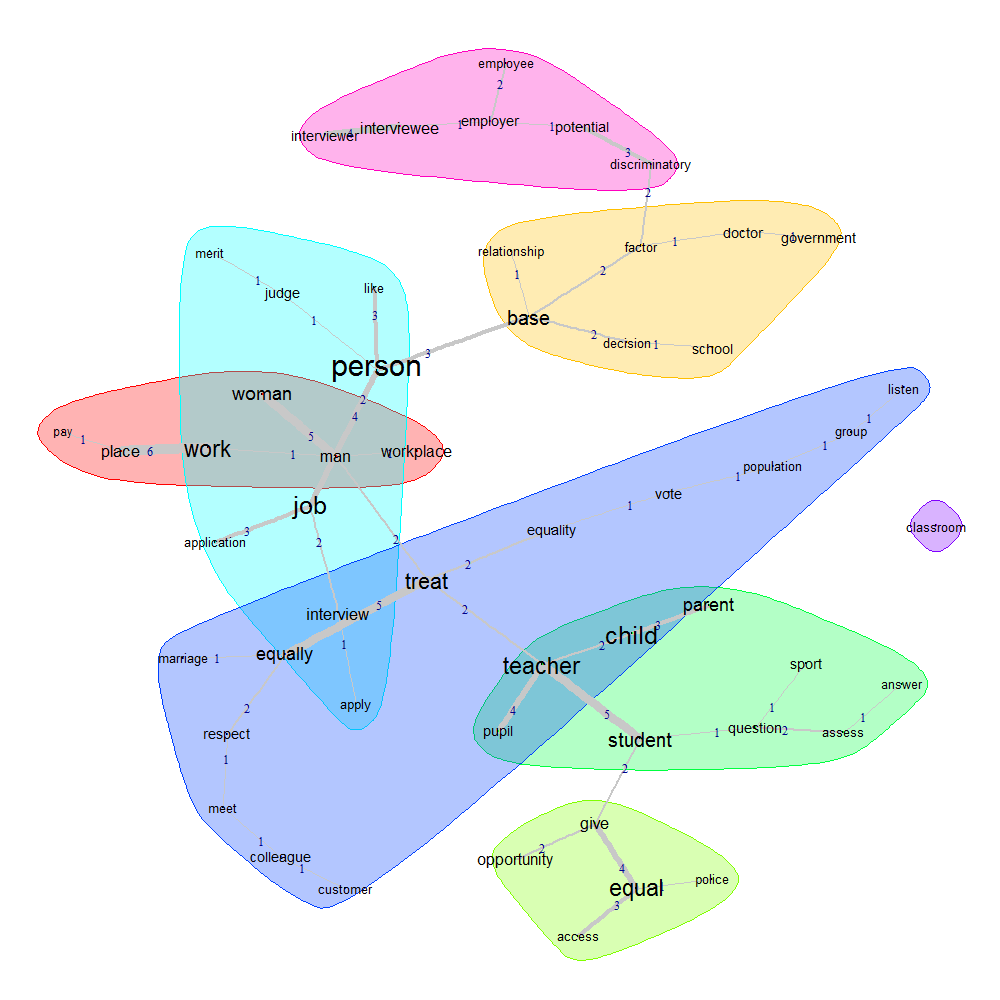


*Figure* ***1***. Result of the graphical similarity analysis for 'Equality' (UK only). Numbers indicate how often two words were mentioned together in single responses. Same colours indicate that words were mentioned together.

Table **4**

***UK results of university sample Equality***

| Word | Meaning (Instantiation) | Absolute Frequency |
| --- | --- | --- |
| person | (no pattern recognizable) | 18 |
| child | “all children are allowed to use all equipment, given the same opportunities”; treating all children equally (4) | 13 |
| job | “job application”, “job interview” (4) | 13 |
| teacher | “teacher” as relevant person in the situation (4) | 12 |
| equal | equal rights and equal opportunities (5) | 11 |
| student | “students/pupils” as relevant people in the situation (3) | 11 |
| work | “work place” as typical situation, “men and women should be treated equally in the work place” (4) | 11 |
| treat | “treat all pupils equally” (3) | 10 |

Table **5**

***Brazil results of university sample Equality***

| Word | Meaning (Instantiation) | Freq. |
| --- | --- | --- |
| all | "Everyone deserves an equal chance." 'Everyone is entitled to an opportunity "(4) | 23 |
| same | "The opportunity is the same for both sexes." "Everyone being treated the same way regardless of the preference of sex" (4) | 19 |
| right | "Claiming your rights." "A university should be an equal right for all people." "Same rights for everyone regardless of color" (5) | 13 |
| black | "The common people in favor of mostly blacks" "abolish the black and white racism" (3) | 13 |
| woman | "Rights for men and women." "That woman and man have no difference" (4) | 11 |
| quota | "University quotas for blacks with Indians and mestizos." "Quota for disabled" | 10 |
| child | "Children with mental problems." "Children and young people who are in a state of obesity" (4) | 10 |
| man | "Men and women in work situations." "Law so that men and women have no difference" (3) | 10 |
| mother | "Mother sharing food" (2) | 10 |
| public | "entrance exames for public positions for all races" (3) | 10 |
| your | "Right to pronounce your ideas." "Each performs its function" (3) | 10 |

Table 6

*India results of university sample for Equality*

| Word | Meaning (Instantiation) | Absolute Frequency |
| --- | --- | --- |
| Person | People from various casts, religious backgrounds, or socio-economic status (3) | 38 |
| Cast(ism)/categories/sc and st | Discrimination based on cast (5) | 17 |
| Work | working hard, equal work (3) | 17 |
| Friend | Friends and family (3) | 16 |
| Student | Students and castism (3) | 16 |
| Give | Giving equal opportunities (4) | 15 |
| Treat | Treat everyone equally (5) | 12 |
| Women | Women equality (4) | 12 |
| Equal | Treat people equal (4) | 11 |
| Time | (no pattern recognizable) | 10 |

**Wisdom**


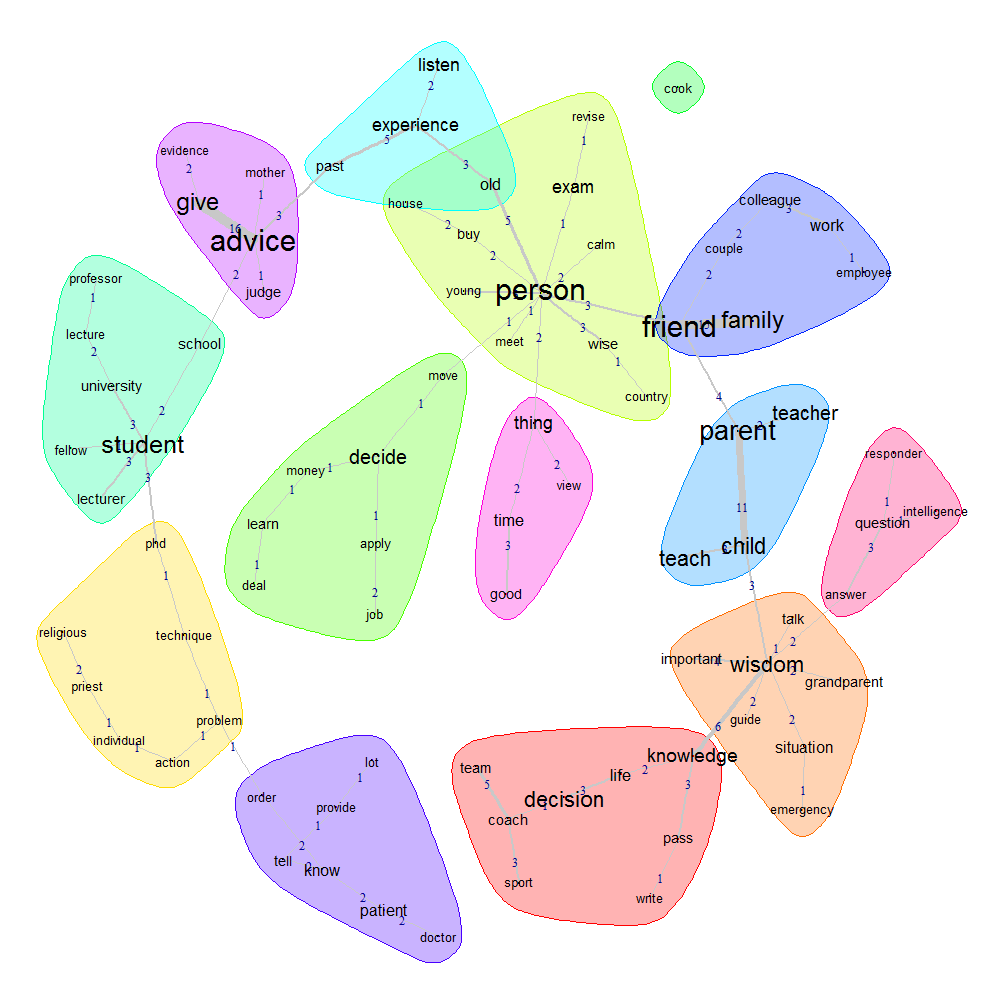


*Figure* ***2*.** Result of the graphical similarity analysis for 'Wisdom' (UK only). Numbers indicate how often two words were mentioned together in single responses. Same colours indicate that words were mentioned together.

Table **7**

***UK results of university sample for Wisdom***

| Word | Meaning (Instantiation) | Absolute Frequency |
| --- | --- | --- |
| person | (no pattern recognizable) | 35 |
| advice | “giving advice” (4) | 30 |
| friend | “friends” as relevant people (5) | 29 |
| parent | “parents teaching their children about life” and “parents” as relevant people (4) | 25 |
| student | “giving students advice”, “students” as relevant people (4) | 22 |
| child | give guidance/pass knowledge to children, teaching children (4) | 20 |
| make | “making [important/serious] decisions” (4) | 20 |
| give | “Giving advice” (older person, friend) (5) | 19 |
| family | “family” as relevant people (5) | 19 |
| decision | “making [important/serious] decisions” (4) | 17 |
| wisdom | “passing on their wisdom”, “having wisdom” (3) | 16 |
| teach | “teaching or explaining something” (3) | 16 |
| decide | deciding about something important (4) | 14 |
| teacher | “teacher” as relevant people (5) | 13 |
| experience | “using past knowledge and experiences” (4) | 13 |
| knowledge | using/sharing knowledge (3) | 12 |
| exam | “exam” as typical situation in which wisdom is important (5) | 12 |
| work | (no pattern recognizable) | 10 |
| thing | (no pattern recognizable) | 10 |
| listen | listening to other people (4) | 10 |
| life | (no pattern recognizable) | 10 |

Table **8**

***Brazil results of university sample for Wisdom***

| Word | Meaning (Instantiation) | Absolute Frequency |
| --- | --- | --- |
| friend | "Receiving advise from friends" (4) | 17 |
| wisdom | "Wisdom to deal with conflicts" wisdom to solve conflicts (5) | 16 |
| know | "How to deal with awkward situations" (4) | 13 |
| other | "Talk to others" (3) | 11 |
| its | "Wisdom" (3) | 11 |
| knowledge | "Spread your knowledge" use knowledge to relate to other people | 10 |

Table 9

*India results of university sample for Wisdom*

| Word | Meaning (Instantiation) | Absolute Frequency |
| --- | --- | --- |
| Person | (no pattern recognizable) | 23 |

**Unity with nature**


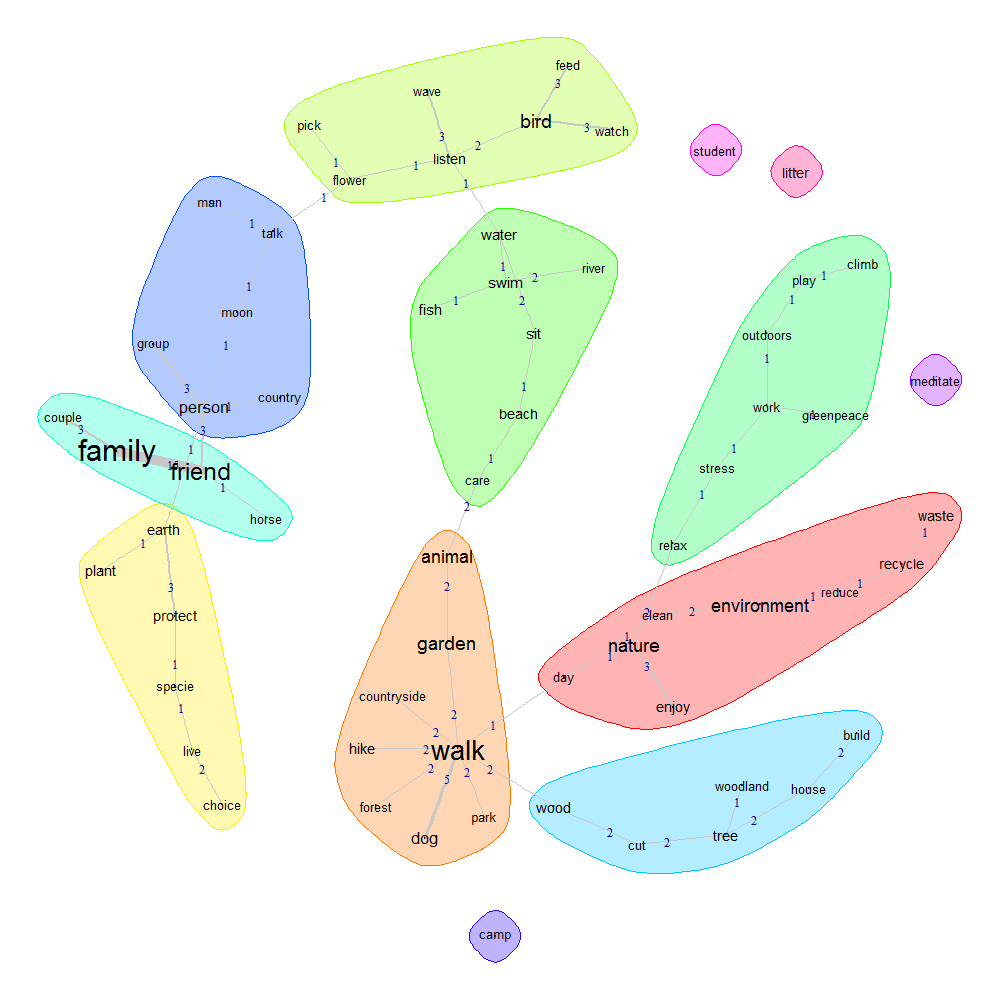


*Figure* ***3***. Result of the graphical similarity analysis for ‘Unity with Nature'. Numbers indicate how often two words were mentioned together in single responses. Same colours indicate that words were mentioned together.

Table **10**

***UK results of university sample for Unity with nature***

| Word | Meaning (Instantiation) | Absolute Frequency |
| --- | --- | --- |
| family | “family” as relevant people (5) | 28 |
| walk | “walking the dog”, walking outside (5) | 25 |
| friend | “friend” as relevant people (5) | 20 |
| nature | “being in nature” (2) | 12 |
| garden | being in the garden (5) | 12 |
| bird | “bird watching”, “feeding birds” (5) | 12 |
| environment | “looking after the environment” (3) | 11 |
| animal | “observing animals”, “helping animals” (4) | 11 |
| person | (no pattern recognizable) | 10 |

Table **11**

***Brazil results of university sample for Unity with nature***

| Word | Meaning (Instantiation) | Absolute Frequency |
| --- | --- | --- |
| nature | "Preserve nature", "closer to nature" (5) | 40 |
| friend | "Meet friends" meet with friends in places with nature (forests and beaches) (4) | 28 |
| animal | "Animal care" (4) | 23 |
| all | Refers to humans and animals (2) | 23 |
| plant | "Plant Trees" planting trees with friends (5) | 19 |
| beach | "Walking at the beach" walk along the beach with friends, family and boyfriends / girlfriends (4) | 19 |
| other | "etc." idea of variety (2) | 16 |
| care | "Animal care" (5) | 15 |
| garbage | "Garbage disposal" In which situations it discards the trash Correctly (4) | 14 |
| same | "Himself" myself (2) | 11 |
| environment | "Do not pollute the environment" situations of environmental protection (5) | 10 |
| familiar | "Weekend with relatives in the countryside" enjoy nature with family | 10 |
| yours | "Taking care of the animals that are under your care" animal care (2) | 10 |

Table 12

*India results of university sample for Unity with Nature*

| Word | Meaning (Instantiation) | Absolute Frequency |
| --- | --- | --- |
| Family | (no pattern recognizable) | 13 |
| Friend | (no pattern recognizable) | 13 |
| Nature | (no pattern recognizable) | 13 |
| Person | (no pattern recognizable) | 13 |

**World of Beauty**


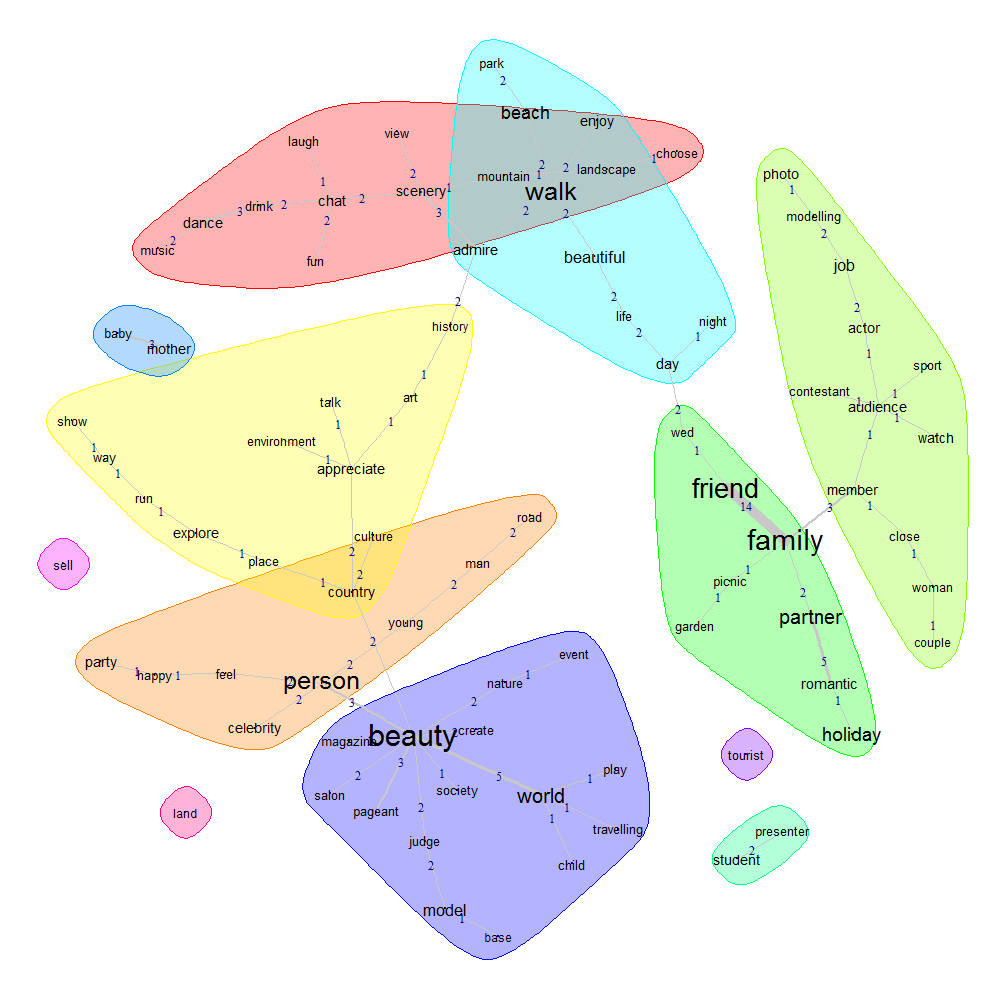


*Figure* ***4***. Result of the graphical similarity analysis for ‘World of Beauty'. Numbers indicate how often two words were mentioned together in single responses. Same colours indicate that words were mentioned together.

Table **13**

***UK results of university sample for World of Beauty***

| Word | Meaning (Instantiation) | Absolute Frequency |
| --- | --- | --- |
| beauty | “beauty pageants” (3) | 29 |
| Family | “family” as relevant people (5) | 26 |
| Friend | “friend” as relevant people (5) | 23 |
| Person | (no pattern recognizable) | 23 |
| Walk | walking outside (4) | 21 |
| world | (no pattern recognizable) | 14 |
| partner | “partner” as relevant people (5) | 13 |
| holiday | “holiday” as typical situation (4) | 12 |
| model | “models celebrities” (4) | 10 |
| beach | “beach” as typical place (4) | 10 |

Table **14**

***Brazil results of university sample for World of Beauty***

| Word | Meaning (Instantiation) | Absolute Frequency |
| --- | --- | --- |
| be | (no pattern recognizable) | 29 |
| beauty | "Natural Beauties" (2) | 25 |
| my | "My Family" (3) | 23 |
| world | "Beautiful World". "World security". "Better world" (3) | 18 |
| friend | "Friends enjoying." "Friends having fun." Friends talking "(5) | 17 |
| family | "Playing family". "With my family enjoying life" (5) | 15 |
| all | "All people" (3) | 15 |
| child | "Working children" "needy children" (4) | 13 |
| woman | "Beautiful women" pleasant situations with women (4) | 12 |
| care | "Take care of his own body." be careful with the body and family (4) | 10 |
| its | "Activities" (3) | 10 |
| life | "Healthier life" (4) | 10 |

Table 15

India results of university sample for World of Beauty

| Word | Meaning (Instantiation) | Absolute Frequency |
| --- | --- | --- |
| Person | (no pattern recognizable) | 26 |
| Nature | Protect nature (3) | 13 |
| Beauty | Beauty of nature (2) | 12 |
| Friend | Friends and family (3) | 11 |

**Broad-mindedness**


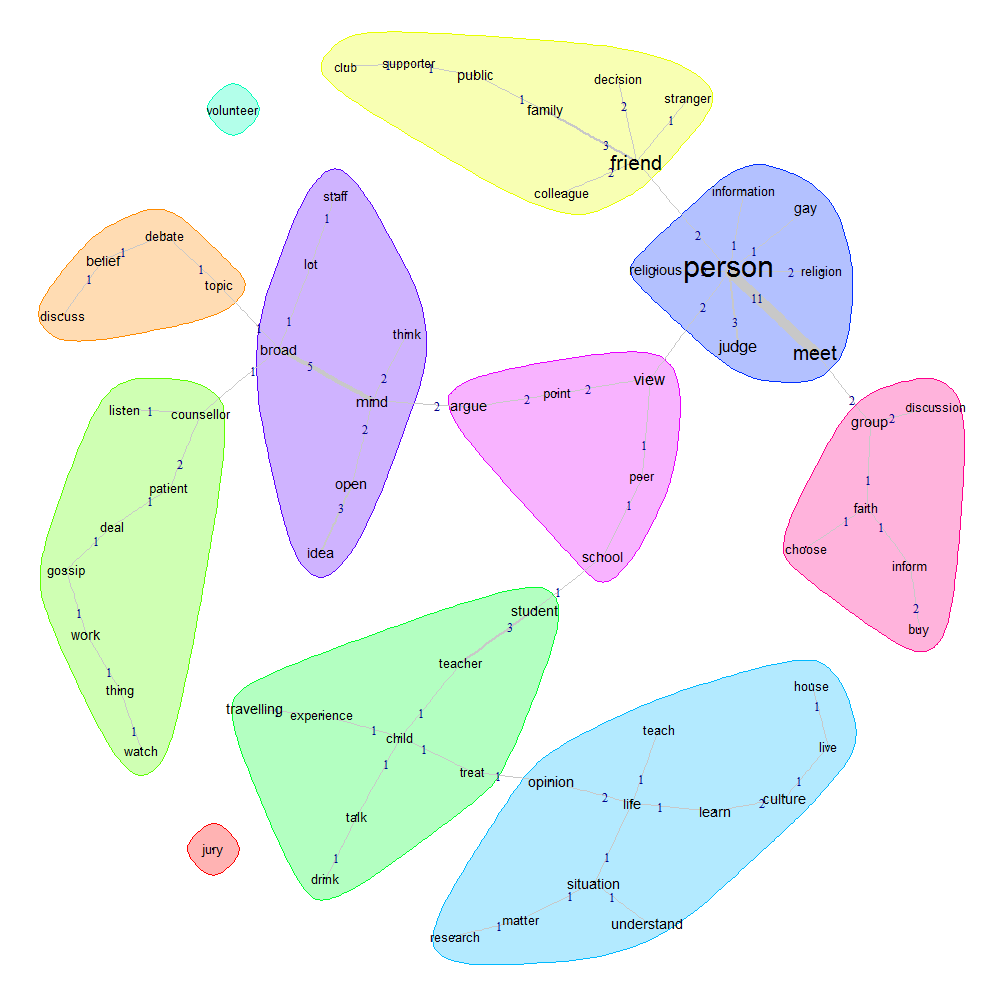


*Figure* ***5****.* Result of the graphical similarity analysis for 'Broad-mindedness'. Numbers indicate how often two words were mentioned together in single responses. Same colours indicate that words were mentioned together.

Table **16**

***UK results of university sample for Broad-mindedness***

| Word | Meaning (Instantiation) | Absolute Frequency |
| --- | --- | --- |
| person | “meeting new people” (3) | 34 |
| friend | “friend” as relevant people (2) | 17 |
| meet | “meeting new people” (4) | 15 |

Table **17**

***Brazil results of university sample for Broad-mindedness***

| Word | Meaning (Instantiation) | Absolute Frequency |
| --- | --- | --- |
| friend | "Friends living the moment" situations of drug use, homosexual relationships and talking controversial topics (3) | 20 |
| new | "New ideas". "New opportunities" (5) | 18 |
| all | "All accepting". "Everyone debating" (4) | 16 |
| society | "Society against homophobia". "Society against bullying" situations against prejudice (4) | 15 |
| its | "Your life." "Ideas" (4) | 13 |
| son | "Gay Son" situations against prejudice (5) | 12 |
| other | "Another sexual orientation" (4) | 12 |
| life | "Better life" (3) | 11 |
| father | "Father and grandparents are dedicated to teaching" situations where parents accept the differences of the sounds (3) | 10 |
| its | "Your Dream". "Their lifestyle" (2) | 10 |

Table 18

India results of university sample for Broad-mindedness

| Word | Meaning (Instantiation) | Absolute Frequency |
| --- | --- | --- |
| Person | “helping people” (3) | 24 |
| Friend | Family and friends (3) | 17 |
| Family | Family and friends (3) | 16 |
| Help | Helping (poor) people (4) | 10 |

**Social Justice**


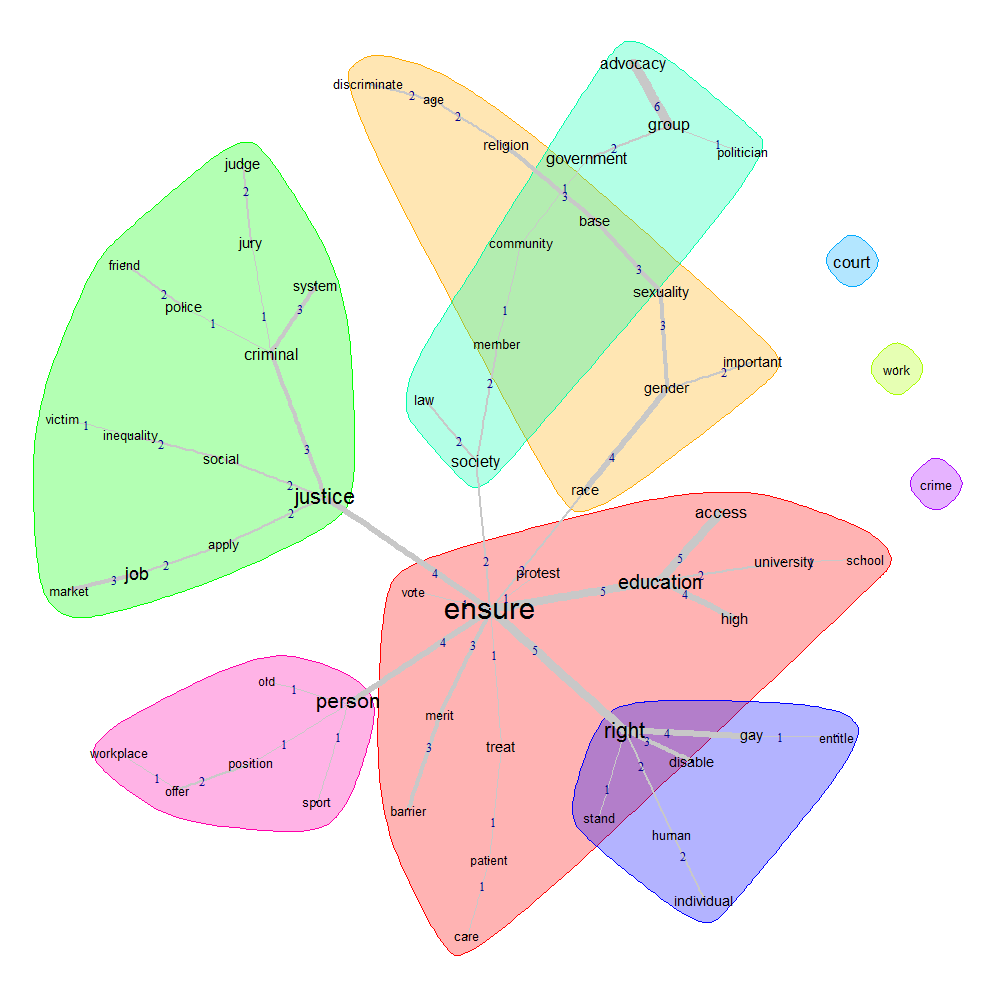


*Figure* ***6.*** Result of the graphical similarity analysis for ‘Social Justice’. Numbers indicate how often two words were mentioned together in single responses. Same colours indicate that words were mentioned together.

Table **19**

***UK results of university sample for Social Justice***

| Word | Meaning (Instantiation) | Absolute Frequency |
| --- | --- | --- |
| ensure | “ensuring that justice is applied equally to all” (4) | 23 |
| person | (no pattern recognizable) | 14 |
| justice | “making sure that people treated wrongly get justice over those that did them wrong” (3) | 13 |
| right | “gay rights”, “disabled rights” (4) | 13 |
| education | “ensuring that there is no barrier other than merit in access to higher education” (4) | 11 |
| job | “applying for job” (2) | 10 |

Table **20**

***Brazil results of university sample for Social Justice***

| Word | Meaning (Instantiation) | Absolute Frequency |
| --- | --- | --- |
| right | "Political rights" common related to health, education and security (5) | 27 |
| public | "Public services". "Public schools" (5) | 20 |
| all | "All people" (3) | 20 |
| government | "Government social policies" situations where the government Should help the population, eg enforce the law and providing public services (4) | 15 |
| population | "Working population". "People going to the streets" | 15 |
| political | "Corrupt politicians" (5) | 14 |
| education | "Education in schools". "Traffic education" (5) | 13 |
| teacher | "Primary school teachers" (4) | 13 |
| fight | "Fighting for the rights" situations against prejudice, injustice and crimes (4) | 12 |
| society | "Struggling society" / fighting against difficulties in society (3) | 11 |
| citizen | "Citizens demanding their rights" (5) | 10 |
| Seniors | "Elderly in homes for asylum" situations on the needs of the elderly and health care eg | 10 |

Table 21

India results of university sample for Social Justice

| Word | Meaning (Instantiation) | Absolute Frequency |
| --- | --- | --- |
| Girl | Discrimination of girls (5) | 10 |
| Person | (no pattern recognizable) | 10 |

**Creativity**


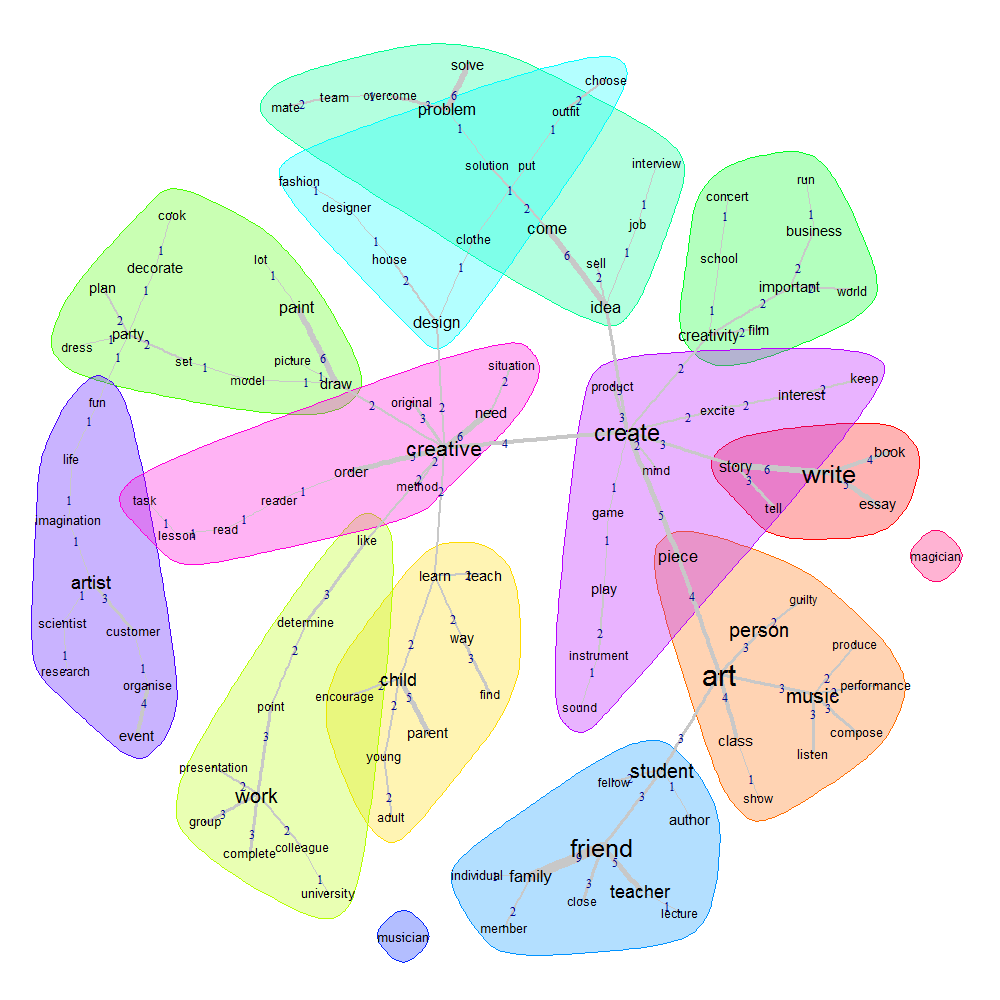


*Figure* ***7***. Result of the graphical similarity analysis for ‘Creativity’. Numbers indicate how often two words were mentioned together in single responses. Same colours indicate that words were mentioned together.

Table **22**

***UK results of university sample for Creativity***

| Word | Meaning (Instantiation) | Absolute Frequency |
| --- | --- | --- |
| art | “making art”, “art classes” (3) | 40 |
| write | writing an essay/book/poem (4) | 29 |
| friend | “friend” as relevant people (5) | 29 |
| create | “creating art” [including books] (4) | 28 |
| make | making something new (3) | 23 |
| music | “Making music”, “Composing music”, “listening to music” (4) | 22 |
| creative | Being/thinking creative is helpful/important (4) | 21 |
| work | “Working as a group in uni”, “When thinking of new ideas - Could be at work” (3) | 20 |
| person | “person” as relevant people (5) | 20 |
| student | “students” as relevant people (4) | 19 |
| child | important for children to learn new things (2) | 19 |
| artist | “artists” as relevant people (5) | 17 |
| teacher | “teacher” as relevant people (3) | 16 |
| idea | “coming up with novel ideas” (5) | 15 |
| family | “family” as relevant people (5) | 14 |
| come | “come up with new ideas” (5) | 13 |
| problem | “problem solving” (5) | 12 |
| piece | “creating a piece of art” (4) | 12 |
| paint | “painting” (5) | 12 |
| story | “writing a story” (4) | 11 |
| draw | “drawing” (4) | 11 |
| design | designing something new (3) | 11 |
| creativity | (no pattern recognizable) | 11 |
| need | (no pattern recognizable) | 10 |
| class | “art class” (4) | 10 |

Table **23**

***Brazil results of university sample for Creativity***

| Word | Meaning (Instantiation) | Absolute Frequency |
| --- | --- | --- |
| work | "Be original work" (5) | 23 |
| create | "Creating beautiful things." "Creating strategies to achieve the goal" (4) | 19 |
| new | "New projects". "Create new products." "Proposing new ideas" (4) | 18 |
| business | "Companies looking for new ways." "Companies seeking growth" (4) | 13 |
| friend | "Debating issues with friends" (4) | 12 |
| creativity | "Can reach a solution with creativity" (4) [‘The Brazilian way’] | 12 |
| your | "Improve their lives" (4) | 12 |
| different | "Seek different things" (4) | 11 |
| creative | "Needs to be very creative" (4) | 10 |
| day | "Scientific articles produced daily (3) | 10 |

Table 24

India results of university sample for Creativity

| Word | Meaning (Instantiation) | Absolute Frequency |
| --- | --- | --- |
| Creative | Being creative is useful | 20 |
| Person | (no pattern recognizable) | 16 |
| Friend | (no pattern recognizable) | 14 |
| Student | (no pattern recognizable) | 12 |

**Freedom**


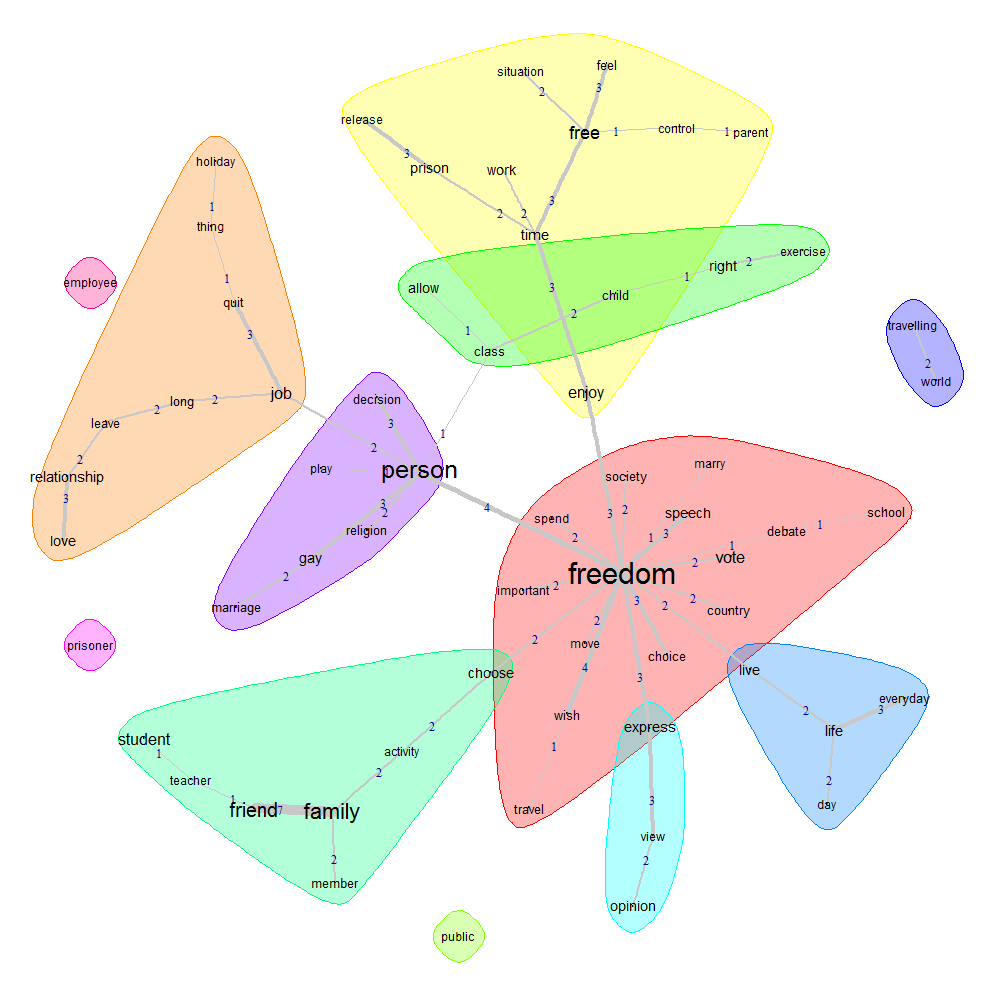


*Figure* ***8***. Result of the graphical similarity analysis for ‘Freedom’. Numbers indicate how often two words were mentioned together in single responses. Same colours indicate that words were mentioned together.

Table **25**

***UK results of university sample for Freedom***

| Word | Meaning (Instantiation) | Absolute Frequency |
| --- | --- | --- |
| freedom | freedom rights. [More positive than negative liberty rights mentioned] (4) | 36 |
| person | persons as relevant people (2) | 26 |
| family | “family” as relevant people (5) | 22 |
| friend | “friends” as relevant people (5) | 17 |
| free | “free to choose”, “free from the oppression” (3) | 14 |
| vote | “voting” (4) | 12 |
| job | “deciding on a job” (3) | 12 |
| student | “students” as relevant people (4) | 11 |
| enjoy | enjoying life (3) | 10 |
| choose | having the possibility to choose between different things (3) | 10 |

Table **26**

***Brazil results of university sample for Freedom***

| Word | Meaning (Instantiation) | Absolute Frequency |
| --- | --- | --- |
| People | "people knowing different places" (4) | 32 |
| Freedom | "freedom of speech" (4) | 21 |
| Friends | "friends and people knowing different places" (3) | 20 |
| your | "exercising their freedom." "Defending your opinion" (3) | 18 |
| want | "I want to travel" (2) | 11 |
| travel | "travel without a plan." "Traveling with friends". "Family travels to have fun" (4) | 11 |
| Young | "young adults and the elderly" (2) | 10 |
| All | "all cultures of freedom" (2) | 10 |

Table 27

India results of university sample for Freedom

| Word | Meaning (Instantiation) | Absolute Frequency |
| --- | --- | --- |
| Freedom | (no pattern recognizable) | 24 |
| Friend | (no pattern recognizable) | 18 |
| Family | (no pattern recognizable) | 17 |
| Person | (no pattern recognizable) | 16 |
| Student | Students need more freedom (4) | 14 |
| Time | (no pattern recognizable) | 10 |

**A varied life**


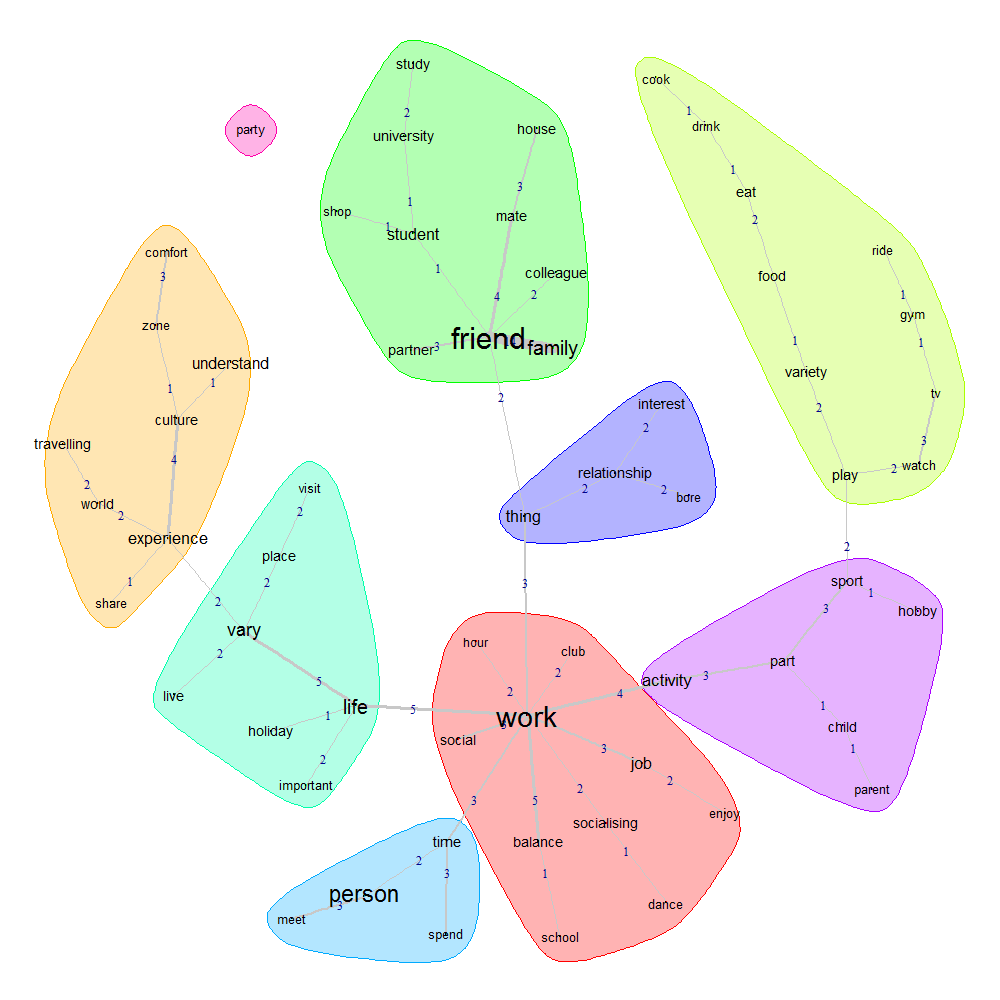


*Figure* ***9***. Result of the graphical similarity analysis for ‘A varied life’. Numbers indicate how often two words were mentioned together in single responses. Same colours indicate that words were mentioned together.

Table **28**

***UK results of university sample for A varied life***

| Word | Meaning (Instantiation) | Absolute Frequency |
| --- | --- | --- |
| work | “keep work life balance maintain”, “varying roles at work”, “balance school work and social life” (4) | 37 |
| friend | “friends” as relevant people (5) | 37 |
| person | “people” as relevant people (3) | 23 |
| life | “work life balance” (2) | 20 |
| family | “family” as relevant people (5) | 18 |
| vary | “varied life”, do varied things (5) | 14 |
| student | “students” as relevant people (3) | 12 |
| experience | gaining new/different experiences (4) | 12 |
| activity | trying new/different activities (4) | 12 |
| thing | “do a variety of things” (3) | 11 |
| job | (no pattern recognizable) | 10 |

Table **29**

***Brazil results of university sample for A varied life***

| Word | Meaning (Instantiation) | Absolute Frequency |
| --- | --- | --- |
| friend | "Friends and family going to new places". "Female friends enjoying" (4) | 31 |
| new | "New cultures". "New foods". "New friends" (5) | 21 |
| work | "Business traveling." "Working in several shifts" (4) | 18 |
| life | "Healthier life". " choosing new ways of living" (3) | 17 |
| family | "Family out of routine search through travel" (3) | 13 |
| know | "See the world". "Meet new people" (5) | 12 |
| day | "Travel on a daily basis with people." "Daily basis busy life routine" (4) | 12 |
| travel | "Traveling and seeing the world." "You travel your friends and family" (4) | 12 |
| search | "Attempts to make things." "Try to learn new traditions" (5) | 11 |
| familiar | "Practicing different sports with friends and family" (4) | 11 |
| work | "Coworkers, family and friends encouraging" (3) | 11 |
| different | "'Practice different sports." "Seeing different places" (4) | 10 |
| several | "Working at various jobs." "Work in various shifts" (4) | 10 |

Table 30

India results of university sample for A varied life

| Word | Meaning (Instantiation) | Absolute Frequency |
| --- | --- | --- |
| life | (no pattern recognizable) | 23 |
| friend | (no pattern recognizable) | 20 |
| family | (no pattern recognizable) | 16 |
| person | (no pattern recognizable) | 15 |

**Daring**


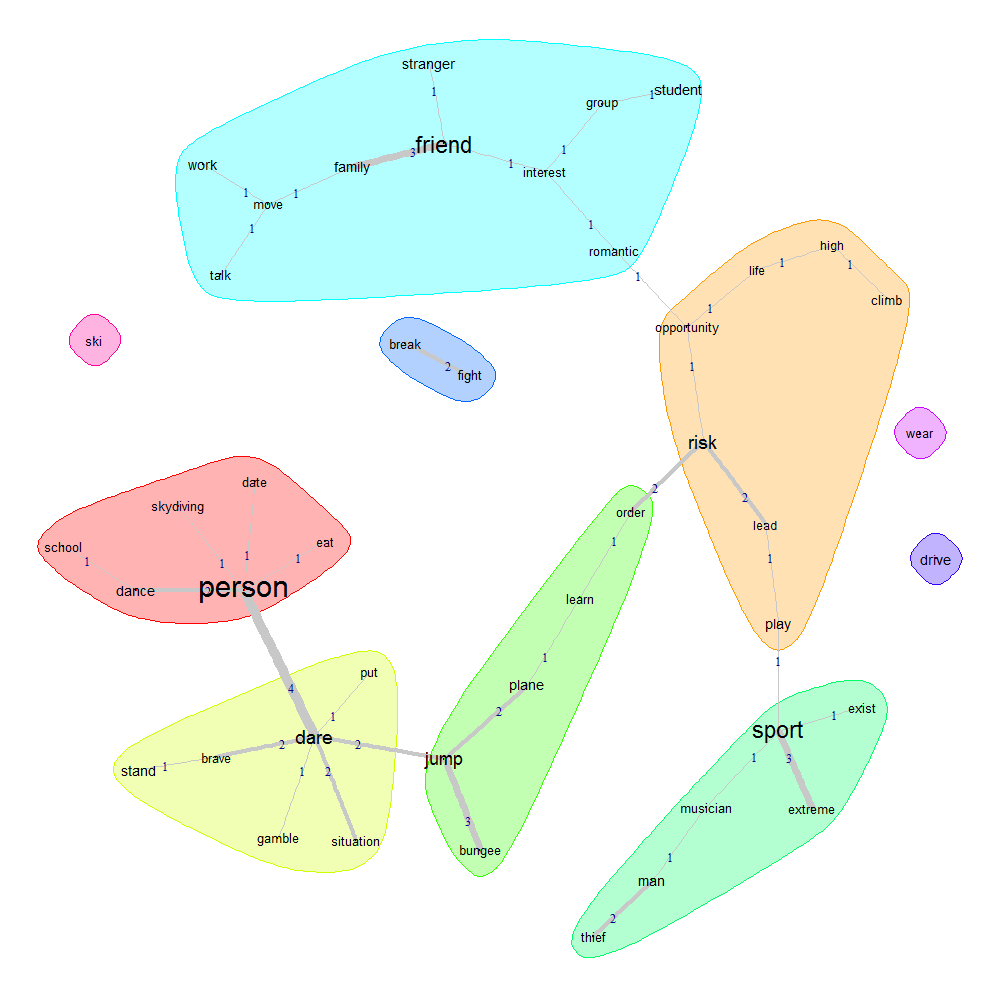


*Figure* ***10.*** Result of the graphical similarity analysis for ‘Daring’. Numbers indicate how often two words were mentioned together in single responses. Same colours indicate that words were mentioned together.

Table **31**

***UK results of university sample for Daring***

| Word | Meaning (Instantiation) | Absolute Frequency |
| --- | --- | --- |
| person | (no pattern recognizable) | 25 |
| sport | “extreme sports” (3) | 17 |
| friend | “friends” as relevant people (5) | 15 |
| dare | (no pattern recognizable) | 12 |
| risk | “have to take risks in order to try and win” (3) | 10 |

Table **32**

***Brazil results of university sample for Daring***

| Word | Meaning (Instantiation) | Absolute Frequency |
| --- | --- | --- |
| Studying | "taking a test without studying." "Leaving home to study in another city" | 10 |
|  |  |  |

Table 33

India results of university sample for Daring

| Word | Meaning (Instantiation) | Absolute Frequency |
| --- | --- | --- |
| dare | (no pattern recognizable) | 15 |
| person | (no pattern recognizable) | 15 |
| situation | (no pattern recognizable) | 14 |

**Pleasure**


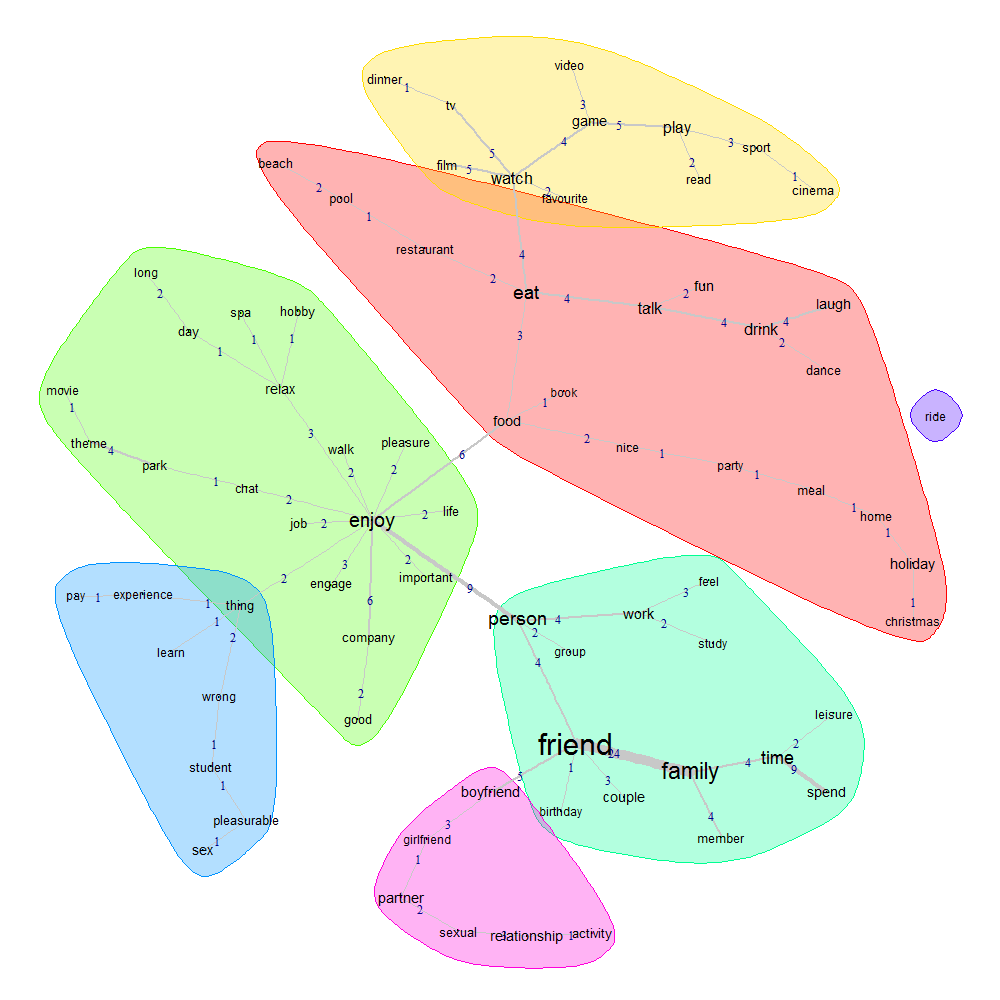


*Figure* ***11***. Result of the graphical similarity analysis for ‘Pleasure’. Numbers indicate how often two words were mentioned together in single responses. Same colours indicate that words were mentioned together.

Table **34**

***UK results of university sample for Pleasure***

| Word | Meaning (Instantiation) | Absolute Frequency |
| --- | --- | --- |
| friend | “spending time with family and friends”, “friends” as relevant people (4) | 59 |
| family | “spending time with family and friends”, “family” as relevant people (4) | 38 |
| enjoy | “Doing something you enjoy, such as a hobby”, “enjoy a fulfilling sexual relationship with their partner”, “enjoying food” (4) | 29 |
| person | “people” as relevant people (3) | 24 |
| eat | “enjoying food when eating”, “eating together” (5) | 24 |
| time | “spending time with family and friends”, “leisure time” (4) | 21 |
| watch | “watching a film” (4) | 17 |
| work | (no pattern recognizable) | 16 |
| talk | “talking” (5) | 14 |
| play | “playing games” (4) | 14 |
| drink | “drinking”, “having a drink” (4) | 14 |
| partner | “partner” as relevant people (5) [with whom you can have pleasure] | 13 |
| holiday | “on holiday” (4) | 12 |
| boyfriend | “spending time with my boyfriend” (5) | 11 |

Table **35**

***Brazil results of university sample for Pleasure***

| Word | Meaning (Instantiation) | Absolute Frequency |
| --- | --- | --- |
| friend | "Friends and family going to the movies." "Friends boyfriend playing video games" (4) | 65 |
| family | "Family and friends having fun." "Family and friends having fun playing football" (5) | 32 |
| be | "Being in the presence of nice people" (4) | 30 |
| pleasure | "Experiencing pleasure in the work." "Pleasurable feeling of satisfaction" (3) | 26 |
| boyfriend | "boy- or girlfriends and family traveling to see new places" (2) | 21 |
| day | "Going to the cafeteria every day." "relief the stress of everyday life" (4) | 16 |
| work | "Experiencing pleasure in the work." "Happy to be working together" (4) | 16 |
| eat | "Eating good food". "Friends and family eating in a bar" (4) | 14 |
| familiar | "To shop with friends and family." "Family Reunion" (3) | 14 |
| know | "Sightseeing", "Going to parks laughing with family" (4) [visiting, seeing, meeting, knowing have the same meaning in Portuguese in this context] | 13 |
| husband | "Husband, friend and family activities for practicing a healthier life" (4) | 13 |
| time | "Times of relaxation", "pleasant time" (4) | 12 |
| individual | "Individuals exercising" (3) | 11 |
| life | "Healthier life". "Living in family life" (4) | 11 |
| attend | "Watch a football game." "Watch a movie with a loved person" (3) | 10 |

Table 36

India results of university sample for Pleasure

| Word | Meaning (Instantiation) | Absolute Frequency |
| --- | --- | --- |
| friend | family and friends (3) | 18 |
| person | (no pattern recognizable) | 17 |
| happy | (no pattern recognizable) | 10 |

**Success**


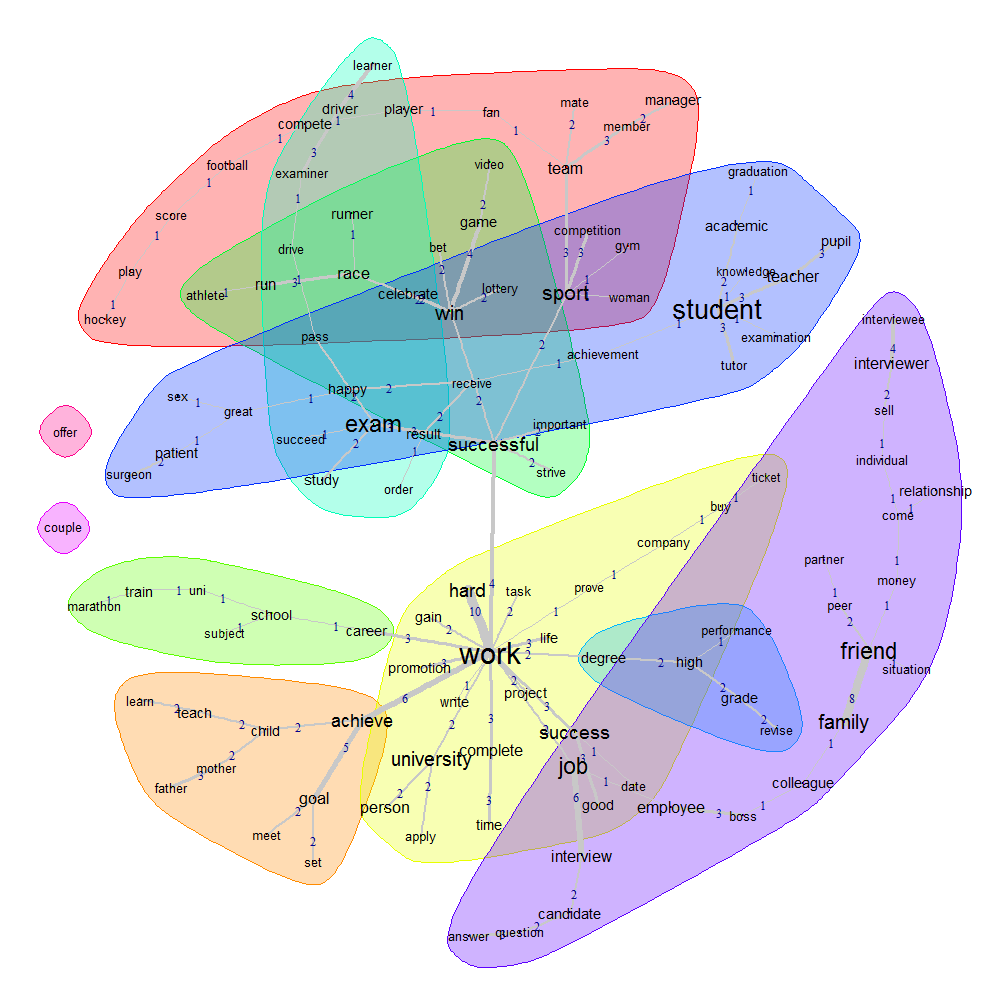


*Figure* ***12***. Result of the graphical similarity analysis for ‘Success’. Numbers indicate how often two words were mentioned together in single responses. Same colours indicate that words were mentioned together.

Table **37**

***UK results of university sample for Success***

| Word | Meaning (Instantiation) | Absolute Frequency |
| --- | --- | --- |
| work | “Team members work hard to win and be successful in their sport”, “working hard to meet deadlines and achieve goals” (3) | 33 |
| student | “student striving for top grades” , “students” as relevant people (4) | 28 |
| exam | ““passing your exams”, being good in exams (4) | 22 |
| job | “a job interview”, “in a job” (4) | 20 |
| friend | “friends” as relevant people (4) | 20 |
| win | winning in order to be successful, e.g. a lottery, a game (3) | 17 |
| sport | “sports competition”, “sports” as relevant situation (4) | 17 |
| university | “university” as a relevant place (4) | 16 |
| family | “successful home and family life”, “family” as relevant people (3) | 16 |
| hard | “studying hard to do well in exams”, “work hard in order to be successful” (4) | 14 |
| achieve | “achieve goals” (3) | 14 |
| successful | “aim to be successful” (4) | 13 |
| success | having success (4) | 13 |
| goal | “achieve goals” (3) | 12 |
| race | doing a race (2) | 11 |
| team | “Team members work hard to win and be successful” (mostly sport teams) (4) | 10 |
| person | (no pattern recognizable) | 10 |

Table **38**

***Brazil results of university sample for Success***

| Word | Meaning (Instantiation) | Absolute Frequency |
| --- | --- | --- |
| person | "People are approved in some selection" (5) [i.e. passing an entrance exam] | 41 |
| success | "Financial success". "Success in studies" (4) | 24 |
| get | "Able to meet objectives." (4) | 23 |
| friend | "Friends celebrating his professional career" victorious situations friends (5) [e.g. if you have passed an entrance exam] | 19 |
| business | "Private company" (3) | 15 |
| family | "Family get to have a good income." "Families having fun on the beach" (5) | 15 |
| life | "Overcome the obstacles of life" (4) | 15 |
| study | "Studying for an entrance exams and passing them" (4) | 14 |
| its | "His family". "Their goals" (3) | 14 |
| familiar | "Family congratulating the success" (3) | 12 |
| goal | "Life goals". "Personal goals" (4) | 12 |
| my | "My life". "My mother" (3) | 12 |
| pass | "Passing the entrance exams." (5) | 12 |
| professional | "Professional achievement". "Professional recognition" (4) | 12 |
| employment | "Get a job" (5) | 11 |
| achievement | "Personal fulfillment." "Professional achievement". "fullfilling of dreams" (5) | 11 |
| doctorate | "Finishing a doctorate" (5) | 10 |

Table 39

India results of university sample for Success

| Word | Meaning (Instantiation) | Absolute Frequency |
| --- | --- | --- |
| friend | family and other people (4) | 28 |
| person | (no pattern recognizable) | 28 |
| family | family and friends (3) | 21 |
| life | (no pattern recognizable) | 15 |
| success | (no pattern recognizable) | 15 |
| work | working hard (4) | 13 |
| hard | working or trying hard (5) | 12 |
| good | being good (3) | 11 |
| job | (no pattern recognizable) | 10 |

**Ambition**


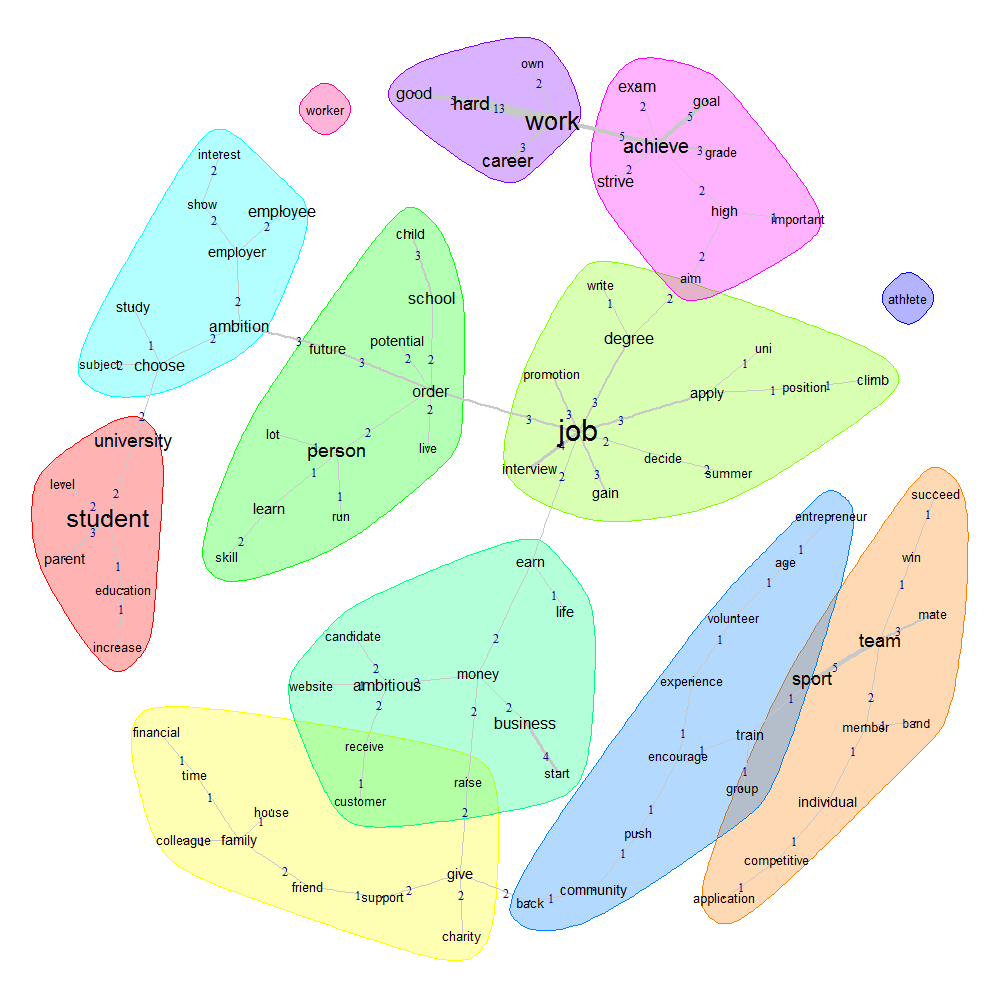


*Figure* ***13.*** Result of the graphical similarity analysis for ‘Ambition’. Numbers indicate how often two words were mentioned together in single responses. Same colours indicate that words were mentioned together.

Table **40**

***UK results of university sample for Ambition***

| Word | Meaning (Instantiation) | Absolute Frequency |
| --- | --- | --- |
| job | “applying for a new job”, “doing their job as best they can maybe to earn a promotion” (4) | 31 |
| work | “working to achieve their goal” (5) | 25 |
| student | “students” as relevant people (5) | 24 |
| achieve | “working to achieve their goal” (4) (similar to ‘success’ instantiation) | 15 |
| university | “university” as a relevant place (4) (similar to ‘success’ instantiation) | 13 |
| team | “Being in a competitive sports team” (3) (very similar to ‘success’ instantiation) | 13 |
| person | (no pattern recognizable) | 13 |
| hard | “working and training hard to achieve success” (4) (similar to ‘success’ instantiation) | 13 |
| sport | “sport” as relevant situation (4) | 12 |
| career | “career” as relevant situation (4) | 12 |
| school | “attending school and working hard to achieve good grades” (4) | 10 |

Table **41**

***Brazil results of university sample for Ambition***

| Word | Meaning (Instantiation) | Absolute Frequency |
| --- | --- | --- |
| family | "Having children, friends and family" wish to build a Family (4) | 14 |
| want | "Want it all". "Want sth. what you don’t have" (4) | 14 |
| get | "Get the top job." "Get a raise" Achieve professional success (5) | 13 |
| other | "Always passing in front of the other" (3) [i.e. in order to pass entrance exams] | 13 |
| post | "Get the top job", "office" (4) [same like “get”] | 10 |
| colleague | "Colleague being jealous" (4) | 10 |
| society | "Competitive society". "Capitalist society" (4) | 10 |

Table 42

India results of university sample for Ambition

| Word | Meaning (Instantiation) | Absolute Frequency |
| --- | --- | --- |
| job | Getting a good job (4) | 18 |
| education | (no pattern recognizable) | 17 |
| good | good job (3), good education (2) | 17 |
| person | (no pattern recognizable) | 17 |
| work | working hard (4) | 14 |
| ambition | (no pattern recognizable) | 11 |
| friend | friends and family (2) | 11 |
| hard | working hard (5) | 11 |

**Wealth**


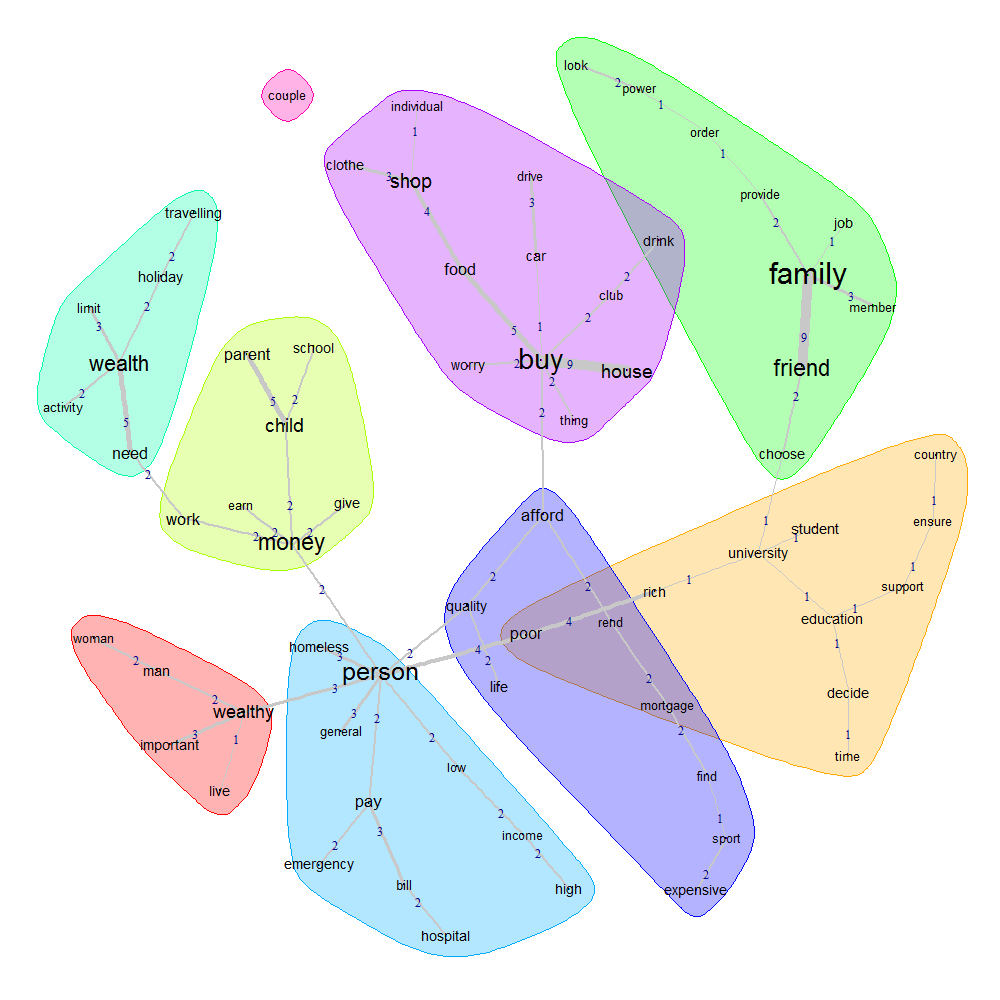


*Figure* ***14***. Result of the graphical similarity analysis for ‘Wealth’. Numbers indicate how often two words were mentioned together in single responses. Same colours indicate that words were mentioned together.

Table **43**

***UK results of university sample for Wealth***

| Word | Meaning (Instantiation) | Absolute Frequency |
| --- | --- | --- |
| family | “providing for a family”, “family” as relevant people (3) | 26 |
| buy | “buying a house” (3) | 24 |
| person | (no pattern recognizable) | 23 |
| wealth | “those with more wealth have greater choice in this” (3) | 17 |
| money | (no pattern recognizable) | 16 |
| friend | “friends” as relevant people (4) | 16 |
| child | “provide children with what they need” (4) | 13 |
| shop | “going shopping”, food and clothes (5) | 12 |
| house | “buying a house” (4) | 11 |
| wealthy | (no pattern recognizable) | 10 |
| food | “buying food” (3) | 10 |
| afford | being able to afford expensive things (4) | 10 |

Table **44**

***Brazil results of university sample for Wealth***

| Word | Meaning (Instantiation) | Absolute Frequency |
| --- | --- | --- |
| friend | "Family and friends traveling" (5) | 33 |
| family | "Family friends have a healthy life." "Family living comfortable" (5) | 31 |
| wealth | "Family since greater wealth that does not exist" attaches great Importance to interpersonal relationships (4) [i.e. the most important thing what you can have is a family] | 12 |
| familiar | "Family environment". "Family members caring" (4) | 10 |
| health | "Taking care of health" (5) | 10 |
| life | "Celebrating Life". "Improving the quality of life" (3) | 10 |

Table 45

*India results of university sample for Wealth*

| Word | Meaning (Instantiation) | Absolute Frequency |
| --- | --- | --- |
| family | (no pattern recognizable) | 22 |
| friend | (no pattern recognizable) | 18 |
| money | (no pattern recognizable) | 16 |
| wealth | (no pattern recognizable) | 15 |
| person | (no pattern recognizable) | 12 |
| life | living a good/comfortable life (4) | 11 |

**Social Power**


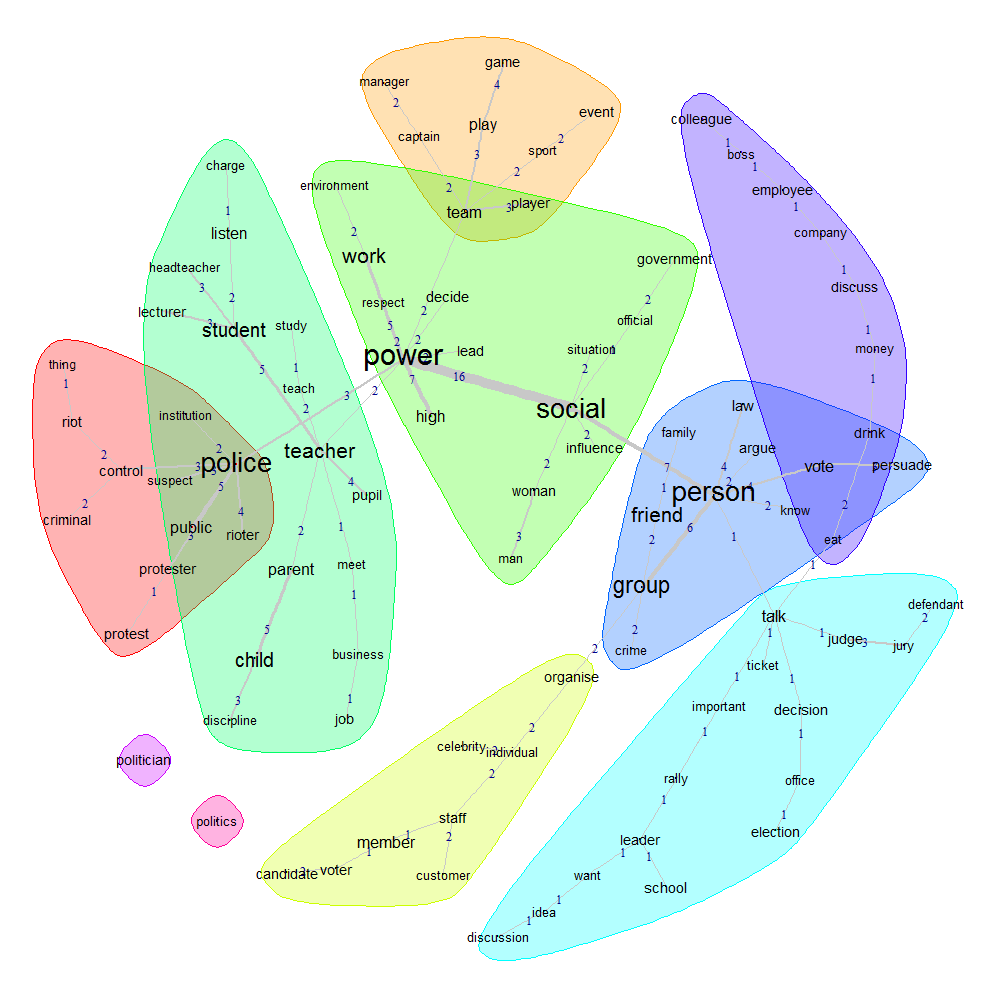


*Figure* ***15*.** Result of the graphical similarity analysis for ‘Social Power’. Numbers indicate how often two words were mentioned together in single responses. Same colours indicate that words were mentioned together.

Table **46**

***UK results of university sample for Social Power***

| Word | Meaning (Instantiation) | Absolute Frequency |
| --- | --- | --- |
| person | (no pattern recognizable) | 27 |
| power | “social power”, (2) | 25 |
| social | “having good social power will help greatly”, “The person with the highest social power is the person who decides” (3) | 23 |
| police | “police” as relevant people with social power (4) | 23 |
| group | “group” as relevant people (3) | 17 |
| friend | “friends” as relevant people (4) | 16 |
| work | (no pattern recognizable) | 14 |
| teacher | “teachers” as relevant people with social power (4) | 14 |
| vote | “trying to persuade people to vote for them” (5) | 12 |
| student | “teacher has power to teach students”, “students as relevant people (4) | 12 |
| child | “parents trying to discipline children” (3) | 12 |

Table **47**

***Brazil results of university sample for Social Power***

| Word | Meaning (Instantiation) | Absolute Frequency |
| --- | --- | --- |
| society | "Welfare of society". "Democratic society" related to social situations common (5) [General well-being of society] | 23 |
| child | "Children and adolescents promoting sports." "School feeding and housing of children" (3) | 13 |
| family | "Social workers helping families' situations In which families receive help (4) | 13 |
| right | "Right of citizens" (5) | 12 |
| improvement | "Improvements to the population"; "Improvements to the community" (4) | 12 |
| community | "Community, family and friends" (5) | 11 |
| government | "Government that helps in feeding" (5) | 10 |

Table 48

India results of university sample for Social Power

| Word | Meaning (Instantiation) | Absolute Frequency |
| --- | --- | --- |
| person | (no pattern recognizable) | 37 |
| give | give rights to people (12) | 12 |
| social | social power (3) | 12 |
| girl | (no pattern recognizable) | 11 |
| work | (no pattern recognizable) | 10 |

**Family Security**


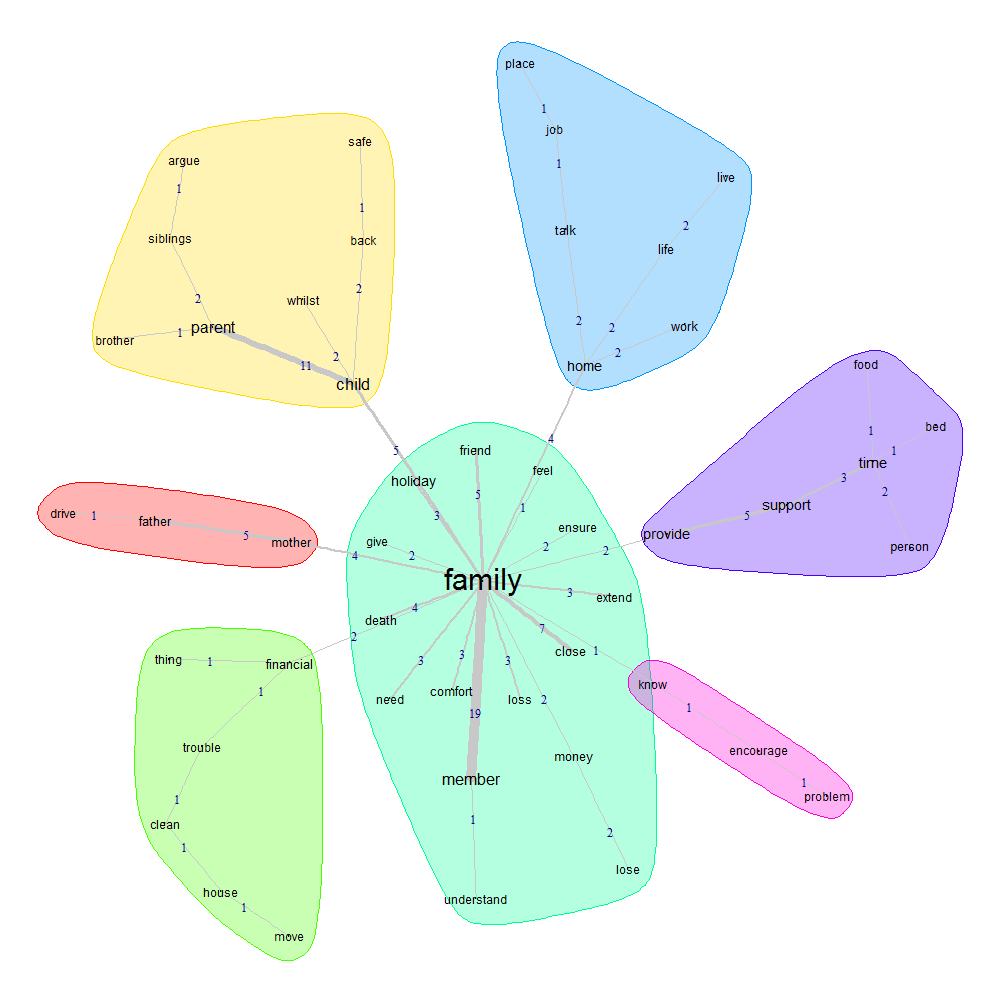


*Figure* ***16*.** Result of the graphical similarity analysis for ‘Family security’. Numbers indicate how often two words were mentioned together in single responses. Same colours indicate that words were mentioned together.

Table **49**

***UK results of university sample for Family Security***

| Word | Meaning (Instantiation) | Absolute Frequency |
| --- | --- | --- |
| family | “comforting other family members” (including financial support), “family” as relevant people (4) | 87 |
| child | “keeping watch of the children, making sure they do not run off etc.” staying in contact with children (4) | 24 |
| member | “family members” as relevant people (4) | 20 |
| parent | “children/ parents” as relevant people (4) | 18 |
| time | “supporting each other during hard times” (2) | 14 |
| support | “providing support and advice, being there no matter what, sticking together”, “supporting you if you get into difficulties, providing a stable home” (5) | 14 |
| provide | provide support/reassurance/help | 13 |
| home | “providing a stable home life” (3) | 10 |
| holiday | “holiday” as a relevant situation (5) | 10 |

Table **50**

***Brazil results of university sample for Family Security***

| Word | Meaning (Instantiation) | Freq. |
| --- | --- | --- |
| son | "Dad took son to play in the park." "Safety at home for parents and children" (3) | 37 |
| father | "Physical presence of parents" (3) | 36 |
| mother | "Father and mother come home" (3) | 35 |
| family | "Leisure on the beach with the whole family." "Taking care of all the family" (4) | 32 |
| all | "The whole family strolling carriage" (3) | 22 |
| security | "Electronic security". "Needing more security and police at the streets" (4) | 20 |
| home | "Installed electric fences in the wall of your home to feel safer" (3) | 18 |
| brother | "health insurance for me, mother, father, sister, and nephew." "Support mother and brother" (2) | 16 |
| familiar | "Contribute to the family income." "Mother and Father ensuring family financial situation" (4) | 15 |
| your | "Pay your taxes". Woman denounces her husband for domestic violence "(2) | 15 |
| your | "Having the risk of your home being burgled." " violence in public transport in your city" (2) | 11 |

Table 51

India results of university sample for Family Security

| Word | Meaning (Instantiation) | Absolute Frequency |
| --- | --- | --- |
| family | (no pattern recognizable) | 51 |
| security | security of one’s family (5) | 14 |
| life | security of one’s life (4) | 13 |
| member | family member (5) | 13 |
| person | (no pattern recognizable) | 12 |
| child | (no pattern recognizable) | 11 |
| care | caring about others (3), not caring about others (3) | 10 |

**Respect for Tradition**


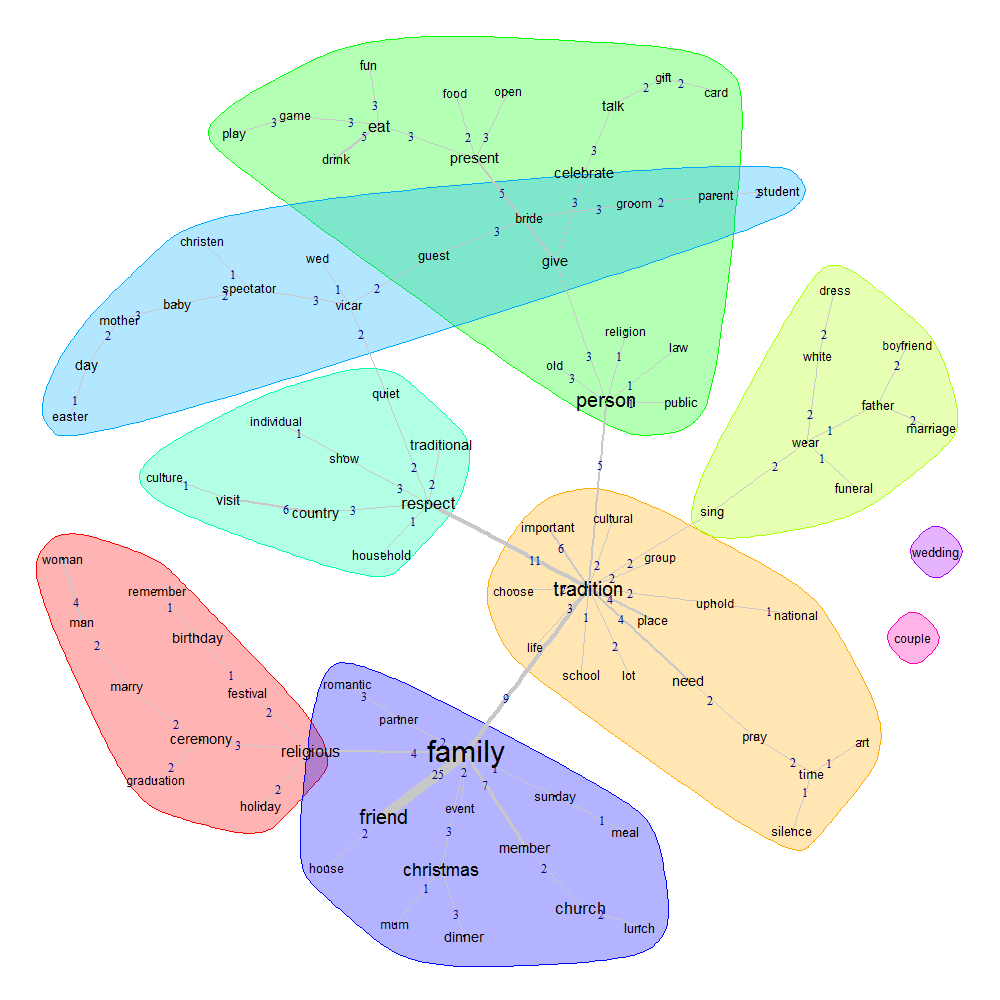


*Figure* ***17.*** Result of the graphical similarity analysis for ‘Respect for tradition’. Numbers indicate how often two words were mentioned together in single responses. Same colours indicate that words were mentioned together.

Table **52**

***UK results of university sample for Respect for Tradition***

| Word | Meaning (Instantiation) | Absolute Frequency |
| --- | --- | --- |
| family | “family” as relevant people (4) | 68 |
| tradition | respecting/understanding traditions (3) | 35 |
| person | “persons” as relevant people who follow traditions (4) | 33 |
| friend | “friends” as relevant people (5) | 30 |
| Christmas | Having Christmas dinner with family, spending time with family during Christmas (5) | 24 |
| respect | “respecting the traditions” in various situations (4) | 20 |
| church | “church” as a relevant place (5) | 19 |
| religious | “religious ceremonies” (3) | 16 |
| eat | “Eating Christmas lunch together”, “Eating, drinking, helping to clear up together, swapping news” (3) | 16 |
| celebrate | Celebrating Christmas, marriage, birthday… (4) | 13 |
| present | giving presents for Christmas or birthday (4) | 12 |
| day | fathers/mother/valentines etc. day (4) | 11 |
| ceremony | Religious and graduation ceremonies (4) | 10 |
| birthday | “birthday” as relevant situation (5) | 10 |

Table **53**

***Brazil results of university sample for Respect for Tradition***

| Word | Meaning (Instantiation) | Freq. |
| --- | --- | --- |
| family | "Celebrating festive days new, Christmas year, St. John's, Carnival, Easter lunch with family". "Family celebrating an event" (4) | 32 |
| father | "Respect the request of the parents." "respect the parents" (5) | 26 |
| son | "Presence of children in cults or services." "Son hear teachings of his father" (4) | 23 |
| your | "Respecting others' opinions regardless of their culture" (3) | 18 |
| tradition | "Keeping alive the traditions." "Annually celebrate something that considers a family tradition" (4) | 18 |
| respect | "Respect your elders". "respect the parents" (4) | 17 |
| all | "Following the tradition of all the men of the family must graduate" (3) | 16 |
| friend | "Family and friends having fun." "Respect for religious beliefs neighbors friends" (4) | 14 |
| respect | "Respect for the family." "Respect the holy holidays." (the ‘Saint holidays’), "Respect for tradition" (5) | 13 |
| familiar | "Family usually having lunch." "Ethical and respectful family" (4) | 12 |
| festival | "Religious festivals". "Cultural feast". "June festivals" (5) | 11 |
| follow | "Members of society follow certain dressing code." "Follow the traditions of their grandparents" (4) | 11 |
| city | "Show the tradition of his city." "Anniversary celebration of the city" (4) | 10 |

Table 54

India results of university sample for Respect for Tradition

| Word | Meaning (Instantiation) | Absolute Frequency |
| --- | --- | --- |
| tradition | (no pattern recognizable) | 26 |
| person | (no pattern recognizable) | 23 |
| family | (no pattern recognizable) | 18 |
| respect | (no pattern recognizable) | 17 |
| wear | wearing traditional dresses (5) | 16 |
| traditional | traditional dresses (4) | 15 |
| dresses | wearing traditional dresses (5) | 13 |
| festival | (no pattern recognizable) | 13 |
| friend | (no pattern recognizable) | 13 |
| culture | (no pattern recognizable) | 10 |

**Self-discipline**


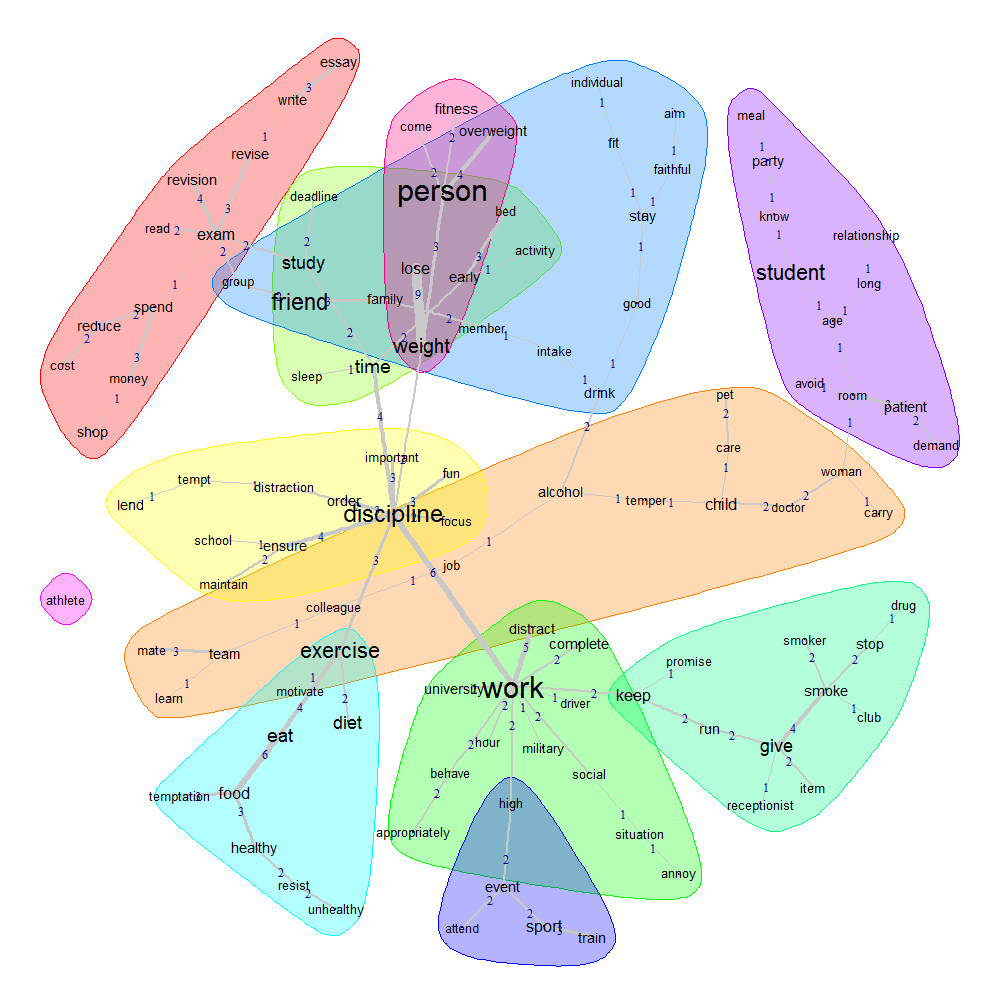


*Figure* ***18***. Result of the graphical similarity analysis for ‘Self-discipline’. Numbers indicate how often two words were mentioned together in single responses. Same colours indicate that words were mentioned together.

Table **55**

***UK results of university sample for Self-discipline***

| Word | Meaning (Instantiation) | Absolute Frequency |
| --- | --- | --- |
| work | “get work done trying your best to succeed” (3) | 34 |
| person | “people” as relevant people, who are or should be self-disciplined (4) | 34 |
| discipline | “need to discipline yourself” (3) | 24 |
| friend | “friends” as relevant people (5) | 22 |
| student | “students” as relevant people (5) | 19 |
| exercise | “exercising” (4) | 19 |
| weight | “losing weight” (5) | 17 |
| time | (no pattern recognizable) | 15 |
| eat | “Not eating all of the food”, “Resisting the urge to eat unhealthy food” (5) | 15 |
| give | “Giving up smoking”, “Have to keep motivating yourself to run even if you want to give up” (3) | 14 |
| diet | “dieting” (5) | 13 |
| study | “studying” (5) | 12 |
| sport | training/playing sport (4) | 11 |
| exam | “exams” as relevant situations (4) | 11 |
| lose | “losing weight” (5) | 10 |
| keep | keeping yourself motivated/healthy (4) | 10 |

Table **56**

***Brazil results of university sample for Self-discipline***

| Word | Meaning (Instantiation) | Freq. |
| --- | --- | --- |
| your | "Succeed in your professional career." "Have discipline in your work routine " (4) | 23 |
| all | "Do all the work." "Every athlete needs discipline." "Every society needs a lot of self-discipline" (4) | 14 |
| day | "Control in a situation of everyday life" (3) | 12 |
| teacher | "Teachers teaching a new language." "Teachers and students studying" (4) | 12 |
| friend | "Family leisure family friends in the park" (2) | 11 |
| student | "Student does not respect the teachers." "Students completing the course" (4) | 10 |
| family | "My family supports me and encourages me to be better qualified at work" (3) | 10 |

Table 57

India results of university sample for Self-discipline

| Word | Meaning (Instantiation) | Absolute Frequency |
| --- | --- | --- |
| discipline | “self-discipline” (5), “should maintain self-discipline” (3) | 33 |
| person | “people” as relevant people, who are or should be self-disciplined (5) | 22 |
| friend | “friends” as relevant people (5) | 16 |
| work | “work” as typical activity (4), “work” place (4) | 16 |
| student | “students” as relevant people (5) | 13 |
| family | “family” as relevant people (5) | 11 |

**Obedience**


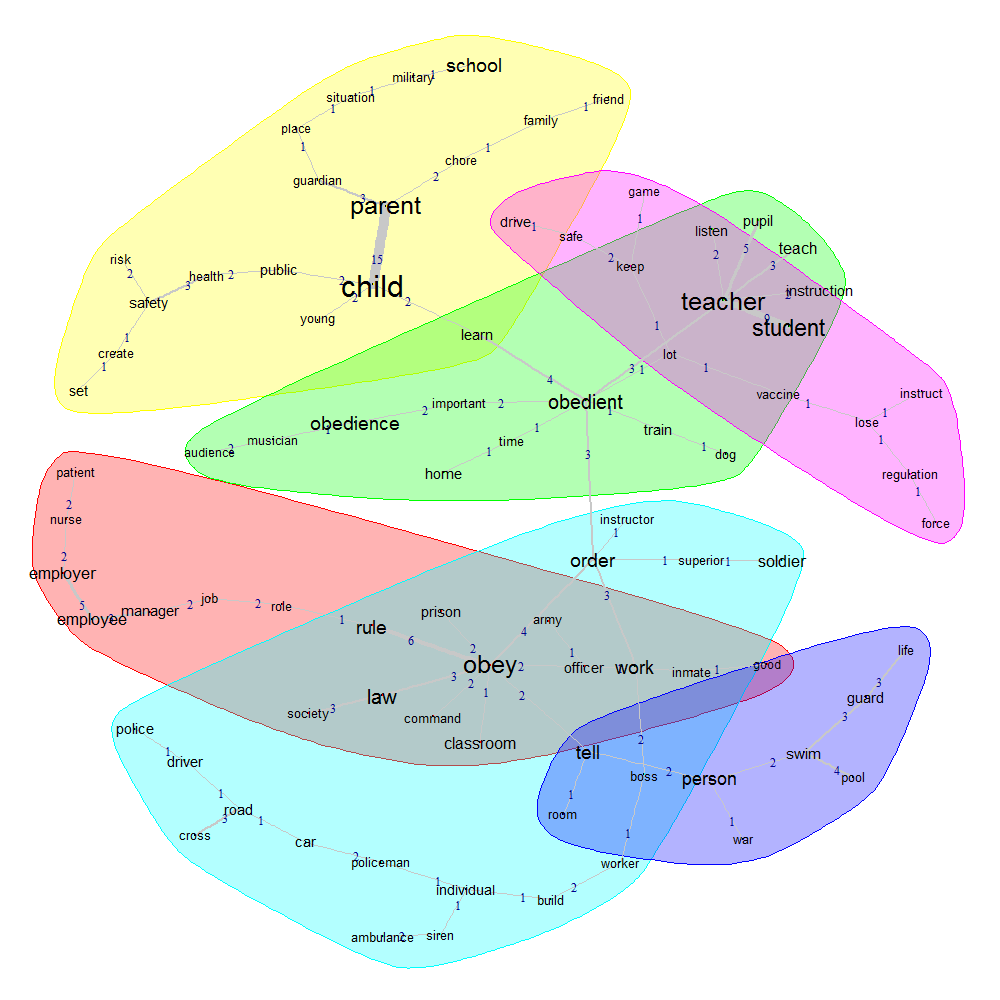


*Figure* ***19***. Result of the graphical similarity analysis for ‘Obedience’. Numbers indicate how often two words were mentioned together in single responses. Same colours indicate that words were mentioned together.

Table **58**

***UK results of university sample for Obedience***

| Word | Meaning (Instantiation) | Absolute Frequency |
| --- | --- | --- |
| child | “Children need to be obedient” (4) | 31 |
| obey | “obey the law”, “obeying rules “ (3) | 23 |
| teacher | “obey teachers” (4) | 22 |
| parent | “parents” as relevant people, to whom you have to be obedient (4) | 22 |
| student | “students” as relevant people, who have to be obedient (4) | 21 |
| order | obeying orders (5) | 15 |
| obedient | “attending school, being obedient as required by teachers”, “you [should] be obedient, you would learn and experience more than less obedient interns” (4) | 15 |
| obedience | (no pattern recognizable) | 15 |
| law | “Compliance with the law”, “obey the law” (5) | 14 |
| work | “at work” as relevant situation (4) | 13 |
| tell | High-ranking person tells inferiors what to do (4) | 13 |
| school | “attending school, being obedient as required by teachers” (5) | 13 |
| rule | obeying/following rules (4) | 12 |
| person | (no pattern recognizable) | 12 |
| employee | “employees” as relevant people, who have to be obedient (4) | 10 |

Table **59**

***Brazil results of university sample for Obedience***

| Word | Meaning (Instantiation) | Freq. |
| --- | --- | --- |
| father | "Obey the parents." "listening to your parents" (4) | 49 |
| obey | "Obey parents even without agreeing." "Obey the seniors." "Obey the rules" (5) | 43 |
| son | "Son or daughter compliance to rules." "Apologize father and SON" (4) | 28 |
| teacher | "Respect to the professor." "Classroom teacher and students who learn" (4) | 22 |
| student | "Students who learn." "Students who respect staff members" (4) | 21 |
| ask | "Parent and father asking to do things". "Professor asks for silence" (5) | 20 |
| rule | "Accept boss’ rule." "Employees and companies following rules" (4) | 20 |
| follow | "Follow the rules imposed by government." "Following the instructions" (4) | 19 |
| mother | " obey mother and father." "help your mother when she asks" (4) | 14 |
| old | "listen to older people." "take advices from older people". "be obedient to older people" (5) | 14 |
| friend | "Having respect for a friend" (3) | 12 |
| respect | "Obeying and respecting older people". "respect older people." "Respect for parents" (5) | 12 |
| class | "Obedience in classroom." "Attend to classes and obey the rules" (4) | 11 |
| law | "Follow the law". “ obey the traffic law." "Obey the laws of the government" (5) | 10 |

Table 60

India results of university sample for Obedience

| Word | Meaning (Instantiation) | Absolute Frequency |
| --- | --- | --- |
| work | “at work” (4) | 19 |
| teacher | “teacher” as typical people to whom one need to obey | 16 |
| family | (no pattern recognizable) | 14 |
| obedience/obedient | (no pattern recognizable) | 14 |
| student | “student must be obedient” (2) | 14 |
| person | (no pattern recognizable) | 10 |

**Helpfulness**


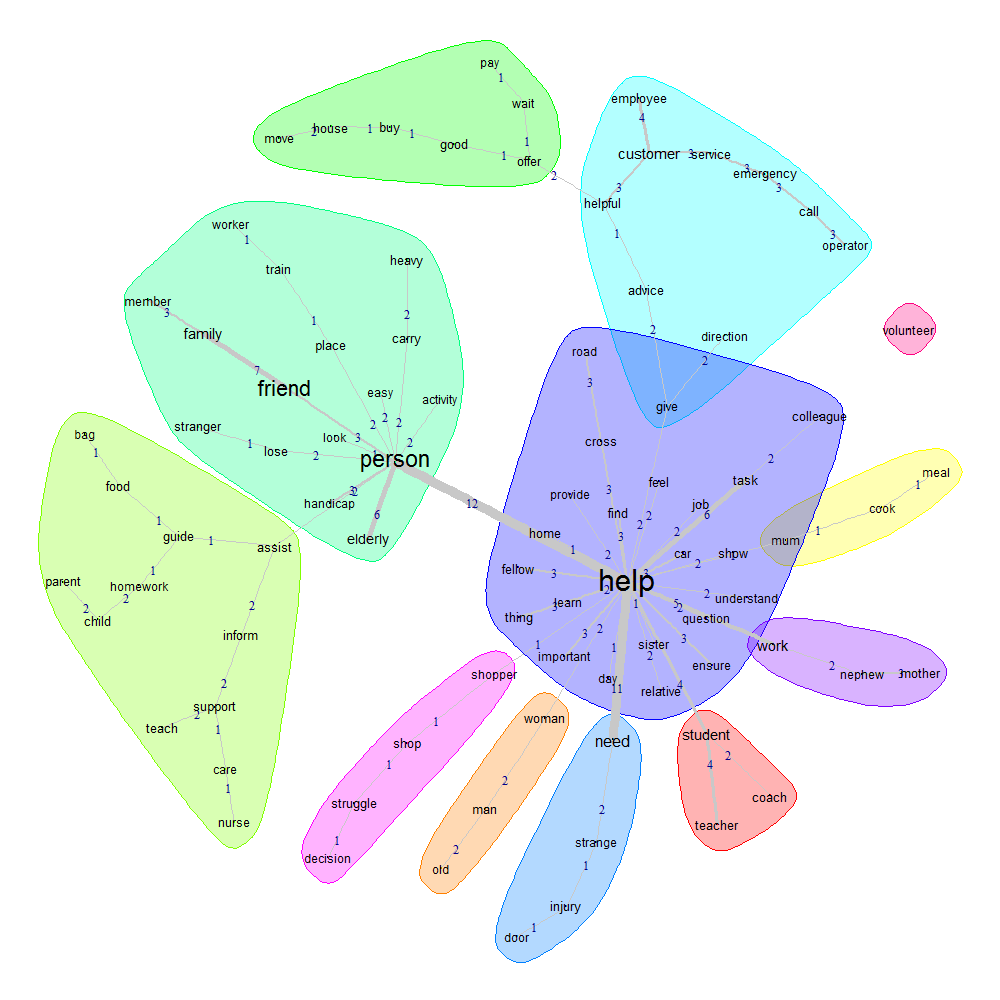


*Figure* ***20*.** Result of the graphical similarity analysis for ‘Helpfulness’. Numbers indicate how often two words were mentioned together in single responses. Same colours indicate that words were mentioned together.

Table **61**

***UK results of university sample for Helpfulness***

| Word | Meaning (Instantiation) | Absolute Frequency |
| --- | --- | --- |
| help | helping other people (4) | 67 |
| person | helping (elderly) people (3) | 45 |
| friend | “friends” as relevant people (5) | 37 |
| need | helping someone in need (3) | 17 |
| work | “helping with work situations” (2) | 13 |
| student | “students” as relevant people (4) | 12 |
| family | “family” as relevant people (4) | 12 |
| customer | “customer service” (4) | 11 |

Table **62**

***Brazil results of university sample for Helpfulness***

| Word | Meaning (Instantiation) | Freq. |
| --- | --- | --- |
| help | "Helping friends and family." "Help in difficult moments parents friends and family" (4) | 45 |
| friend | "Hear a familiar friend when needed" (4) | 41 |
| work | "Help work colleagues". "Offer help to another coworker" (4) | 40 |
| colleague | "Colleague offer help to another colleague." "Help the ‘next one’, neighbor or co-worker" (5) | 24 |
| elderly | "Helping an elderly cross the street." "Gives rise to an elderly passenger who has no where to sit" (5) | 18 |
| street | "Crossing the street elder or child." "Help a blind man cross the street" (5) | 17 |
| help | "Help poor people." "Help by giving food." "Helping people in poverty" (4) | 15 |
| give | "Gives a lift to another." "Giving another people place to sit" (4) | 15 |
| family | "Help needy families." "Understand the demands of other families" (4) | 15 |
| offer | "Help support". "Offer a ride." "Offer to help your mother" (5) | 15 |
| student | "College students are helping their colleagues" (3) | 14 |
| familiar | "Help friend and family when they are moving." "Pay a bill of a family" (4) | 14 |
| service | "Provide the requested service." "Performing free services" (4) | 13 |
| passenger | "A passenger offer your place to another" (4) | 12 |
| always | "Always present on the job." "In the leisure area is always helpful" (4) | 11 |
| ask | "Family and friends need help and ask for it" "A friend asks you to solve a problem" (4) | 10 |
| specify | "Families need and ask for help." "One needs a ride." "Poor people need of food" (4) | 10 |

Table 63

India results of university sample for Helpfulness

| Word | Meaning (Instantiation) | Absolute Frequency |
| --- | --- | --- |
| person | “person” as relevant people who (should) give or receive help (3) | 21 |
| friend | (no pattern recognizable) | 14 |

**Loyalty**


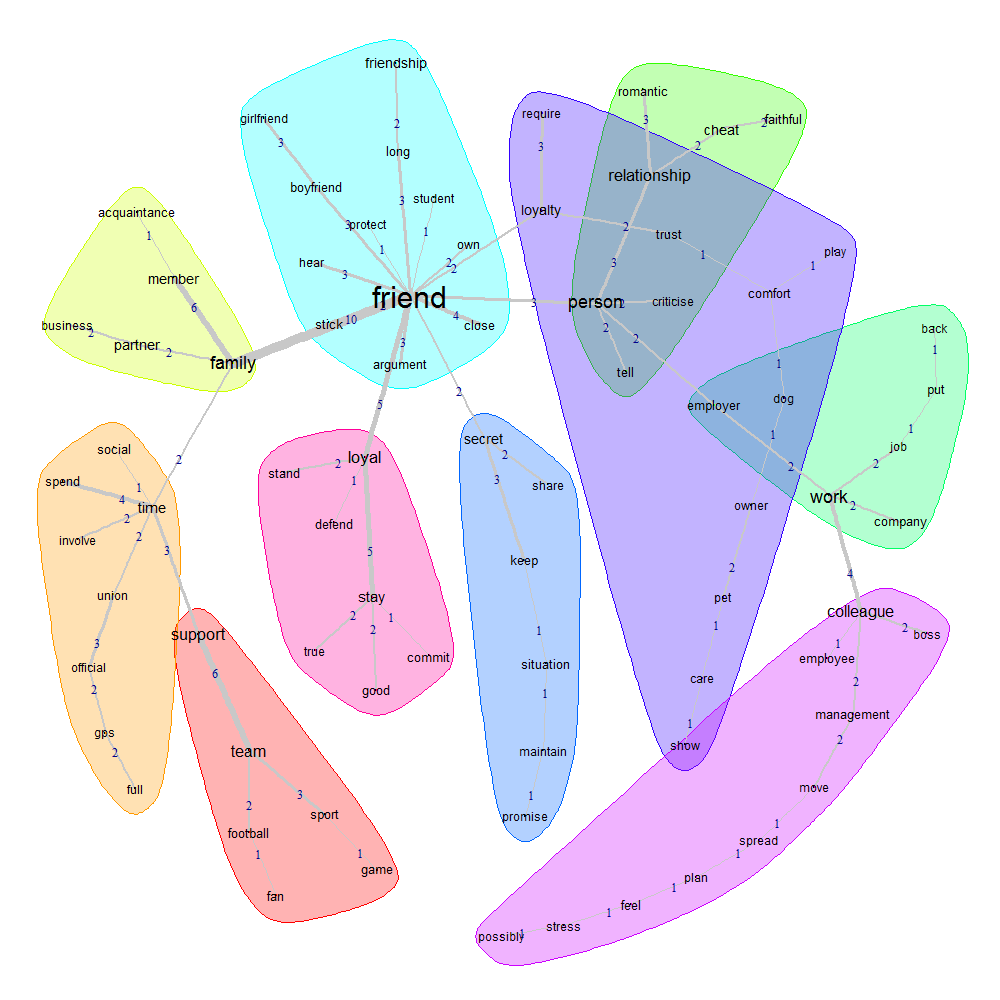


*Figure* ***21***. Result of the graphical similarity analysis for ‘Loyalty’. Numbers indicate how often two words were mentioned together in single responses. Same colours indicate that words were mentioned together.

Table **64**

***UK results of university sample for Loyalty***

| Word | Meaning (Instantiation) | Absolute Frequency |
| --- | --- | --- |
| friend | “standing by your friends and being loyal to them”, “friends” as relevant people (4) | 64 |
| family | “family” as relevant people, being loyal towards your family (3) | 23 |
| work | “in a work place” (4) | 22 |
| person | (no pattern recognizable) | 22 |
| relationship | “in a relationship” (5) | 19 |
| support | “Supporting your team at football, rugby”, “supporting each other” (e.g. family, friends) (4) | 18 |
| loyal | “A stranger shows romantic interest in you, but you stay loyal to your boyfriend, girlfriend”, “standing by your friends and being loyal to them” (4) | 17 |
| team | “Staying Loyal to team by no underhand tactics and by good teamwork” (4) | 15 |
| colleague | “defending a colleague” (2) | 15 |
| time | spending time with close ones if they are in trouble (2) | 13 |
| partner | “partner” as relevant people (4) | 13 |
| stay | “staying loyal” (5) | 12 |
| secret | “keeping secrets” (4) | 10 |

Table **65**

***Brazil results of university sample for Loyalty***

| Word | Meaning (Instantiation) | Freq. |
| --- | --- | --- |
| friend | "Do not lie to friend." "Friends who help in the crisis" (4) | 72 |
| loyalty | "In every marriage there must be loyalty." "Have loyalty to your boss." (4) | 25 |
| familiar | "Family always be faithful." "Help friends and family" (3) | 23 |
| fair | "Relationship shows a couple loyal to another." "Be loyal to those we love." "Always be loyal and true to who is on our side" (4) | 20 |
| business | "Demonstrate loyalty to the company that trusted him." "Have commitment to the company" (4) | 17 |
| always | "Always attend the same company." "Always be faithful and loyal to those who are always on our side" (4) | 15 |
| all | "Come together at all times" (3) | 15 |
| help | "Friends who help in the crisis." "Help a needy person on the street." "Is always willing to help" (5) | 14 |
| colleague | "Co-workers being fair" (3) | 13 |
| friendship | "The person is loyal to a friendship." "Friendship two friends keeping in touch whenever possible" (4) | 12 |
| brother | "When a brother helps his other brother" (3) | 12 |
| dog | "Dog shows loyalty to his master" (5) | 11 |
| husband | "Husband respecting his wife." "Husband and wife being loyal to the commitment of marriage" (4) | 11 |
| woman | "Marriage husband and wife." "" Woman is being faithful to her husband "(4) | 11 |
| boyfriend | "The girlfriend of a friend is flirting with me" (3) | 11 |
| money | "Working with money." "You lend money to friends" (4) | 10 |
| employee | "Loyalty of an employee in the company" (5) | 10 |

Table 66

India results of university sample for Loyalty

| Word | Meaning (Instantiation) | Absolute Frequency |
| --- | --- | --- |
| person | (no pattern recognizable) | 12 |
| loyal | (no pattern recognizable) | 10 |

**Honesty**


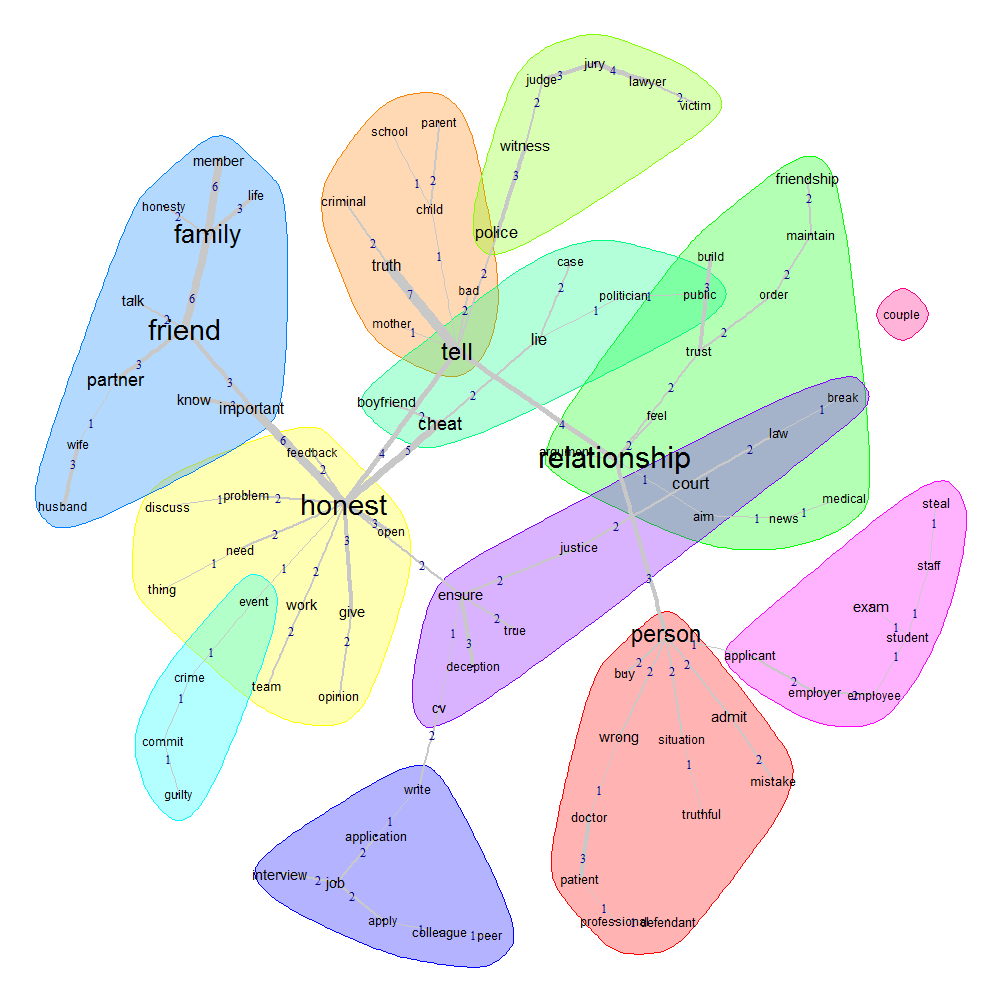


*Figure* ***22***. Result of the graphical similarity analysis for ‘Honesty’. Numbers indicate how often two words were mentioned together in single responses. Same colours indicate that words were mentioned together.

Table **67**

***UK results of university sample for Honesty***

| Word | Meaning (Instantiation) | Absolute Frequency |
| --- | --- | --- |
| relationship | “in a relationship” (4) | 31 |
| honest | “being honest” to friends, family etc. (4) | 30 |
| friend | “friends” as relevant people (5) | 30 |
| family | “family” as relevant people (4) | 25 |
| person | (no pattern recognizable) | 22 |
| tell | telling the truth (4) | 21 |
| partner | “partner” as relevant people (5) | 13 |
| cheat | “not cheating” (4) | 13 |
| lie | do not lie (4) | 12 |
| police | (no pattern recognizable) | 11 |
| court | “court” as relevant situation (5) | 11 |

Table **68**

***Brazil results of university sample for Honesty***

| Word | Meaning (Instantiation) | Freq. |
| --- | --- | --- |
| money | "Politicians stealing government money." "Someone finds money on the ground and returns." "Returns the money he received" (4) | 21 |
| find | "Find money". "Find documents and deliver the right person" (4) | 15 |
| return | "Get a wrong change and return." "Do not keep the money for themselves returning to the owner" (5) | 15 |
| other | "Be true to each other no lies." "Friends are honest with each other" (4) | 15 |
| change | "Get a wrong change and return" (5) | 15 |
| friend | "I am honest to friends." "Borrow money and give it back" (5) | 14 |
| receive | "Get a wrong change and return." "Return the money he received wrong" (5) | 13 |
| its | "Perceiving and understanding comprising its existence" (3) | 13 |
| honest | "An honest person". "My mother she is an example of honest person." "An honest person does not accept what is not yours" (4) | 12 |
| wrong | "Does not accept wrong change." "Get a wrong change and return" (5) | 10 |

Table 69

India results of university sample for Honesty

| Word | Meaning (Instantiation) | Absolute Frequency |
| --- | --- | --- |
| person | “person” as typical people who should be honest (3) | 34 |
| friend | (no pattern recognizable) | 25 |
| situation | (no pattern recognizable) | 18 |
| honest | “should be honest” (4) | 16 |
| family | (no pattern recognizable) | 12 |
| parent | “parents” as typical people | 11 |
| time | (no pattern recognizable) | 11 |
| respect | Being honest is to show respect to other people (5) | 10 |
| work | To “work” as a typical activity for which honesty is important | 10 |

**Detailed Results of Study 2**

Table S70

*Study 2*: Absolute frequencies of how often an instantiation thought to be related with various values (Brazil – UK)

|  | Brazil | | | | | | | United Kingdom | | | | | | |
| --- | --- | --- | --- | --- | --- | --- | --- | --- | --- | --- | --- | --- | --- | --- |
| **Unity with Nature** | Varied | Equality | Creativity | Wisdom | Loyalty | Unity | ? | Varied | Equality | Creativity | Wisdom | Loyalty | Unity | ? |
| Walking outside (1u1) | 13 | 4 | 1 | 2 | 3 | **47** |  | 2 |  | 2 |  |  | **39** |  |
| Being in the garden (1u2) | 4 |  | 3 |  | 2 | **57** | 1 |  |  |  |  |  | **40** |  |
| Preserving the environment (1b1) |  |  | 4 | 24 | 1 | **43** |  |  |  |  |  |  | **42** |  |
| Planting trees (1b2) |  | 1 |  | 9 |  | **58** |  |  |  | 1 |  |  | **43** |  |
| Walking on the beach (1b3) | 17 | 3 | 1 | 1 | 1 | **53** | 1 | 3 |  |  |  |  | **37** |  |
| Spending a few hours in the park (1n1) | 30 | 1 | 1 |  |  | 40 | 1 | 1 |  |  |  |  | **40** |  |
| **Wisdom** | Wisdom | Daring | Success | Honesty | Unity | Varied |  | Wisdom | Daring | Success | Honesty | Unity | Varied |  |
| Giving advice (2u1) | **63** |  | 1 | 2 |  | 1 | 1 | **29** |  |  | 14 |  |  |  |
| Making important decisions (2u2) | **60** | 6 | 5 | 2 |  |  |  | **29** |  | 10 |  |  |  |  |
| Explaining something (2a1) | **65** | 1 | 1 | 7 |  |  | 1 | **37** |  | 1 | 4 |  |  |  |
| Receiving advice (2b1) | **63** | 1 | 3 | 3 |  | 2 |  | **35** |  | 2 | 7 |  |  |  |
| Solving conflicts (2b2) | **61** | 1 |  | 3 |  |  | 2 | 19 |  | 1 | 20 |  |  |  |
| Dealing with awkward situations (2b3) | 31 | 22 |  | 1 |  | 12 | 4 | 15 | 6 |  | 16 |  | 3 | 1 |
| **A World of Beauty** | Power | Broad | Security | Obedience | Beauty | Wisdom |  | Power | Broad | Security | Obedience | Beauty | Wisdom |  |
| Beauty pageants (3u1) | 7 | 2 | 2 | 1 | **51** | 2 | 5 | 7 | 1 |  | 2 | **28** |  | 5 |
| Walking outside (3u2) | 3 | 16 | 3 | 1 | **39** | 4 | 1 | 1 |  |  |  | **38** |  | 1 |
| Having fun with friends (3b1) | 13 | 26 | 1 | 1 | 14 | 5 | 12 | 8 | 17 | 6 |  | 3 |  | 9 |
| Enjoying life with close relatives (3b2) | 2 |  | **56** | 3 | 3 | 9 | 4 |  |  | **42** |  | 1 |  |  |
| Taking care of one’s own body (3b3) | 1 | 4 | 3 | 5 | 31 | 24 | 5 | 1 | 2 | 3 | 5 | 12 | 13 | 4 |
| Enjoying an art museum (3n1) | 1 | 14 |  |  | **43** | 9 |  |  | 5 |  |  | **35** |  | 1 |
| **Social Justice** | Wealth | Justice | Loyalty | Power | Ambition | Wisdom |  | Wealth | Justice | Loyalty | Power | Ambition | Wisdom |  |
| Gay rights (4u1) | 2 | **57** | 2 | 4 |  | 2 | 10 |  | **35** | 1 | 3 | 1 | 3 |  |
| Ensuring that equity is applied equally to all (4u2) | | **65** | 3 | 3 |  | 1 | 1 | 1 | **35** |  | 2 |  |  | 1 |
| Public services (4b1) | 1 | **49** | 1 | 14 |  | 1 | 2 | 1 | **32** |  | 6 | 1 |  | 2 |
| Fighting against prejudices and crimes (4b2) |  | **68** |  | 2 |  |  |  |  | **44** |  |  |  |  |  |
| Fighting against difficulties in the society (4b3) | 1 | **58** | 2 | 3 | 2 | 1 |  |  | **39** |  |  | 1 |  |  |
| Ensuring animal rights (4n1) |  | **39** | 12 | 1 |  | 15 | 4 |  | **37** | 1 |  |  | 2 | 1 |
| **Broad-mindedness** | Protect | Justice | Freedom | Daring | Broad | Honesty |  | Protect | Justice | Freedom | Daring | Broad | Honesty |  |
| Meeting new people (5u1) |  | 1 | 38 | 1 | 29 | 1 |  |  |  | 5 | 5 | **32** | 1 |  |
| Living in the moment (5b1) |  |  | **47** | 8 | 11 | 1 |  |  |  | 17 | 20 | 3 |  |  |
| Reducing prejudices (5b2) |  | 34 | 15 |  | 20 | 1 |  |  | 19 | 3 |  | 20 |  |  |
| Accepting other social orientations (5b3) |  | 8 | 6 |  | **53** |  | 1 |  | 3 | 2 |  | **39** |  |  |
| Approaching strangers (5b1) |  | 2 | 13 | 14 | **39** | 1 | 4 |  |  | 4 | 13 | 22 |  |  |
| Reading authors with different political views (5n2) | | 2 | 8 |  | **66** |  | 1 |  | 1 | 4 |  | **36** |  |  |
| **Protecting the environment** | Varied | Helpfulness | Wealth | Protect | Unity | Daring |  | Varied | Helpfulness | Wealth | Protect | Unity | Daring |  |
| Putting certain rubbish in recycle bins rather than general waste* (6u1) | | 9 |  | **54** | 3 | 1 |  |  | 1 |  | **42** |  |  |  |
| Making sure the lights are off (6u2) | 3 | 21 | 4 | **36** | 7 | 1 | 5 |  | 1 |  | **39** |  |  |  |
| Walk instead of using car for short distances (6u3) | 8 | 2 | 1 | **48** | 6 |  |  |  |  |  | **38** | 4 |  |  |
| Throwing garbage in the bin (6a1) |  |  |  | **61** | 8 | 1 | 1 |  | 4 |  | **36** | 3 |  | 1 |
| Saving water* (6b1) |  | 6 | 3 | **52** | 11 |  |  |  | 1 | 1 | **35** | 3 |  |  |
| Installing heat insulation in the house* (6n1) | 10 | 8 | 25 | 11 |  | 3 | 13 | 1 | 4 | 8 | **25** |  |  | 2 |
| **Equality** | Obedience | Creativity | Equality | Protect | Discipline | Security |  | Obedience | Creativity | Equality | Protect | Discipline | Security |  |
| Allowing all children to use all equipment (7u1) | 5 | 8 | 20 | 1 | 10 | 12 | 14 | 4 | 5 | **22** |  | 6 | 2 | 4 |
| Fair evaluation of job applications (7u2) | 2 | 5 | 34 | 1 | 22 | 4 | 4 |  |  | **39** |  | 1 |  |  |
| Fair treatment in the work place (7u3) | 10 |  | **46** | 1 | 16 |  |  |  |  | **42** |  |  |  |  |
| Treating everyone in the same way without  regard to their sex (7b1) | | | **66** |  |  |  |  |  |  | **44** |  |  |  |  |
| University quotas for blacks and Indians (7b2) | 2 |  | **65** |  | 1 |  | 9 |  |  | **39** |  |  |  | 1 |
| Discrimination against left-handed people (7n1) |  | 2 | 22 |  | 10 |  | 31 | 1 | 1 | 17 |  |  |  | 22 |
| **Freedom** | Honesty | Success | Helpfulness | Pleasure | Protect | Freedom | ? | Honesty | Success | Helpfulness | Pleasure | Protect | Freedom | ? |
| The right to vote (8u1) | 4 |  | 11 | 2 |  | **47** | 3 |  |  | 1 |  |  | **39** | 3 |
| Having the possibility to choose (8u2) |  | 3 | 1 | 1 | 1 | **67** |  |  |  |  |  |  | **40** |  |
| Knowing people from different places (8b1) |  | 1 | 2 | 30 |  | 41 | 1 |  | 2 | 5 | 1 |  | **28** | 6 |
| Defending one’s own opinion (8b2) | 7 | 1 | 2 |  |  | **62** |  | 6 | 2 |  |  |  | **34** | 2 |
| Traveling (8b3) |  | 1 |  | **56** | 1 | 9 |  |  |  |  | 10 |  | **30** |  |
| Questioning religious authorities (8n1) | 1 |  | 3 | 3 | 1 | **47** | 14 | 6 |  |  |  |  | **32** | 3 |
| **Creativity** | Discipline | Tradition | Varied | Obedience | Protect | Creativity |  | Discipline | Tradition | Varied | Obedience | Protect | Creativity |  |
| Making art (9u1) | 1 | 2 | 4 | 1 | 1 | **60** | 1 |  |  |  |  |  | **43** |  |
| Painting (9u2) | 1 |  | 5 |  | 1 | **60** |  |  |  |  |  |  | **40** |  |
| Being original at work (9b1) | 24 | 2 | 2 | 5 | 1 | 36 | 2 |  |  |  |  |  | **40** | 1 |
| Companies who aim to prosper (9b2) | 30 | 4 | 1 | 4 |  | 27 | 10 | 16 | 1 |  | 2 | 1 | 16 | 8 |
| Solving problems (9a1) | 27 | 1 | 3 | 3 | 2 | 31 | 6 | 8 |  | 1 | 2 |  | **26** | 2 |
| Thinking of new ideas (9a2) |  |  | 5 |  | 1 | **60** |  |  |  |  |  |  | **41** |  |
| **A varied life** | Success | Justice | Tradition | Pleasure | Broad | Varied |  | Success | Justice | Tradition | Pleasure | Broad | Varied |  |
| Maintaining a good work life balance (10u1) | 49 |  |  | 13 |  | 4 | 1 | 5 |  |  | 3 |  | **34** | 1 |
| Gaining new experiences (10u2) | 22 |  |  | 10 | 11 | 33 |  |  |  |  | 1 | 8 | **31** |  |
| Trying different activities (10a1) | 18 | 1 |  | 11 | 5 | 30 | 2 |  |  |  |  | 13 | **27** | 1 |
| Trying new food (10b1) |  |  | 2 | 30 | 10 | 30 | 1 |  |  |  | 3 | 16 | 25 |  |
| Trying to learn new traditions (10b2) | 1 |  | 15 | 5 | **36** | 13 | 1 |  | 1 | 14 |  | 20 | 5 |  |
| Practicing different sports (10b3) | 1 |  |  | 25 | 2 | 39 |  | 2 |  |  | 7 | 1 | **31** |  |
| **Daring** | Obedience | Wisdom | Tradition | Honesty | Beauty | Daring |  | Obedience | Wisdom | Tradition | Honesty | Beauty | Daring |  |
| Doing extreme sports (11u1) |  | 5 | 1 | 1 | 3 | **56** |  |  |  |  |  |  | **42** |  |
| Risk-taking (11u2) | 1 | 1 | 2 |  | 2 | **63** | 3 |  |  |  |  |  | **40** |  |
| Taking a test without studying (11b1) | 1 | 8 |  |  | 1 | **55** | 8 |  | 1 |  |  |  | **37** | 4 |
| Leaving home to study in another city (11b2) |  | 14 |  |  | 1 | **50** | 2 |  | 4 |  |  | 3 | **37** |  |
| Participating in an experiment that was not approved by an ethics committee (11n1) | 1 | 1 | 8 | 1 |  | **49** | 16 | 9 |  | 1 |  |  | **26** | 4 |
| Living in a house without a smoke detector* (11n2) | 1 | 3 | 5 | 1 | 1 | 21 | 35 | 1 | 4 | 1 |  |  | **27** | 8 |
| **Pleasure** | Security | Pleasure | Varied | Freedom | Discipline | Power |  | Security | Pleasure | Varied | Freedom | Discipline | Power |  |
| Enjoy a fulfilling sexual relationship (12u1) | 1 | **64** |  | 1 | 1 |  |  | 2 | **38** |  | 3 |  |  |  |
| Having a drink (12u2) |  | **51** | 8 | 10 | 1 | 1 | 1 |  | **36** | 1 | 1 | 1 | 1 |  |
| Spending time with close relatives and friends (12a1) | 19 | **48** | 4 | 3 | 1 | 1 |  | **34** | 7 |  |  |  |  | 1 |
| Relief of stress from everyday life (12a2) | 6 | **54** | 3 | 1 | 4 | 1 | 3 |  | **35** | 6 | 3 |  |  |  |
| Being in the presence of nice people (12b1) | 28 | 31 | 3 | 1 | 2 | 1 | 1 | 2 | **36** | 1 |  |  | 1 |  |
| Watching a football game 12b2) |  | **61** | 5 | 2 |  |  | 2 |  | **33** | 1 |  | 1 | 1 | 5 |
| **Success** | Discipline | Creativity | Success | Beauty | Protect | Power |  | Discipline | Creativity | Success | Beauty | Protect | Power |  |
| Striving for top grades (13u1) | 30 | 2 | 37 | 1 |  |  |  | 14 |  | **29** |  |  |  |  |
| Aiming to be good in a job (13u2) | 19 | 3 | **42** |  |  | 3 |  | 2 | 1 | **37** |  |  |  |  |
| Achieving goals (13u3) | 9 | 1 | **59** |  |  | 3 |  | 11 |  | **31** |  |  |  |  |
| Passing an entrance exam for a job (13b1) | 10 |  | **63** |  |  | 2 |  | 11 |  | **33** |  |  |  |  |
| Being able to meet objectives (13b2) | 11 | 4 | **57** |  |  |  | 1 | 20 |  | 20 |  |  |  |  |
| Obtaining a PhD (13b3) | 9 | 1 | **52** |  |  | 6 |  | 11 |  | **29** | 1 |  |  |  |
| **Ambition** | Ambition | Beauty | Helpfulness | Loyalty | Daring | Wealth |  | Ambition | Beauty | Helpfulness | Loyalty | Daring | Wealth |  |
| Doing good work in order to obtain a promotion (14u1) | **59** |  | 3 |  | 2 | 2 |  | **40** |  | 1 | 1 |  | 1 |  |
| Applying for a new job (14u2) | 29 |  | 16 | 1 | 15 | 4 | 8 | **39** |  |  |  |  | 1 |  |
| Working to achieve your goals (14u3) | **53** |  | 9 | 2 | 3 | 7 | 2 | **40** |  |  | 1 |  |  |  |
| Having children, friends and family (14b1) | 2 | 10 | 5 | 28 | 1 | 19 | 7 | 4 | 7 | 1 | 26 |  |  | 6 |
| Getting the top job (14b2) | **49** |  | 7 | 1 | 4 | 5 | 1 | **38** |  |  |  |  | 2 |  |
| Wanting something what no one else has (14b3) | **55** | 1 |  |  | 2 | 9 | 3 | **30** |  |  |  | 1 | 10 |  |
| **Wealth** | Obedience | Varied | Wisdom | Security | Wealth | Daring |  | Obedience | Varied | Wisdom | Security | Wealth | Daring |  |
| Buying a house (15u1) |  |  | 6 | **45** | 17 |  | 2 |  |  |  | 24 | 19 |  |  |
| Going shopping (15u2) | 1 | 11 |  | 3 | **46** | 2 | 4 | 1 | 5 |  | 1 | **32** |  | 1 |
| Provide children with what they need (15a1) | 1 | 3 | 33 | 29 | 3 |  | 2 |  | 1 | 1 | **35** | 5 |  |  |
| Having many good friends * (15b1) | 1 | 20 | 33 | 6 | 11 |  | 5 | 2 | **25** | 1 | 5 |  |  | 11 |
| Being able to celebrate life (15b2) |  | 24 | 30 | 7 | 5 | 2 | 5 |  | **30** | 4 | 5 |  |  | 1 |
| Being able to buy organic food* (15n1) |  | **29** | 15 | 6 | 4 |  | 12 |  | 5 | 3 |  | **25** |  | 8 |
| **Social Power** | Success | Unity | Power | Protect | Wealth | Varied |  | Success | Unity | Power | Protect | Wealth | Varied |  |
| Making decisions which are followed by other people (16u1) | 5 |  | **41** |  |  | 7 | 23 | 1 |  | **41** |  |  | 1 |  |
| Trying to persuade people to vote for you (16u2) | 3 | 1 | **58** |  | 1 | 2 | 8 | 2 |  | **37** |  |  |  |  |
| Providing welfare to society (16b1) | 7 | 2 | **29** | 4 | 1 | 8 | 13 | 4 |  | 18 | 3 | 3 | 2 | 12 |
| Strengthening the rights of citizens (16b2) | 7 | 3 | **48** |  |  | 2 | 9 | 4 | 1 | **32** |  |  |  | 7 |
| Being able to make improvements within a community (16b3) | 7 | 1 | **49** | 2 | 4 | 1 | 3 | 7 | 3 | 14 | 10 | 2 | 1 | 3 |
| Controlling other people (16n1) |  |  | **59** |  | 5 | 2 | 6 | 1 |  | **38** |  |  |  | 2 |
| **Family Security** | Helpfulness | Justice | Ambition | Creativity | Security | Beauty |  | Helpfulness | Justice | Ambition | Creativity | Security | Beauty |  |
| Comforting close relatives (17u1) | 37 | 6 | 1 | 1 | 25 |  | 1 | 8 |  |  |  | **34** |  |  |
| Keeping watch over the children (17u2) | 34 | 4 | 1 |  | 27 |  | 1 | 5 | 1 |  |  | **34** |  |  |
| Providing a stable home life (17u3) | 7 |  | 2 |  | **59** |  | 1 | 2 |  |  |  | **40** |  |  |
| Leisure time on the beach with close relatives (17b1) | 9 | 1 |  | 6 | 18 | 28 | 6 |  |  |  |  | **28** | 14 | 2 |
| Installing electric fences for your home* (17b2) | 1 | 1 |  | 2 | **69** |  |  |  | 1 |  | 1 | **36** |  | 2 |
| Ensuring a good income (17b3) | 6 | 4 | 38 |  | 25 | 2 | 2 | 1 |  | 20 |  | 20 |  |  |
| **Respect for Traditon** | Tradition | Pleasure | Helpfulness | Beauty | Success | Creativity |  | Tradition | Pleasure | Helpfulness | Beauty | Success | Creativity |  |
| Celebrating religious ceremonies (18a1) | **60** | 10 | 1 | 2 |  |  | 2 | **42** |  |  |  |  |  |  |
| Spending time with family at Christmas (18a2) | 39 | 29 | 3 |  | 2 |  |  | **29** | 10 |  | 1 |  |  |  |
| Honouring your parents’ requests (18b1) | **53** |  | 12 | 1 | 1 |  |  | **30** |  | 10 |  |  |  | 2 |
| Listening to your father’s advice (18b2) | **53** | 2 | 8 | 1 | 5 |  |  | 22 |  | 11 |  | 6 | 1 | 3 |
| Celebrating the anniversary of the city in which you live (18b3) | **63** | 2 | 1 |  | 1 |  |  | **35** |  |  | 4 | 1 |  |  |
| Celebrating a carnival (18n1) | 23 | 35 |  | 5 |  |  | 8 | 19 | 19 |  | 1 |  | 2 |  |
| **Self-discipline** | Freedom | Honesty | Power | Creativity | Discipline | Wealth |  | Freedom | Honesty | Power | Creativity | Discipline | Wealth |  |
| Exercising (19u1) | 23 |  |  | 5 | 37 | 1 | 1 | 2 |  |  |  | **38** | 1 | 2 |
| Losing weight (19u2) | 10 |  |  | 3 | **51** | 1 | 4 | 1 | 1 |  |  | **38** |  |  |
| Quitting smoking (19u3) | 8 |  |  | 1 | **55** | 1 | 3 |  |  |  |  | **41** |  |  |
| Succeeding in your professional career (19a1) | 1 | 8 | 8 | 4 | **32** | 18 | 1 | 1 |  | 1 | 3 | **28** | 9 |  |
| Doing all your work (19b1) | 1 | 8 |  | 2 | **63** | 1 | 1 |  |  |  |  | **40** |  |  |
| Living your own life and not following the crowd (19n1) | 1 | 8 |  | 2 | **63** | 1 | 1 | **33** | 1 |  | 1 | 5 |  |  |
| **Obedience** | Wisdom | Obedience | Freedom | Discipline | Tradition | Equality |  | Wisdom | Obedience | Freedom | Discipline | Tradition | Equality |  |
| Complying with the law* (20a1) | 6 | **46** |  | 11 | 5 | 3 |  |  | **39** |  | 2 |  | 2 |  |
| Children need to comply (20a2) | 5 | **49** |  | 13 | 6 |  | 1 |  | **38** |  |  | 2 |  |  |
| A high-ranking person telling inferiors what to do (20a3) | 8 | 33 | 3 | 5 | 5 |  | 19 | 3 | **32** |  | 1 | 1 | 1 | 3 |
| Listening to older people (20b1) | 26 | 27 |  |  |  | 13 |  | 31 | 5 |  |  | 7 | 1 |  |
| Attending classes and following the rules (20b2) | 5 | **41** | 1 | 19 | 2 | 1 |  | 1 | **30** |  | 9 |  |  |  |
| Helping your mother if she asks for it (20b3) | 2 | **60** |  | 1 | 2 | 2 |  | 1 | **30** | 1 |  | 5 | 2 | 2 |
| **Helpfulness** | Broad | Varied | Justice | Helpfulness | Tradition | Creativity |  | Broad | Varied | Justice | Helpfulness | Tradition | Creativity |  |
| Customer service (21u1) | 2 | 1 | **43** | 15 | 1 |  | 9 | 1 |  | 3 | **35** |  |  | 3 |
| Supporting other people (21a1) | 1 | 1 | 4 | **59** | 2 |  |  | 4 |  | 6 | **30** |  |  |  |
| Supporting colleagues (21a2) | 3 |  |  | **63** | 2 |  | 1 | 1 |  | 2 | **38** | 1 |  |  |
| Offering a lift (21b1) | 5 | 17 |  | **35** | 1 | 6 | 3 |  |  |  | **43** | 1 |  |  |
| Giving food to poor people (21b2) | 3 | 1 | 25 | **43** |  |  |  | 1 |  | 11 | **28** |  |  |  |
| Accepting a gift (21n1) | 17 | 7 |  | 14 | 18 | 1 | 19 | 5 | 2 | 2 | 1 | 16 | 1 | 13 |
| **Loyalty** | Creativity | Obedience | Protect | Loyalty | Varied | Helpfulness |  | Creativity | Obedience | Protect | Loyalty | Varied | Helpfulness |  |
| Supporting your rugby team (22u1) |  |  |  | **38** | 18 | 3 | 18 |  |  |  | **41** |  |  | 2 |
| Defending a colleague (22u2) |  |  |  | **56** | 1 | 14 | 2 |  | 1 |  | **36** |  | 4 |  |
| Keeping secrets* (22u3) | 1 | 3 |  | **59** | 3 | 1 |  |  |  |  | **39** |  |  | 3 |
| Standing by your friends (22a1) |  |  |  | **47** |  | 22 |  |  |  |  | **44** |  |  |  |
| A husband who respects his wife (22b1) |  | 3 |  | **61** |  | 2 |  |  |  |  | **39** |  |  | 1 |
| Always working for the same company (22b2) | 1 | 4 | 1 | **49** | 1 | 8 | 7 |  | 3 |  | **38** |  |  |  |
| **Honesty** | Justice | Wisdom | Honesty | Discipline | Power | Pleasure |  | Justice | Wisdom | Honesty | Discipline | Power | Pleasure |  |
| Not cheating (23u1) | 1 | 6 | **56** | 10 |  |  |  | 2 | 1 | **34** | 6 |  |  |  |
| Telling the truth (23a1) |  | 3 | **61** | 2 |  |  |  |  |  | **40** |  |  |  |  |
| Returning money which you have found or wrongly received (23b1)^ | 3 |  | **65** |  |  |  | 1 |  |  |  |  |  |  |  |
| Borrowing money and giving it back (23b2) | 2 | 1 | **62** | 2 |  |  |  | 5 |  | **73** | 4 |  |  | 3 |
| Completing an exam without cheating* (23n1) |  | 7 | **56** | 7 |  | 1 |  |  |  | **40** |  |  |  |  |
| Informing a car owner when you have accidentally damaged his/her car (23n2) | 6 | 1 | **70** |  |  |  |  | 1 |  | **39** |  |  |  |  |

*Note*. Most frequent chosen value in bold if significant different from the second most often chosen value. ?: “Don’t know”. Unity: Unity with nature, Beauty: A world of beauty, Protect: Protecting the environment, Justice: Social Justice, Broad: Broad-mindedness, Varied: A varied life, Power: Social power, Security: Family security, Tradition: Respect for Tradition, Discipline: Self-discipline.

1u1 is the first instantiation mentioned by British participants of the first value (here: Unity with nature), 2b3 is the third instantiation mentioned by Brazilian participants of the second value. The letter “a” in the middle stands for all (i.e., mentioned in all two countries), the letter “n” indicates that the instantiation was not mentioned by the participants in none of the two countries (i.e., was created by us).
